# Supplementary material for: Characterization of meiotic axis proteins in the model brown alga Ectocarpus
Source: EMBO Rep. 2025 Oct 23;26(23):5673–702. doi: 10.1038/s44319-025-00605-3 (PMC12678776; doi:10.1038/s44319-025-00605-3)
Supplement: Supplementary file 1 — Table EV1 [file 44319_2025_605_MOESM1_ESM.docx]

**Table EV1.** Oligonucleotide primers for ecHOP1 RT-PCR

| **Primers** | **Orientation** | **Nucleotide Sequence** |
| --- | --- | --- |
| ecHOP1^1500-1855bp^ | Sense | 5’-GGACGACTCGGAAACTCAAGT-3’ |
|  | Antisense | 3’-CGCTGTTGTTGTTGTCATCGT-5’ |
| ecHOP1^1067-1529bp^ | Sense | 5’-GCATGATGACCAACCTCAACG-3’ |
|  | Antisense | 3’-AGATTATCGTCGCCACCATCG-5’ |
| ecHOP1^1302-1520/1306-1577bp^ | Sense | 5’-CCTGGAGAAGATGGTTCACGA-3’ |
|  | Antisense | 3’-ACTTGAGTTTCCGAGTCGTCC-5’ |
