# Supplementary material for: Characterization of meiotic axis proteins in the model brown alga Ectocarpus
Source: EMBO Rep. 2025 Oct 23;26(23):5673–702. doi: 10.1038/s44319-025-00605-3 (PMC12678776; doi:10.1038/s44319-025-00605-3)
Supplement: Supplementary file 2 — Table EV2 [file 44319_2025_605_MOESM2_ESM.docx]

**Table EV2.** Oligonucleotide primers for ecHOP1 and ecRED1 Cloning

| **Primers** | **Orientation** | **Nucleotide Sequence** |
| --- | --- | --- |
| ecHOP1^1-274^ | Sense | 5’-CTGAGACTCAAGCGCTTACACTTGTTAAGAAC-3’ |
|  | Antisense | 3’-TCCTCTAGTACTTCTCGACAAGCTTTTACTGTTCCTGGGTGTCATAACC-5’ |
| ecHOP1^50-274^ | Sense | 5’-CTGTTCCAGGGGCCCGGATCCATGCAATCACTTACACTTGTTAAG-3’ |
|  | Antisense | 3’-TCCTCTAGTACTTCTCGACAAGCTTTTACTGTTCCTGGGTGTCATAACC-5’ |
| ecHOP1^374-495^ | Sense | 5’-CTGTTCCAGGGGCCCGGATCCGGACACCACCAAGCGTTTAAAGCACAG-3’ |
|  | Antisense | 3’-TCCTCTAGTACTTCTCGACAAGCTTTTATCACTCCTCAGGCTTGTCGATAATTAC-5’ |
| ecHOP1^R432A^ | Sense | 5’-CGCGAATTAGCCGCCGAGTTCCTGGAAAAAATG-3’ |
|  | Antisense | 3’-CATTTTTTCCAGGAACTCGGCGGCTAATTCGCG-5’ |
| ecHOP1^1-597; iso2^ | Sense | 5’-CTGAGACTCAAGCGCTTACACTTGTTAAGAAC-3’ |
|  | Antisense | 3’-TCCTCTAGTACTTCTCGACAAGCTTTTATCATCAGCGAATCTTCTCTTGCACACG-5’ |
| ecHOP1^562-694, WT^ | Sense | 5’-CTGTTCCAGGGGCCCGGATCCATGCCACCTATTCCC-3’ |
|  | Antisense | 3’-TCCTCTAGTACTTCTCGACAAGCTTTTATCACTCTTCACAGACACGAAGAGC-5’ |
| ecHOP1^562-694, mut^ | Sense | 5’-CTGTTCCAGGGGCCCGGATCCCCACCTATTCCCACGCCCAGCGCACCTCCC-3’ |
|  | Antisense | 3’-TCCTCTAGTACTTCTCGACAAGCTTTTATCACTCTTCACAGACACGAAGAGC-5’ |
| ecHOP1^562-620, WT^ | Sense | 5’-CTGTTCCAGGGGCCCGGATCCATGCCACCTATTCCC-3’ |
|  | Antisense | 3’-TCCTCTAGTACTTCTCGACAAGCTTTTATCATTCATCTGAATTGTTGTTATC-5’ |
| ecHOP1^562-620, mut^ | Sense | 5’-TCCTCTAGTACTTCTCGACAAGCTTTTATCATTCATCTGAATTGTTGTTATC-3’ |
|  | Antisense | 3’-GATAACAACAATTCAGATGAATGATAAAAGCTTGTCGAGAAGTACTAGAGGA-5’ |
| ecHOP1^650-694, WT^ | Sense | 5’-CTGTTCCAGGGGCCCGGATCCATGCCCGGGTATGAGCGCGTAATG-3’ |
|  | Antisense | 5’-TCCTCTAGTACTTCTCGACAAGCTTTTATCACTCTTCACAGACACGAAGAGC-3’ |
| ecHOP1^650-694, mut^ | Sense | 5’-CTGTTCCAGGGGCCCGGATCCGGGTATGAGCGCGTCATGTTG-3’ |
|  | Antisense | 3’-CAACATGACGCGCTCATACCCGGATCCGGGCCCCTGGAACAG-5’ |
| ecHOP1^560-597, WT^ | Sense | 5’-CTGTTCCAGGGGCCCGGATCCGGTCAAGTTGGACGTCATACACCAGAG-3’ |
|  | Antisense | 3’-TCCTCTAGTACTTCTCGACAAGCTTTTATCATCAGCGAATCTTCTCTTGCACACG-5’ |
| ecHOP1^560-597, mut^ | Sense | 5’-CTGTTCCAGGGGCCCGGATCCGGTCAAGTTGGACGTCATACACCAGAG-3’ |
|  | Antisense | 3’-CTCTGGTGTATGACGTCCAACTTGACCGGATCCGGGCCCCTGGAACAG-5’ |
| ecRED1^1-510^ | Sense | 5’-TCCTCTAGTACTTCTCGACAAGCTTTACTCTGGGTCGTACTCTCCGTCTTCCCC-3’ |
|  | Antisense | 3’-TCCTCTAGTACTTCTCGACAAGCTTTACTAACTGCTGGCTTTCTCGCGCGATGC-5’ |
| ecRED1^CM-R-A^ | Sense | 5’-CTGTTCCAGGGGCCCGGATCCATGGAAGATTTGGATGATGACGATGAAGAAGG-3’ |
|  | Antisense | 3’-TCCTCTAGTACTTCTCGACAAGCTTTACTAACTGCTGGCTTTCTCGCGCGATGC-5’ |
| ecRED1^546-602^ | Sense | 5’-CTGTTCCAGGGGCCCGGATCCATGGCGCGGAAAAGAAGCGTATTACAGAC-3’ |
|  | Antisense | 3’-TCCTCTAGTACTTCTCGACAAGCTTTACTAATCAGCGTCGTCCGAAAAGGAACG-5’ |
| ecRED1^611-641^ | Sense | 5’-CTGTTCCAGGGGCCCGGATCCATGGGTGGAGATTCCTGGATGGCACCC3’ |
|  | Antisense | 3’-TCCTCTAGTACTTCTCGACAAGCTTTACGGACCCTGTTTTAGTGCGAGC-5’ |
| ecRED1^665-718^ | Sense | 5’-CTGTTCCAGGGGCCCGGATCCATGTCAACCGAATGGGATATTTCTGAAGAC-3’ |
|  | Antisense | 3’-TCCTCTAGTACTTCTCGACAAGCTTTACTACGAACCTGGACCGGGCCCGGC-5’ |
| ecRED1^778-826^ | Sense | 5’-CTGTTCCAGGGGCCCGGATCCATGGGTTTGGATGAAGATCAGAAGCAG-3’ |
|  | Antisense | 3’-TCCTCTAGTACTTCTCGACAAGCTTTACTAACGCCCTGAGCCCGAAGAGCTTCC-5’ |
| ecRED1^917-965^ | Sense | 5’-CTGTTCCAGGGGCCCGGATCCATGGCGGCAGCTACTGGATCTCGTGAGGAG-3’ |
|  | Antisense | 3’-TCCTCTAGTACTTCTCGACAAGCTTTACTACTCGTCGCCACCAAAGGA-5’ |
