# Supplementary material for: Characterization of meiotic axis proteins in the model brown alga Ectocarpus
Source: EMBO Rep. 2025 Oct 23;26(23):5673–702. doi: 10.1038/s44319-025-00605-3 (PMC12678776; doi:10.1038/s44319-025-00605-3)
Supplement: Supplementary file 3 — Table EV3 [file 44319_2025_605_MOESM3_ESM.docx]

**Table EV3.** Expression Vectors

| **Plasmid ID** | **Protein ID** | **Domain Boundaries** | **Fusion Tag** | **Reference** |
| --- | --- | --- | --- | --- |
| pWL1556 | ecHOP1; isoform 1 | 1-694  (Full-Length) | StrepII | This Study |
| pWL1776 | ecHOP1; isoform 1 | 1-274  (HORMA) | StrepII | This Study |
| pWL2555 | ecHOP1; isoform 1 | 1-274  (HORMA) | His_6_SUMO | This Study |
| pWL2546 | ecHOP1; isoform 1 | 562-620  (CM-H1-A); WT | His_6_MBP | This Study |
| pWL2547 | ecHOP1; isoform 1 | 562-620  (CM-H1-A); K/RxA | His_6_MBP | This Study |
| pWL2550 | ecHOP1; isoform 1 | 650-694 (CM-H1-B); WT | His_6_MBP | This Study |
| pWL2551 | ecHOP1; isoform 1 | 650-694  (CM-H1-B); K/RxA | His_6_MBP | This Study |
| pWL2552 | ecHOP1; isoform 2 | 560-597  (CM-H2-A); WT | His_6_MBP | This Study |
| pWL2553 | ecHOP1; isoform 2 | 560-597  (CM-H2-A); K/RxA | His_6_MBP | This Study |
| pWL2557 | ecHOP1; isoform 1 | 375-462  (wHTH); WT | His_6_SUMO | This Study |
| pWL2566 | ecHOP1; isoform 1 | 375-462  (wHTH); R432A | His_6_SUMO | This Study |
| pWL2604 | ecRED1 | 1-1291  (Full-Length) | His_6_MBP | This Study |
| pWL2572 | ecRED1 | CM-R-A | His_6_MBP | This Study |
| pWL2573 | ecRED1 | 546-602 | His_6_MBP | This Study |
| pWL2574 | ecRED1 | 611-641 | His_6_MBP | This Study |
| pWL2575 | ecRED1 | 665-718 | His_6_MBP | This Study |
| pWL2576 | ecRED1 | 778-826 | His_6_MBP | This Study |
| pWL2577 | ecRED1 | 917-965 | His_6_MBP | This Study |
