# Supplementary figures and images for: Characterization of meiotic axis proteins in the model brown alga Ectocarpus
Source: EMBO Rep. 2025 Oct 23;26(23):5673–702. doi: 10.1038/s44319-025-00605-3 (PMC12678776; doi:10.1038/s44319-025-00605-3)

# Eukaryotic Supergroups

- SAR
- Archaeplastida
- Opisthokonta
- Haptista
- Cryptophyta

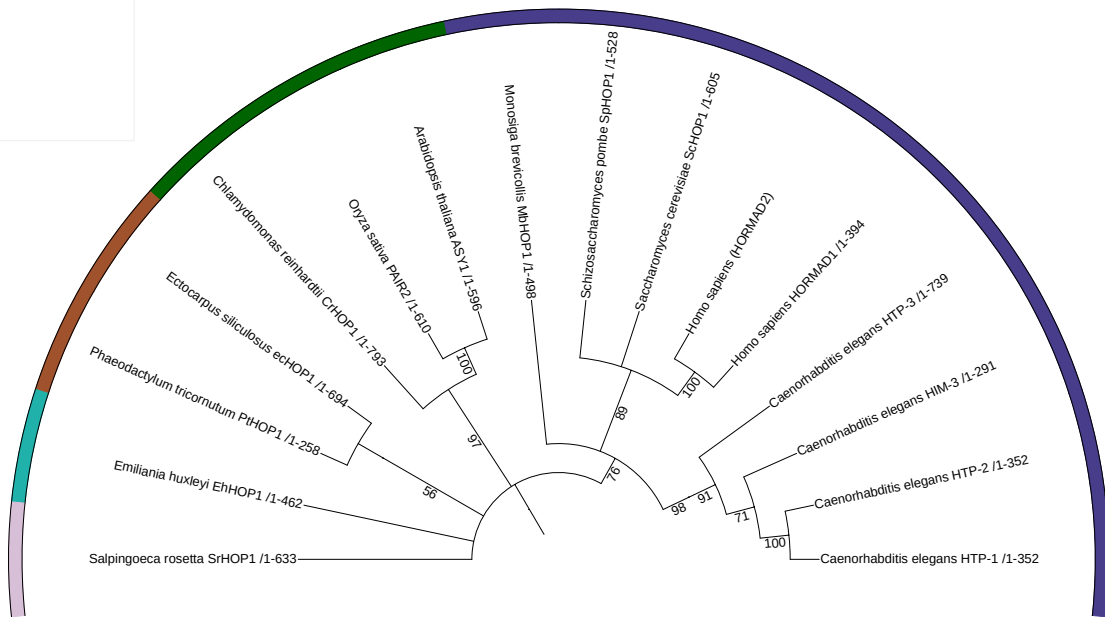

Supplement: Supplementary file 5 — Source data Fig. 1 [file 44319_2025_605_MOESM5_ESM.zip › Figure 1/1G/Figure1G_ecHOP1-phylogeny-tree.pdf]

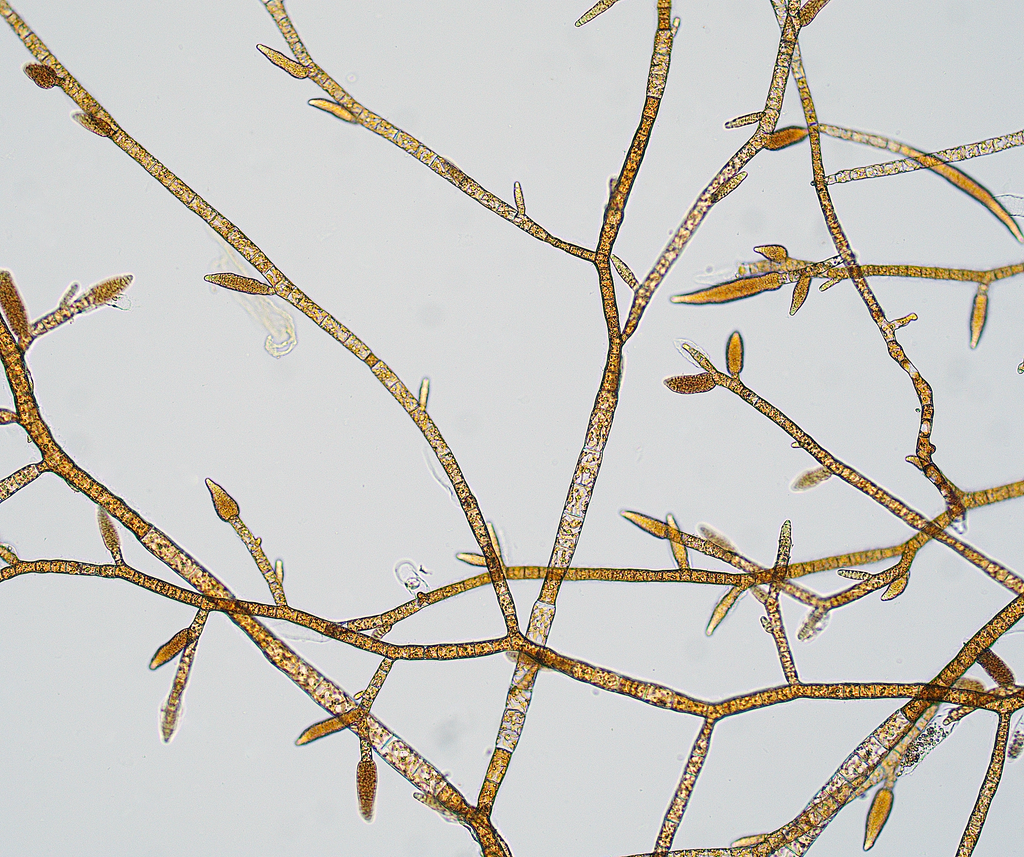

Supplement: Supplementary file 5 — Source data Fig. 1 [file 44319_2025_605_MOESM5_ESM.zip › Figure 1/1B/Figure1B_Ec25-mat-GA-producers-of-gametes-samples_14ND_x10_280922.png]

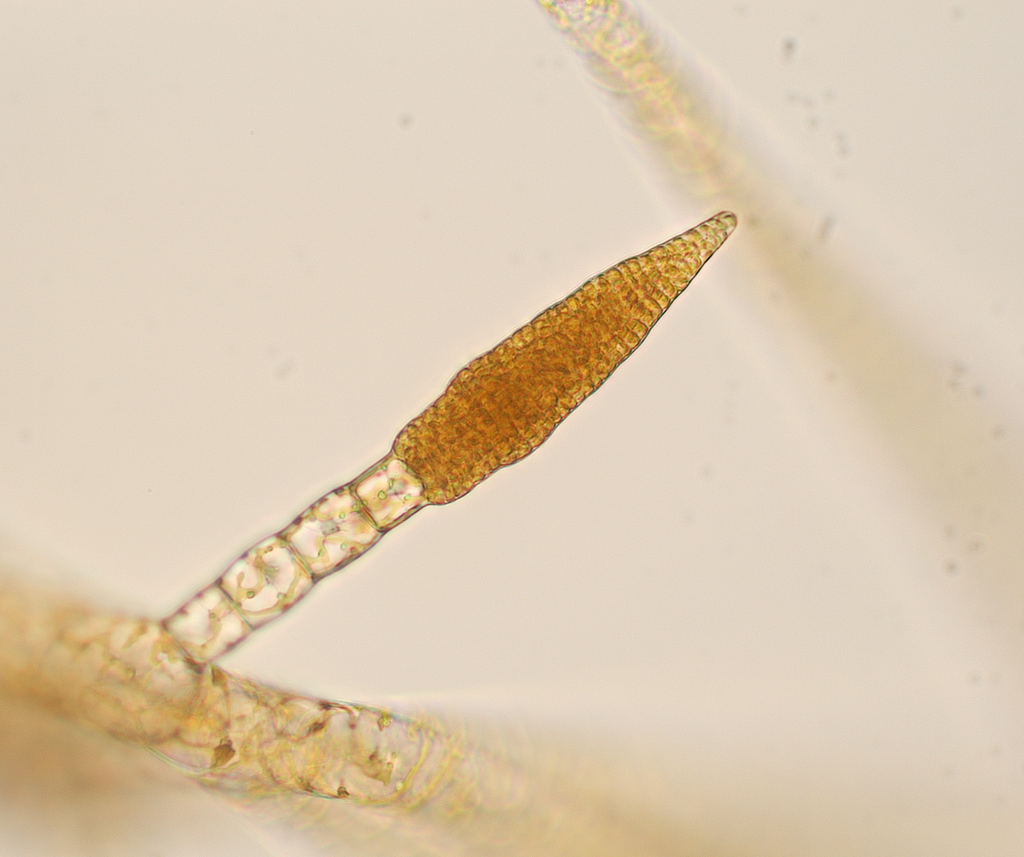

Supplement: Supplementary file 5 — Source data Fig. 1 [file 44319_2025_605_MOESM5_ESM.zip › Figure 1/1B/Figure1B_Ec32-pSP-Plurilocs-20LD_x40_4_130722.png]

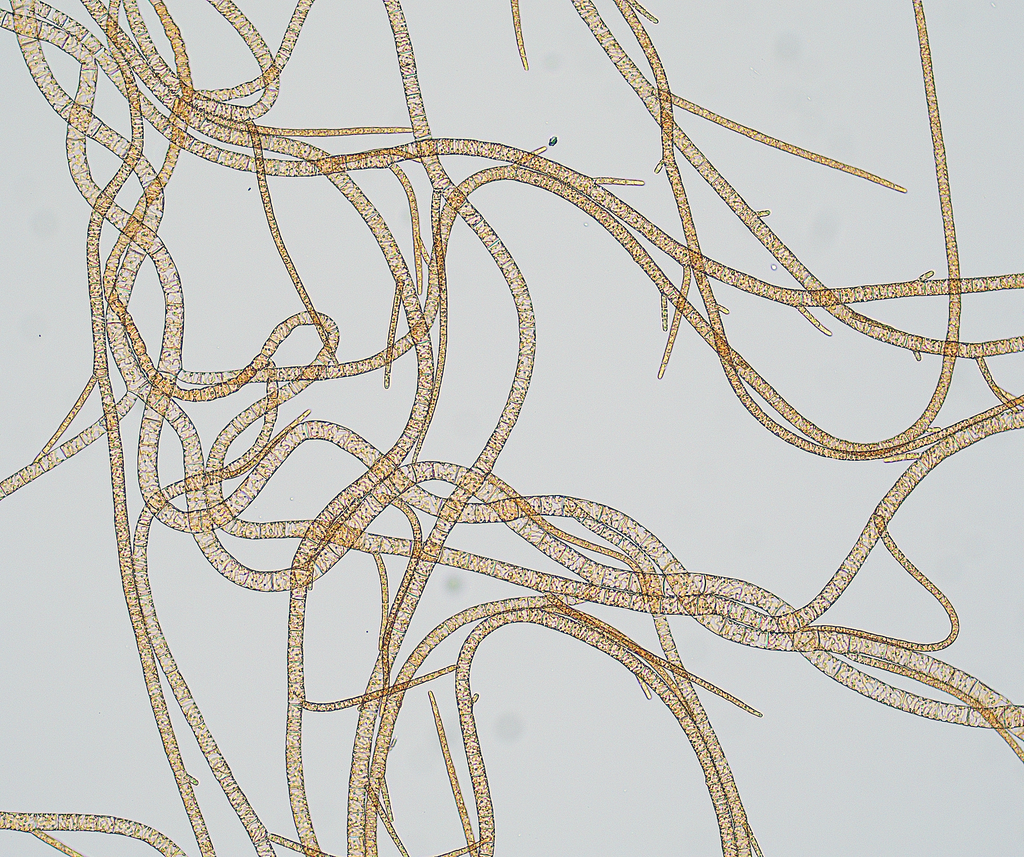

Supplement: Supplementary file 5 — Source data Fig. 1 [file 44319_2025_605_MOESM5_ESM.zip › Figure 1/1B/Figure1B_Immature-Ec32-GA-14ND_x10_151022_2.png]

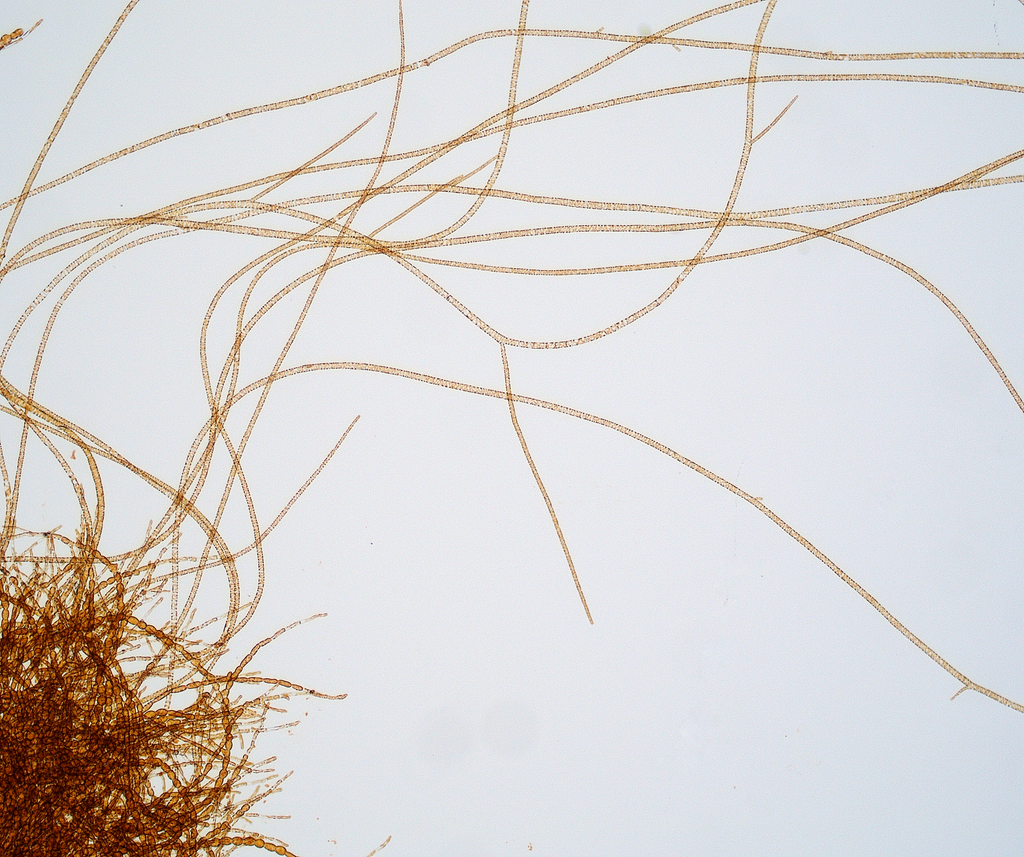

Supplement: Supplementary file 5 — Source data Fig. 1 [file 44319_2025_605_MOESM5_ESM.zip › Figure 1/1B/Figure1B_Immature-Ec32-pSP-14ND_x4_151022.png]

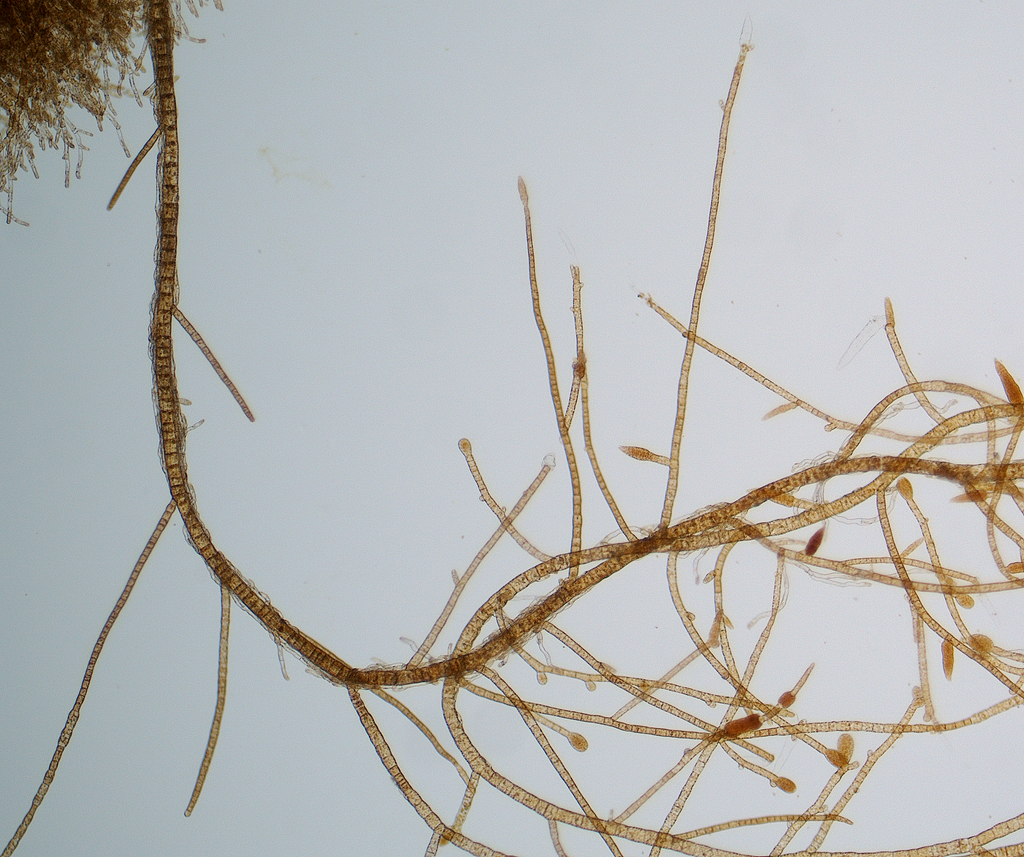

Supplement: Supplementary file 5 — Source data Fig. 1 [file 44319_2025_605_MOESM5_ESM.zip › Figure 1/1B/Figure1B_Ec17_U+P_14NDhl_x4_020922_3.png]

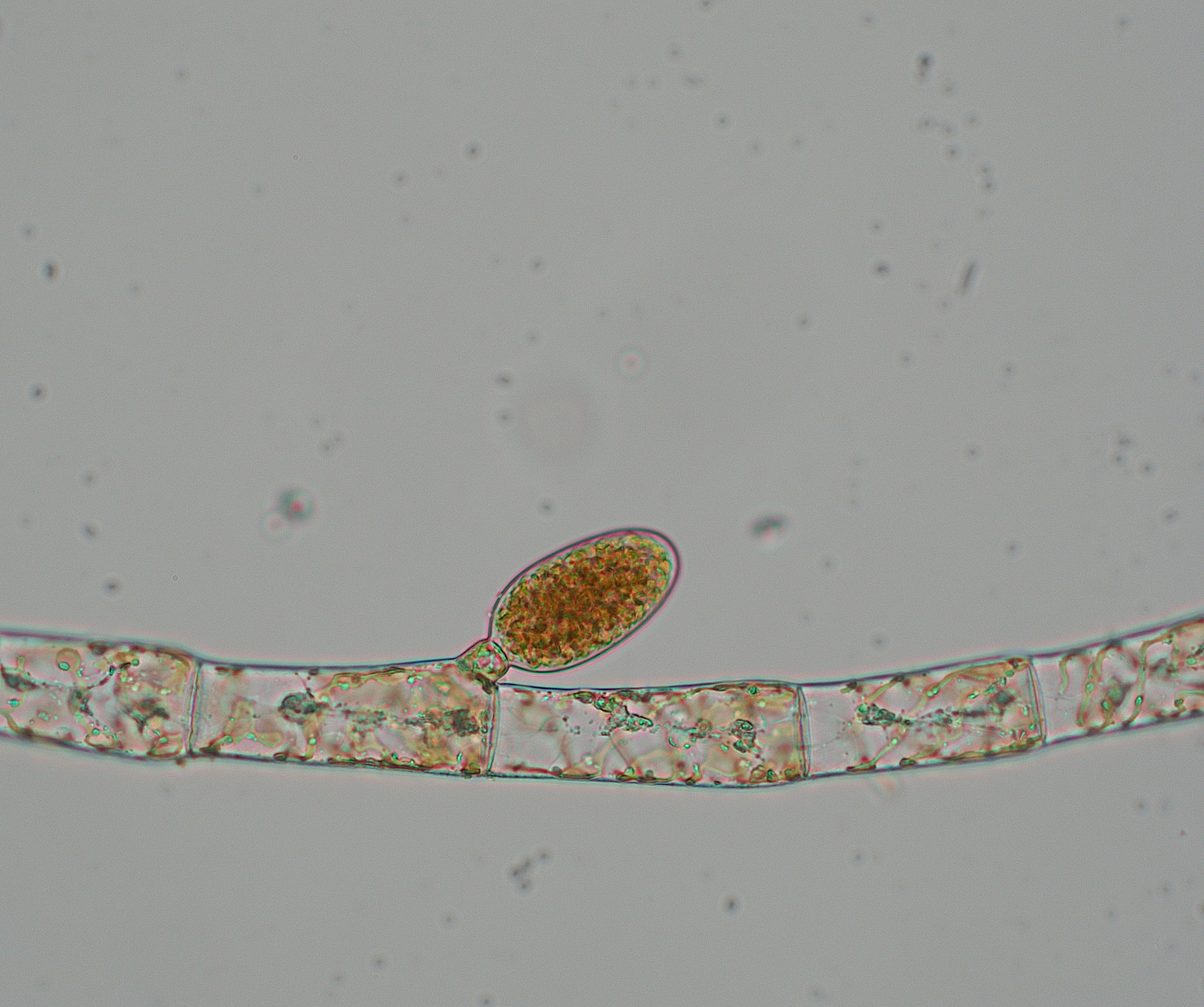

Supplement: Supplementary file 5 — Source data Fig. 1 [file 44319_2025_605_MOESM5_ESM.zip › Figure 1/1B/Ec17 14ND 10percentx40Basler_210323.tiff]

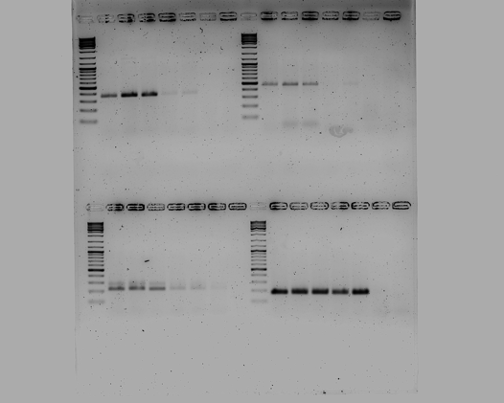

Supplement: Supplementary file 5 — Source data Fig. 1 [file 44319_2025_605_MOESM5_ESM.zip › Figure 1/1E/Fig1E_rt-PCR.png]

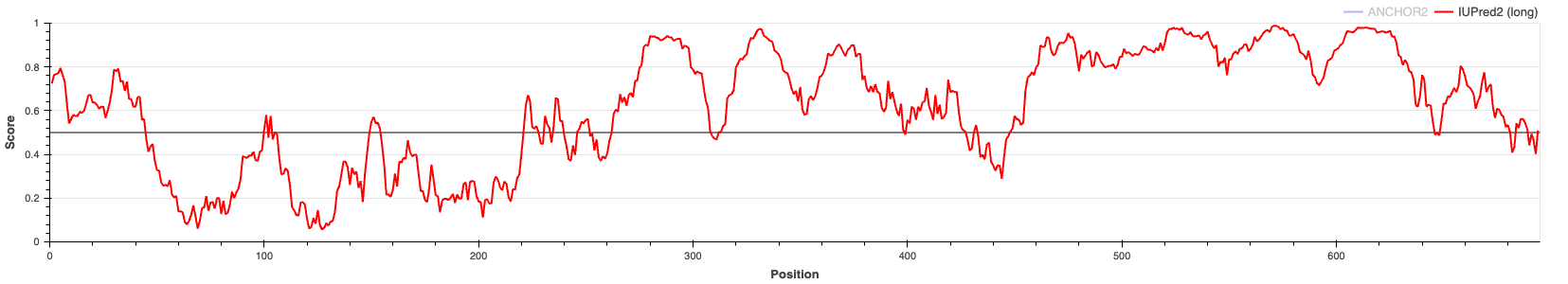

Supplement: Supplementary file 5 — Source data Fig. 1 [file 44319_2025_605_MOESM5_ESM.zip › Figure 1/1D/ecHOP1_FL_IUPred-Disordered-Regions-only_plot.png]

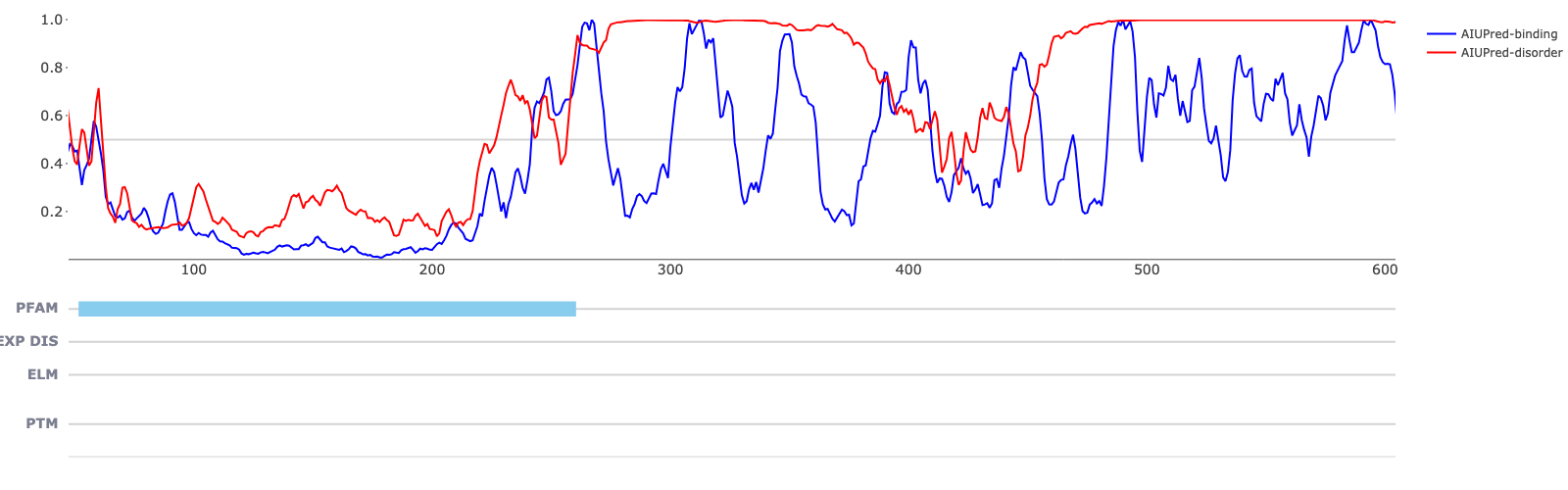

Supplement: Supplementary file 5 — Source data Fig. 1 [file 44319_2025_605_MOESM5_ESM.zip › Figure 1/1D/ecHOP1_FL_IUPred-and ANCHOR2-Plots_plot.png]

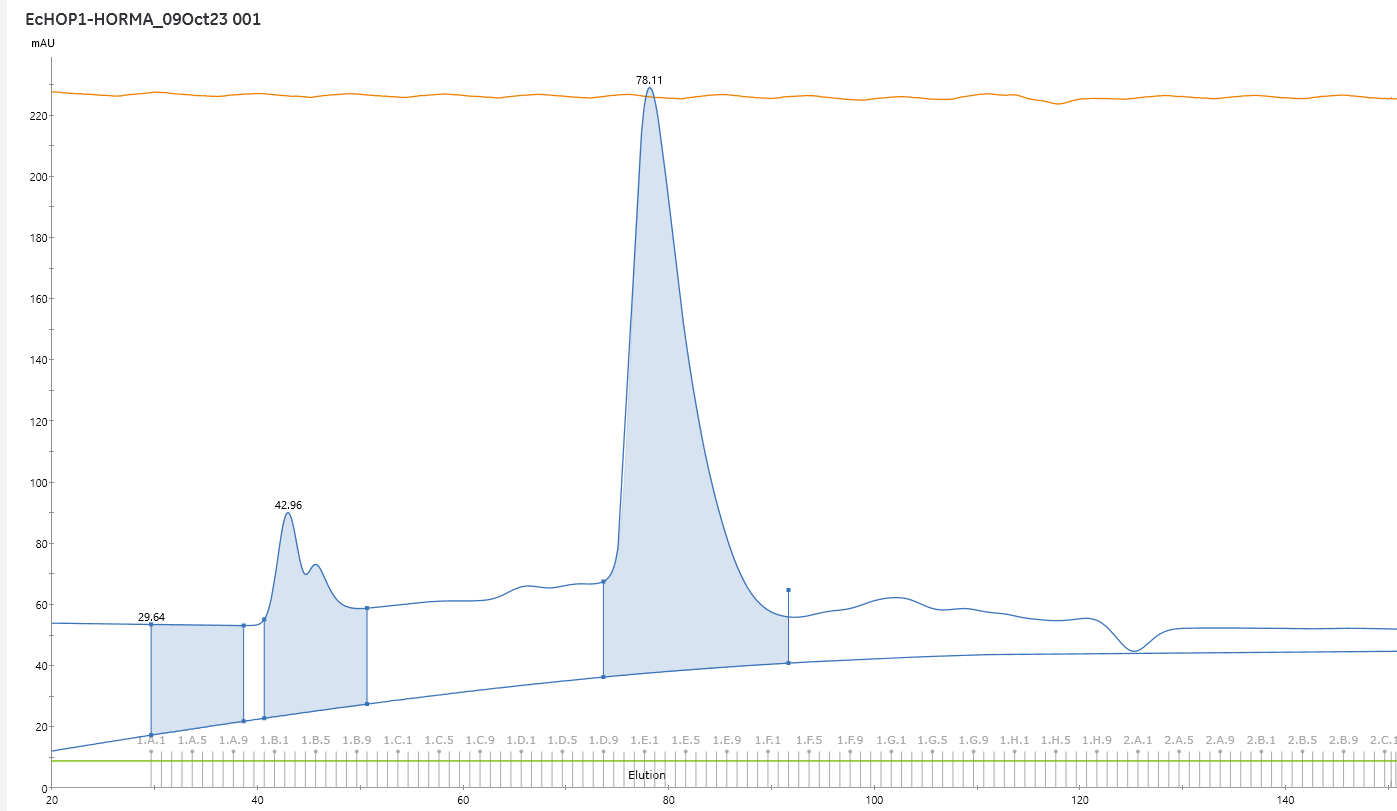

Supplement: Supplementary file 6 — Source data Fig. 2 [file 44319_2025_605_MOESM6_ESM.zip › Figure 2/2A/Figure2A_StrepII-EcHOP1-HORMA_SEC-Chromatogram.png]

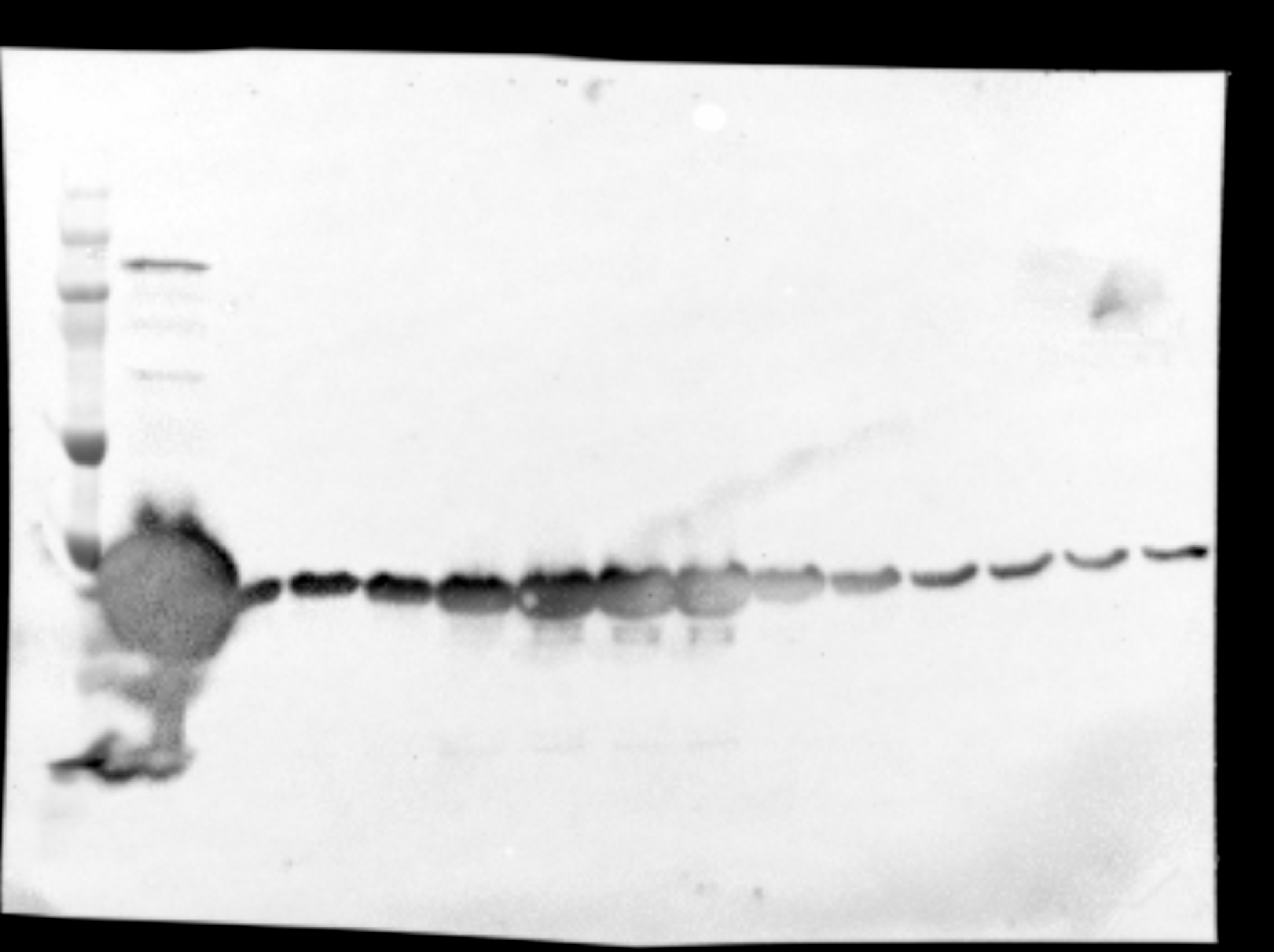

Supplement: Supplementary file 6 — Source data Fig. 2 [file 44319_2025_605_MOESM6_ESM.zip › Figure 2/2A/Figure2A_StrepII-EcHOP1-HORMA_SEC-Fractions-Merge.tif]

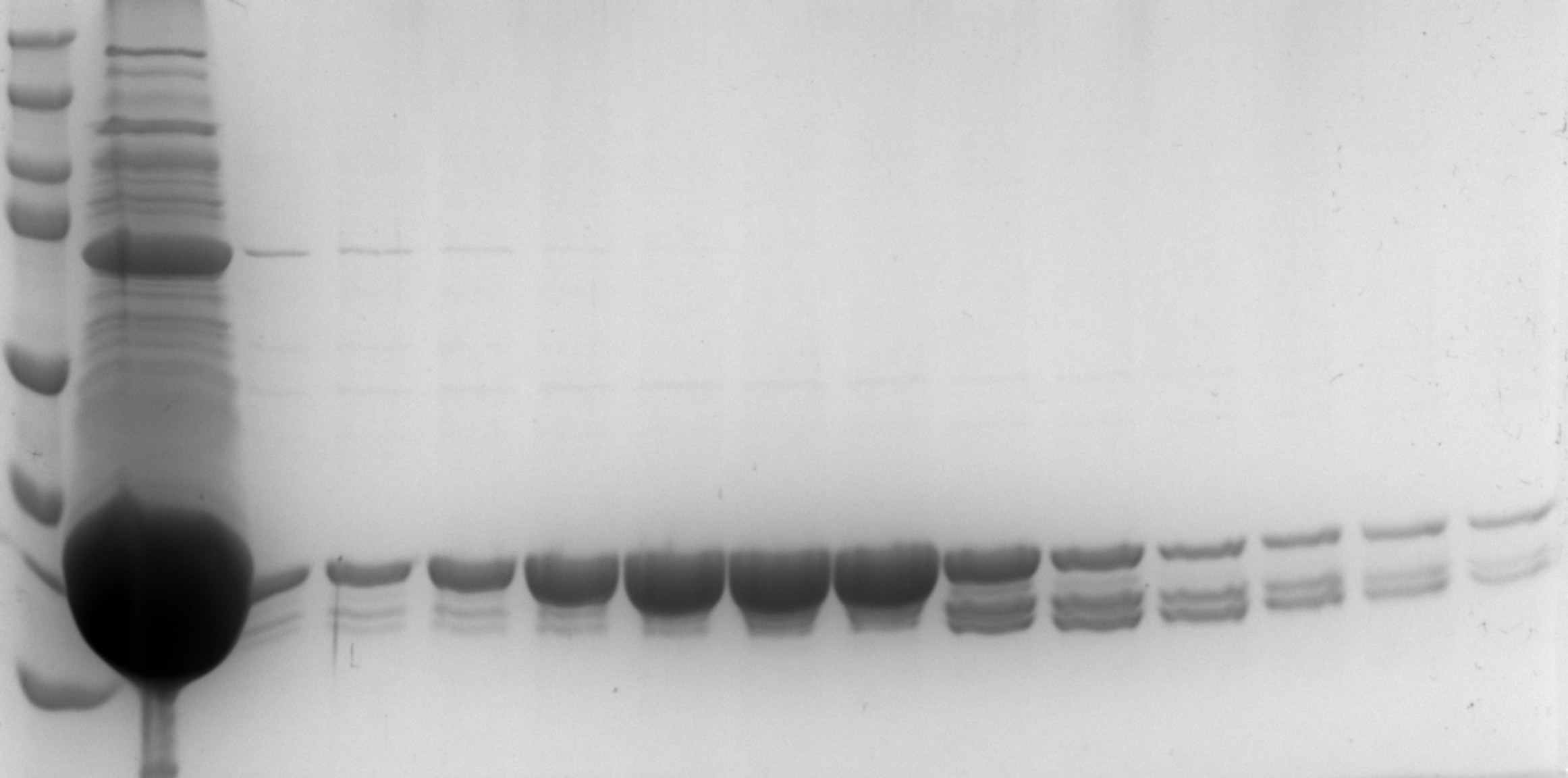

Supplement: Supplementary file 6 — Source data Fig. 2 [file 44319_2025_605_MOESM6_ESM.zip › Figure 2/2A/Figure2A_StrepII-EcHOP1-HORMA_SEC-Fractions_coomassie.tif]

# Molar\_Mass

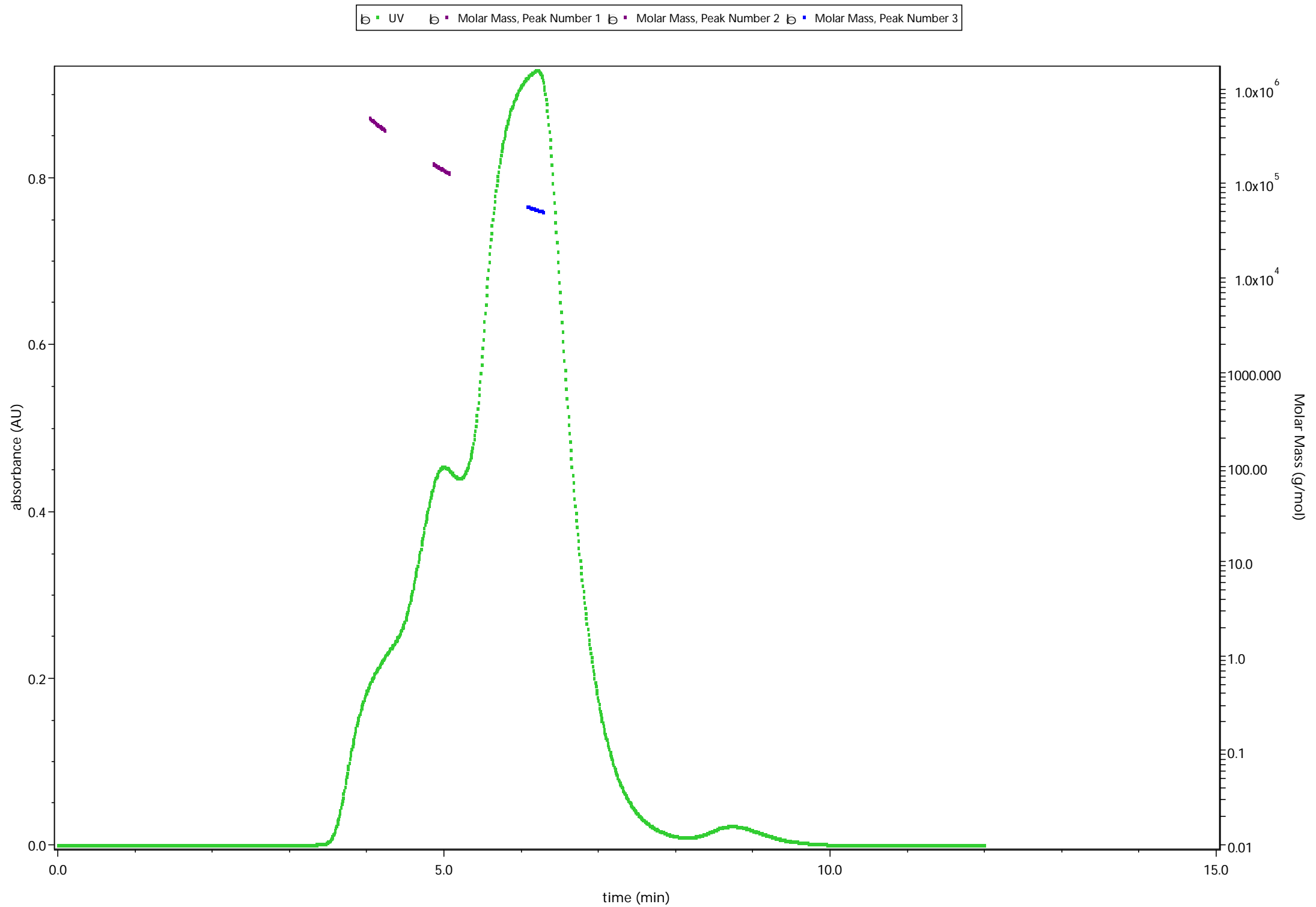

Supplement: Supplementary file 6 — Source data Fig. 2 [file 44319_2025_605_MOESM6_ESM.zip › Figure 2/2B/Graph-BSA_12Jul23.pdf]

# Molar\_Mass

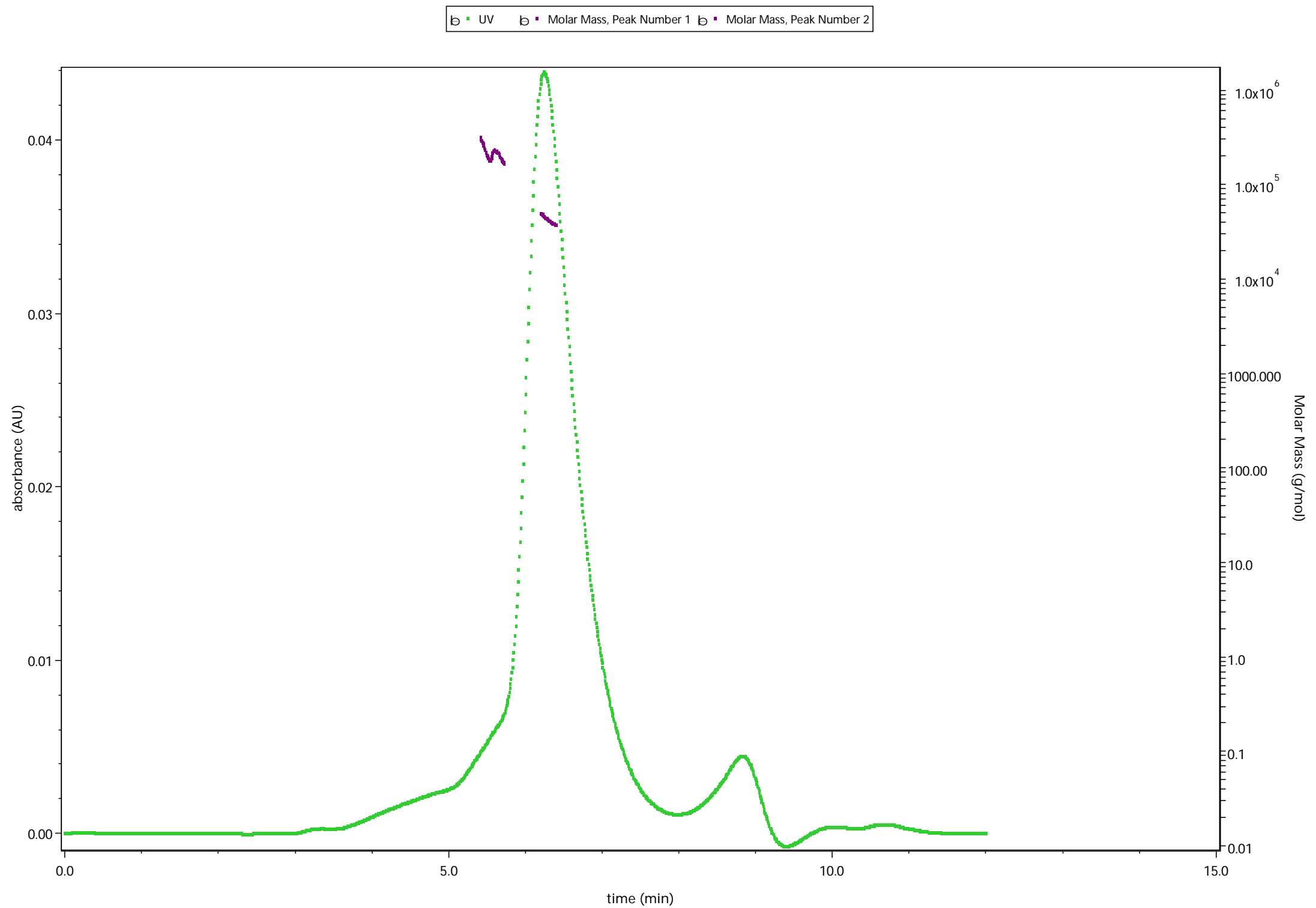

Supplement: Supplementary file 6 — Source data Fig. 2 [file 44319_2025_605_MOESM6_ESM.zip › Figure 2/2B/Graph-EcHOP1-HORMA-1_12Jul23.pdf]

Molar\_Mass

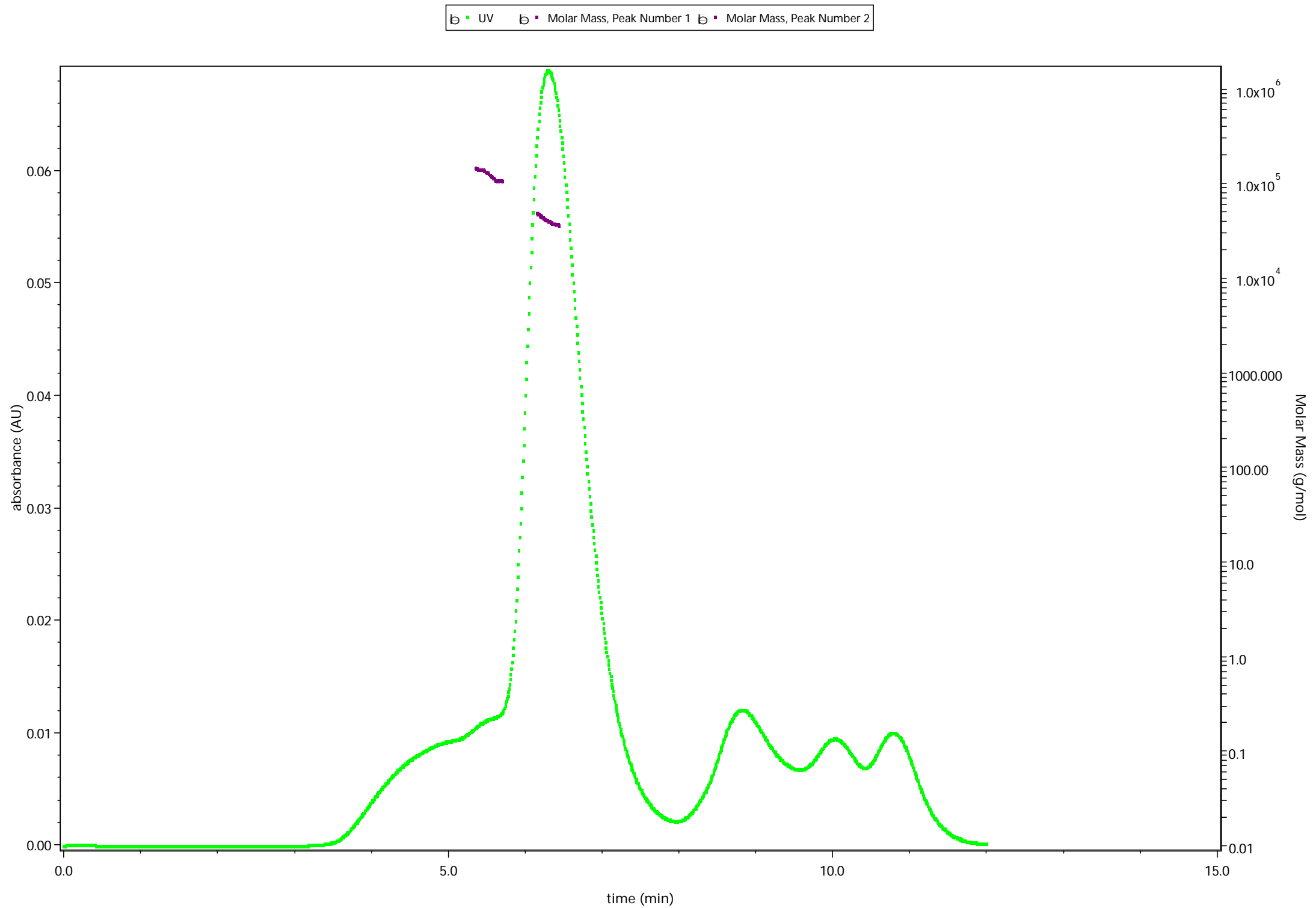

Supplement: Supplementary file 6 — Source data Fig. 2 [file 44319_2025_605_MOESM6_ESM.zip › Figure 2/2B/Graph-EcHOP1-HORMA-2_12Jul23.pdf]

Molar\_Mass

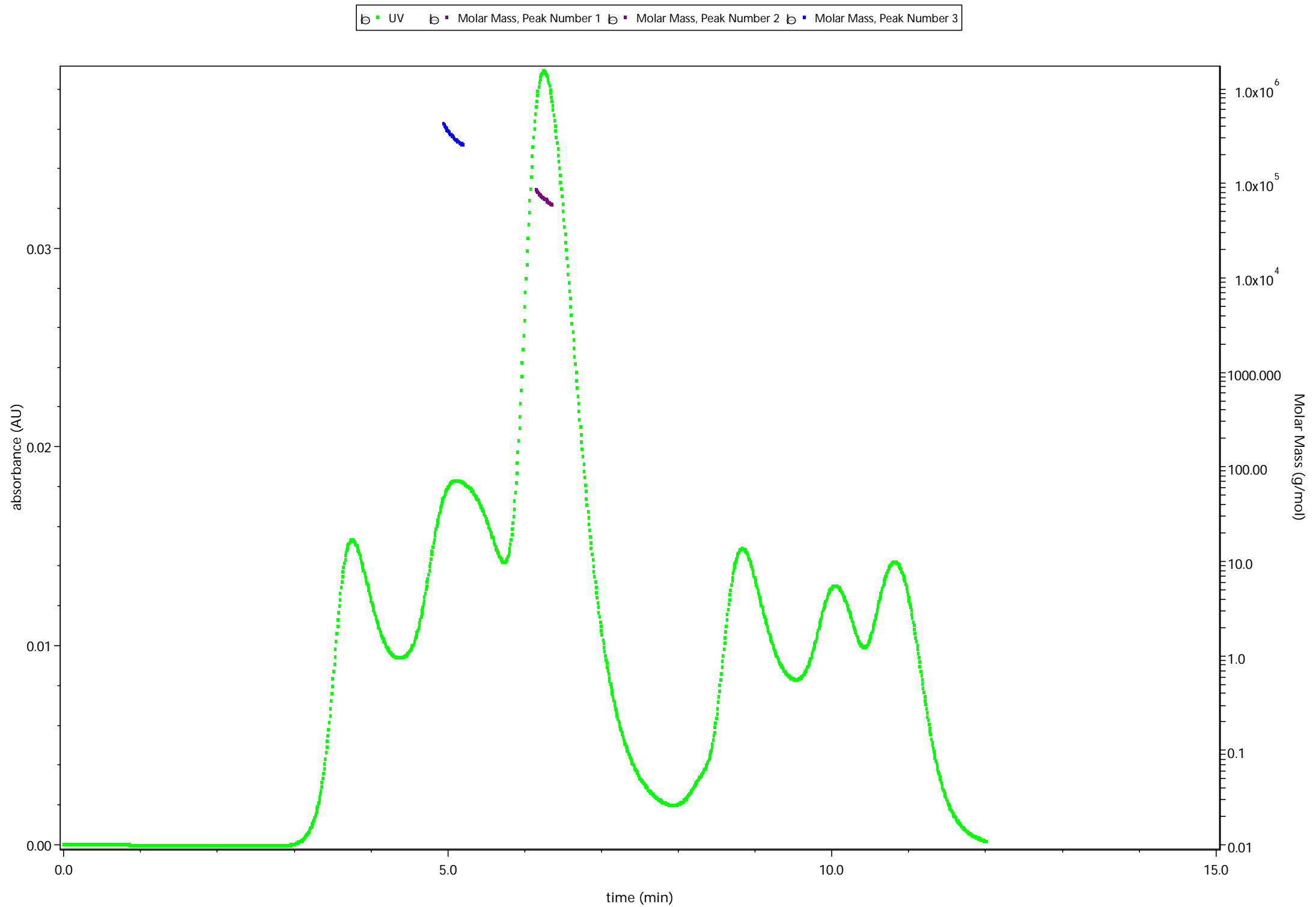

Supplement: Supplementary file 6 — Source data Fig. 2 [file 44319_2025_605_MOESM6_ESM.zip › Figure 2/2B/Graph-EcHOP1-HORMA-3_12Jul23.pdf]

# Molar\_Mass

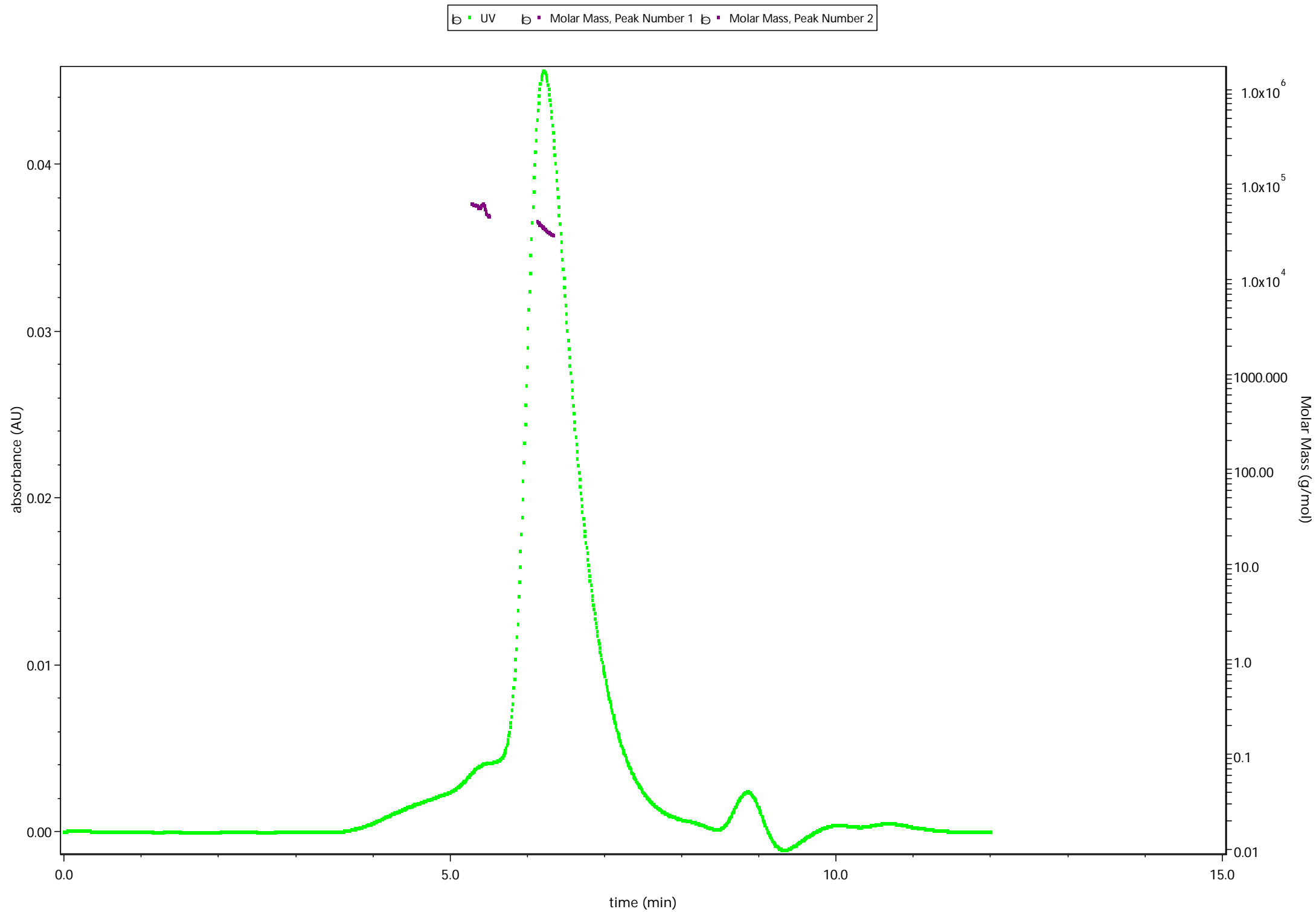

Supplement: Supplementary file 6 — Source data Fig. 2 [file 44319_2025_605_MOESM6_ESM.zip › Figure 2/2B/Graph-EcHOP1-HORMA-4_12Jul23.pdf]

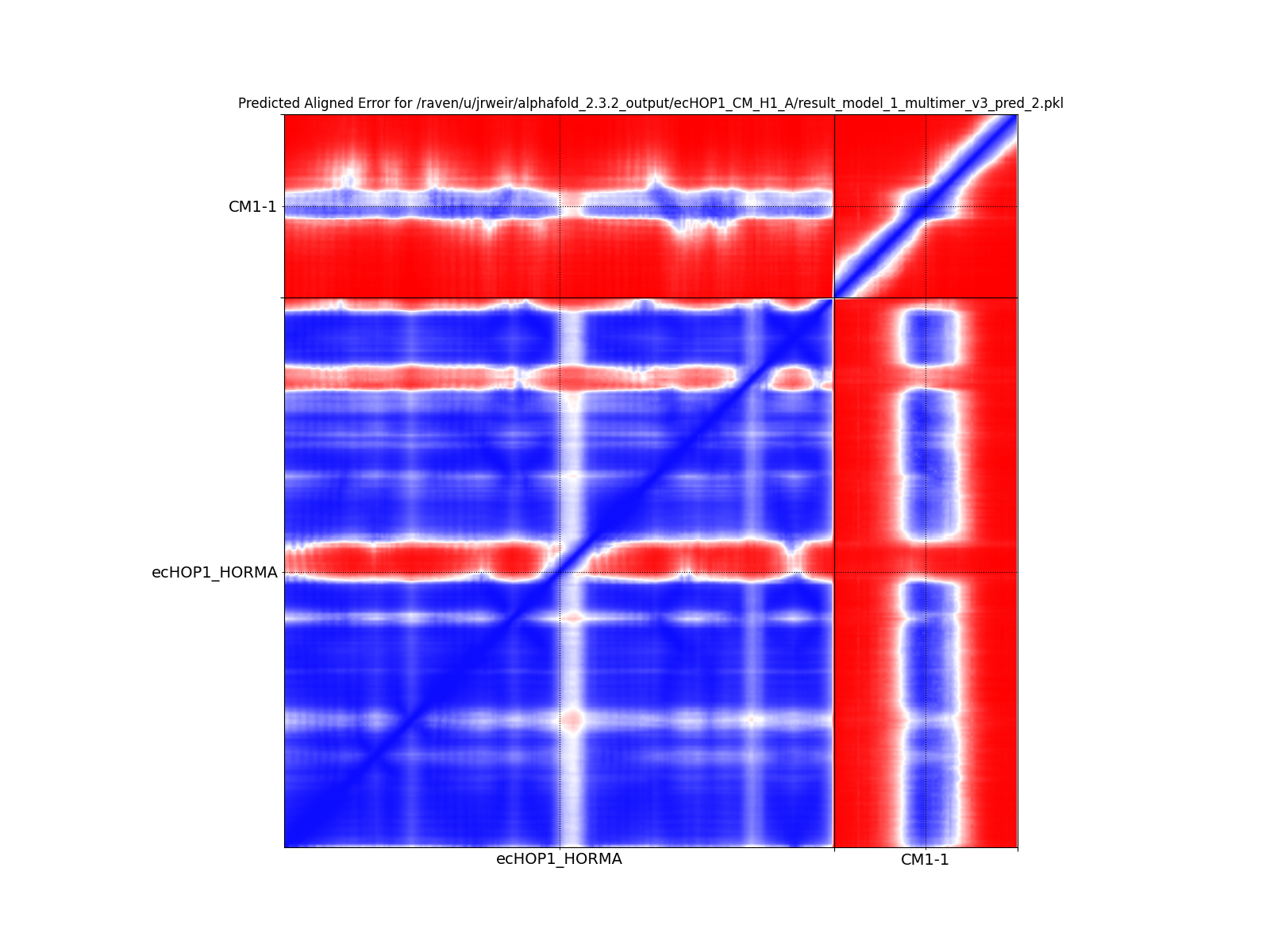

Supplement: Supplementary file 6 — Source data Fig. 2 [file 44319_2025_605_MOESM6_ESM.zip › Figure 2/2C/pae_ranked_0.png]

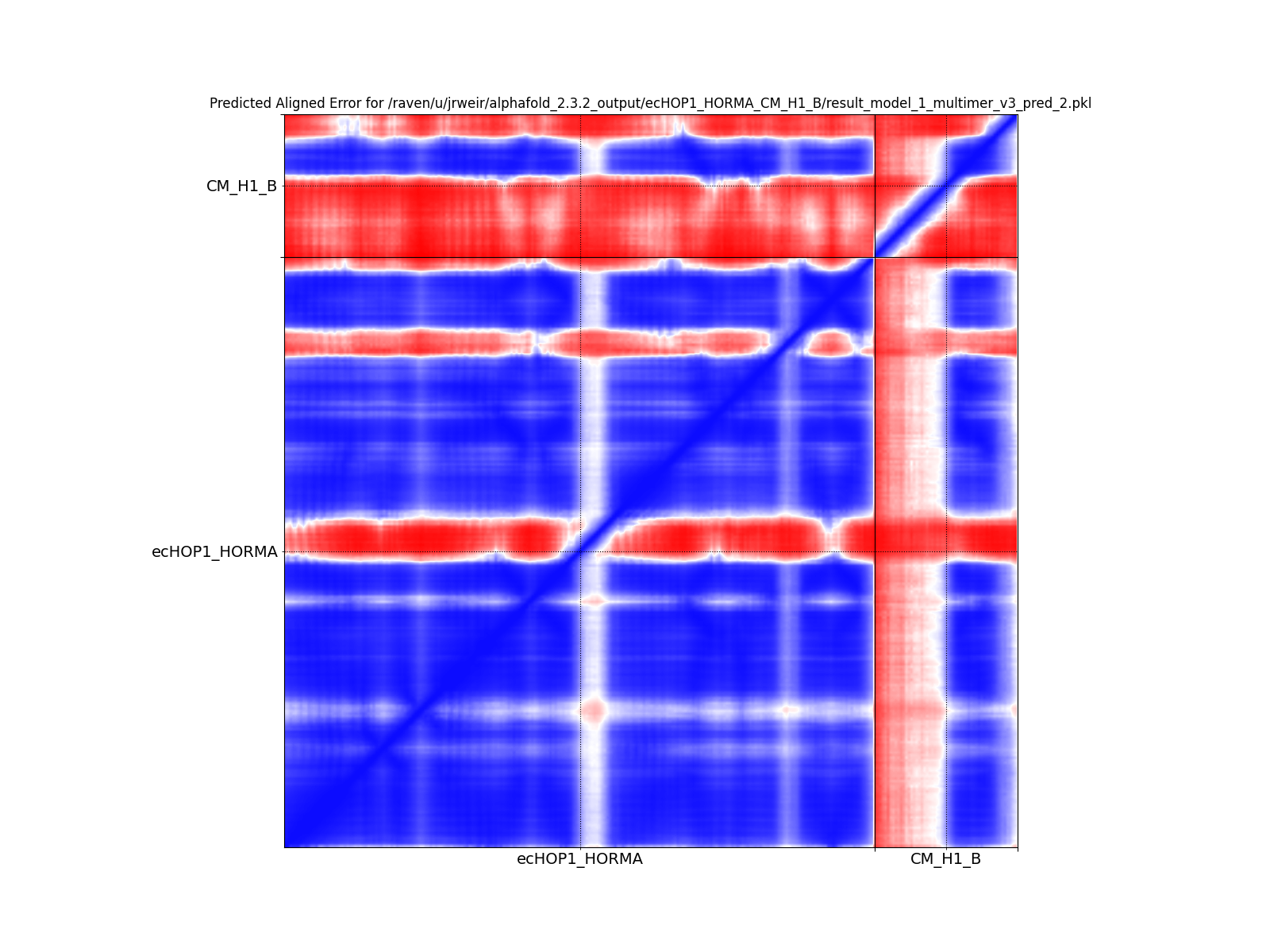

Supplement: Supplementary file 6 — Source data Fig. 2 [file 44319_2025_605_MOESM6_ESM.zip › Figure 2/2D/pae_ranked_0.png]

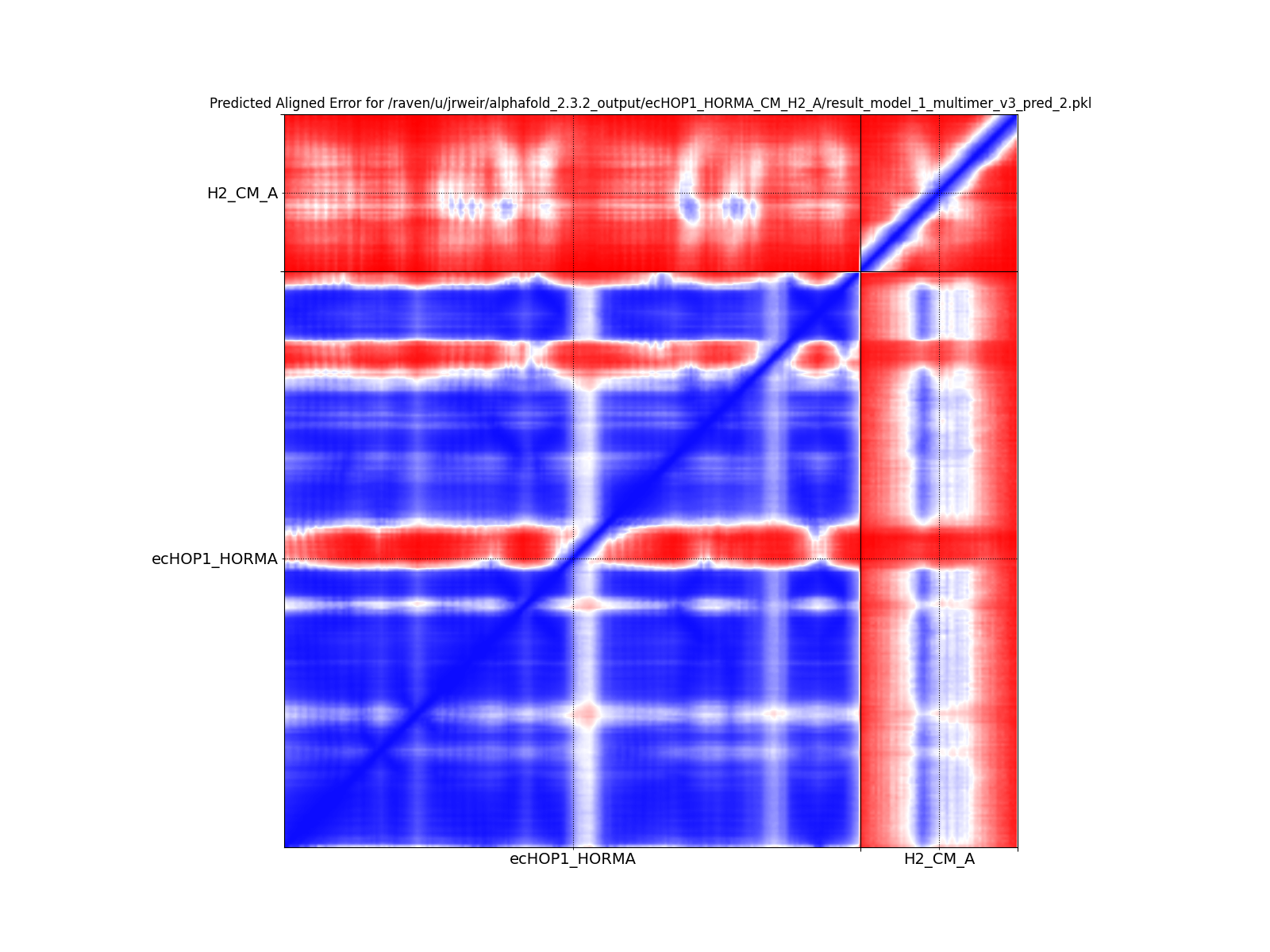

Supplement: Supplementary file 6 — Source data Fig. 2 [file 44319_2025_605_MOESM6_ESM.zip › Figure 2/2E/pae_ranked_0.png]

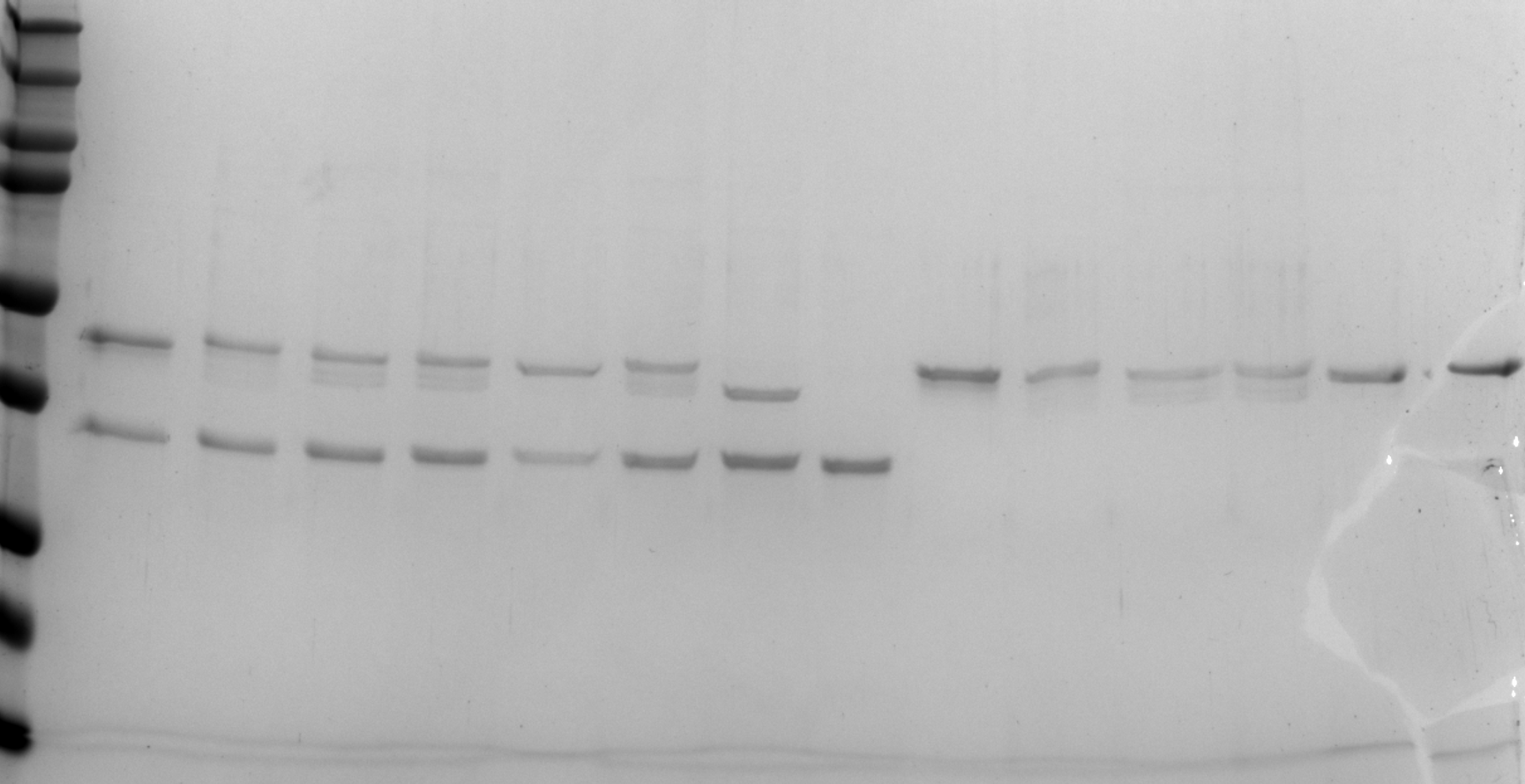

Supplement: Supplementary file 6 — Source data Fig. 2 [file 44319_2025_605_MOESM6_ESM.zip › Figure 2/2G/Fig2G_StrepII-ecHOP1-HORMA_CMs-all_Coomassie_Pull-Down_Gel1-Input.tif]

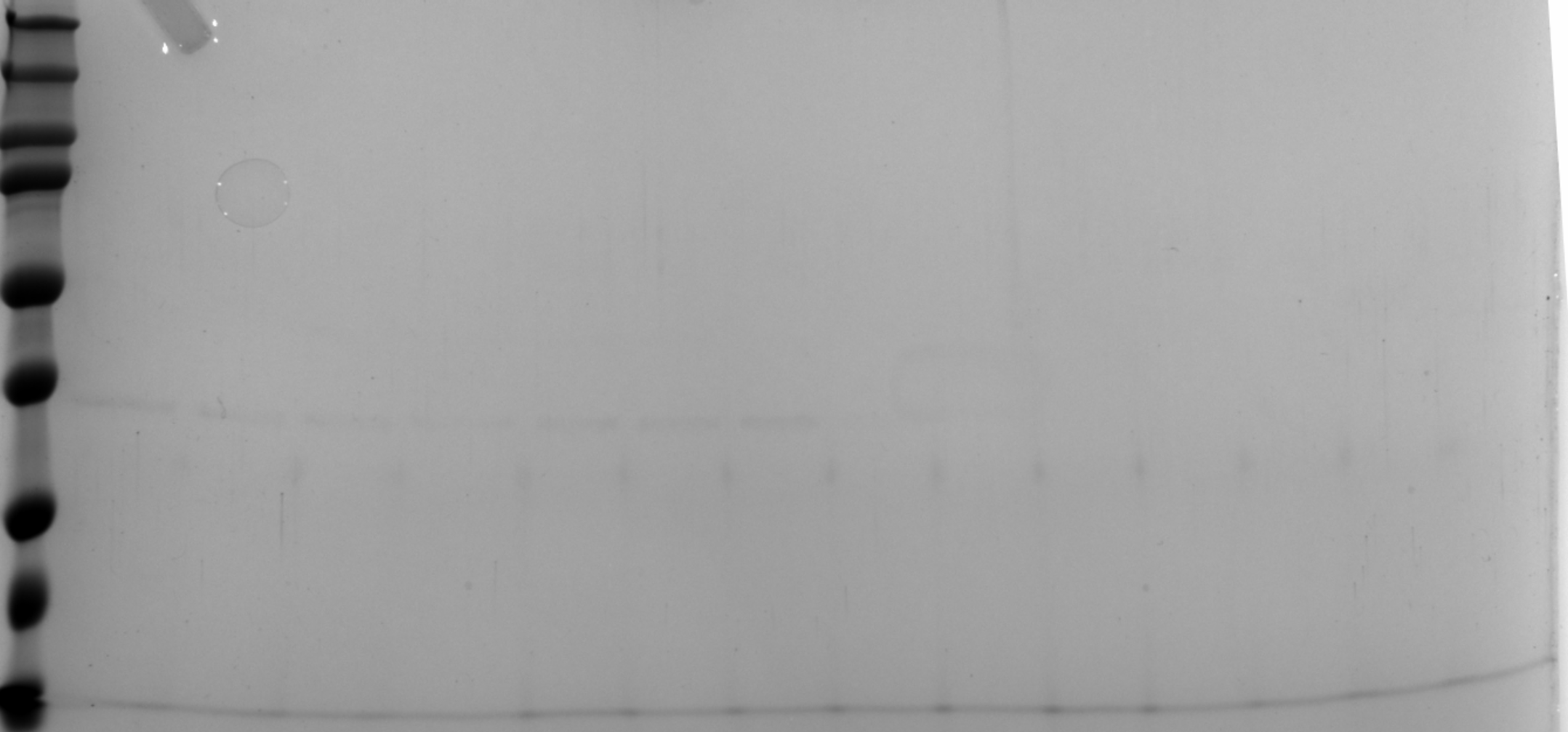

Supplement: Supplementary file 6 — Source data Fig. 2 [file 44319_2025_605_MOESM6_ESM.zip › Figure 2/2G/Fig2G_StrepII-ecHOP1-HORMA_CMs-all_Coomassie_Pull-Down_Gel4-Elute.tif]

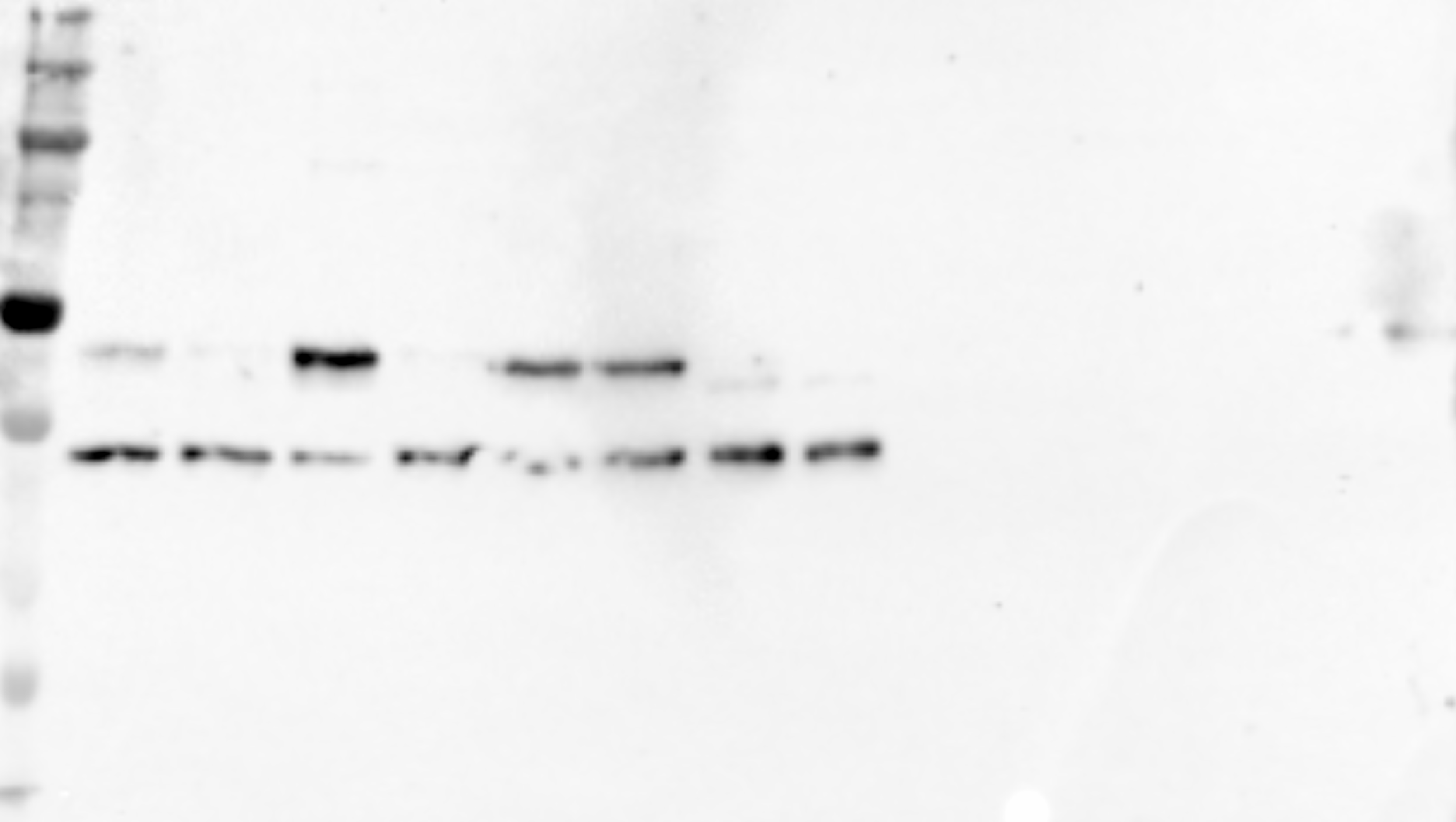

Supplement: Supplementary file 6 — Source data Fig. 2 [file 44319_2025_605_MOESM6_ESM.zip › Figure 2/2G/Fig2G_StrepII-ecHOP1-HORMA_CMs-all_Pull-Down_a-Strep_a-MBP_Gel4-Elute-Merge.tif]

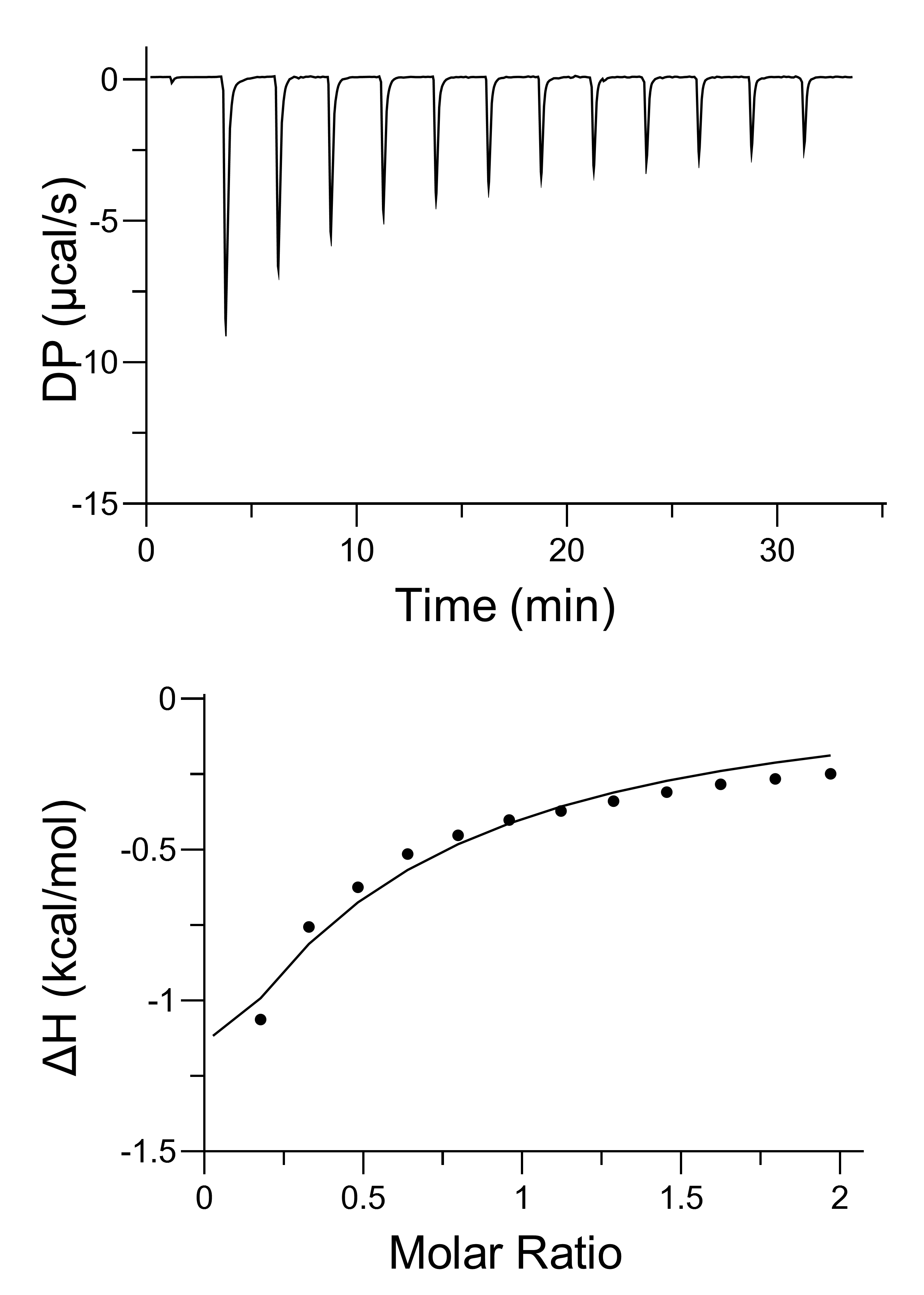

Supplement: Supplementary file 6 — Source data Fig. 2 [file 44319_2025_605_MOESM6_ESM.zip › Figure 2/2H-J/Figure2H_HORMA-25uM_CM-H1-A-350uM_Graph.bmp]

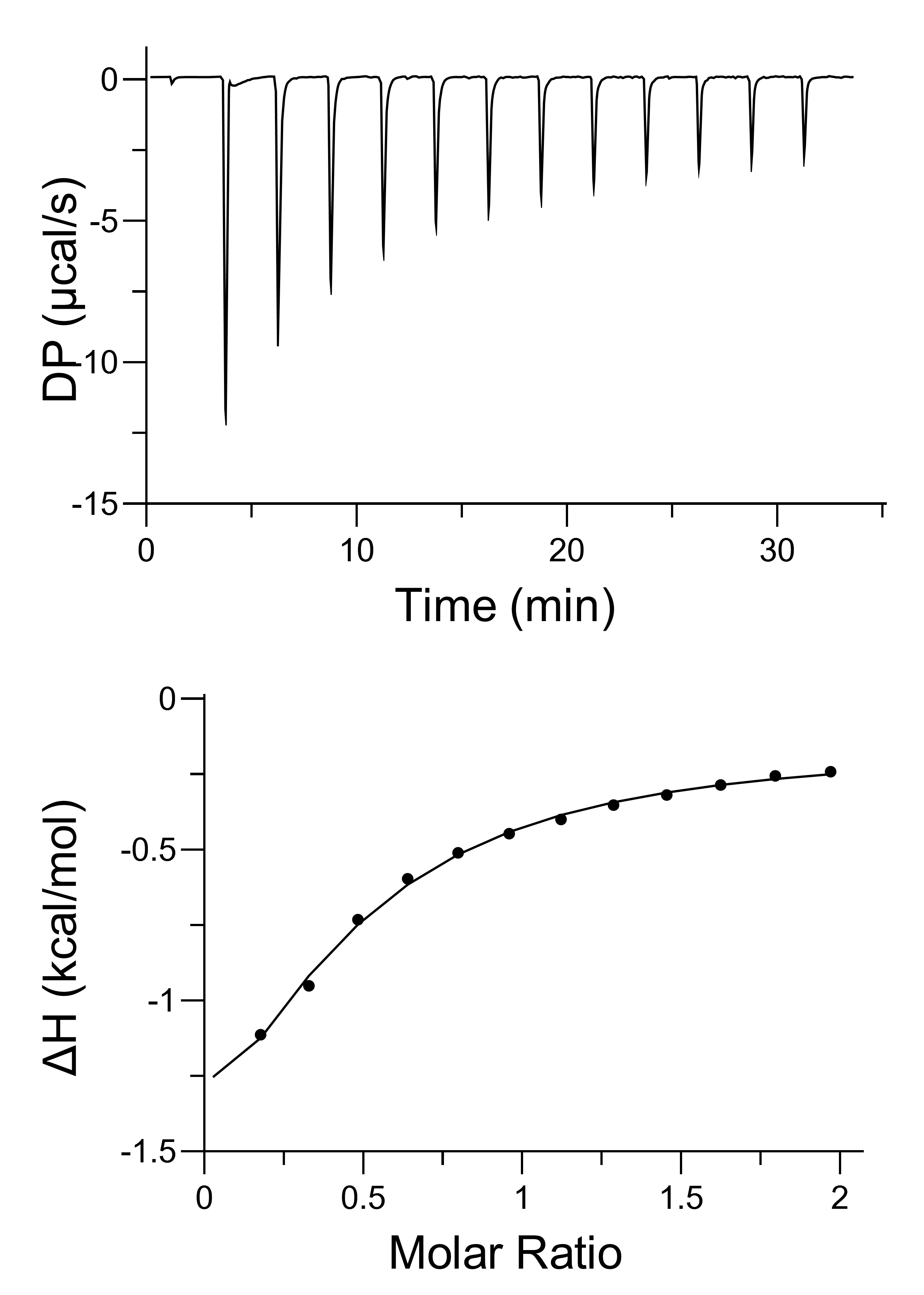

Supplement: Supplementary file 6 — Source data Fig. 2 [file 44319_2025_605_MOESM6_ESM.zip › Figure 2/2H-J/Figure2H_HORMA-25uM_CM-H1-B-350uM_Graph.bmp]

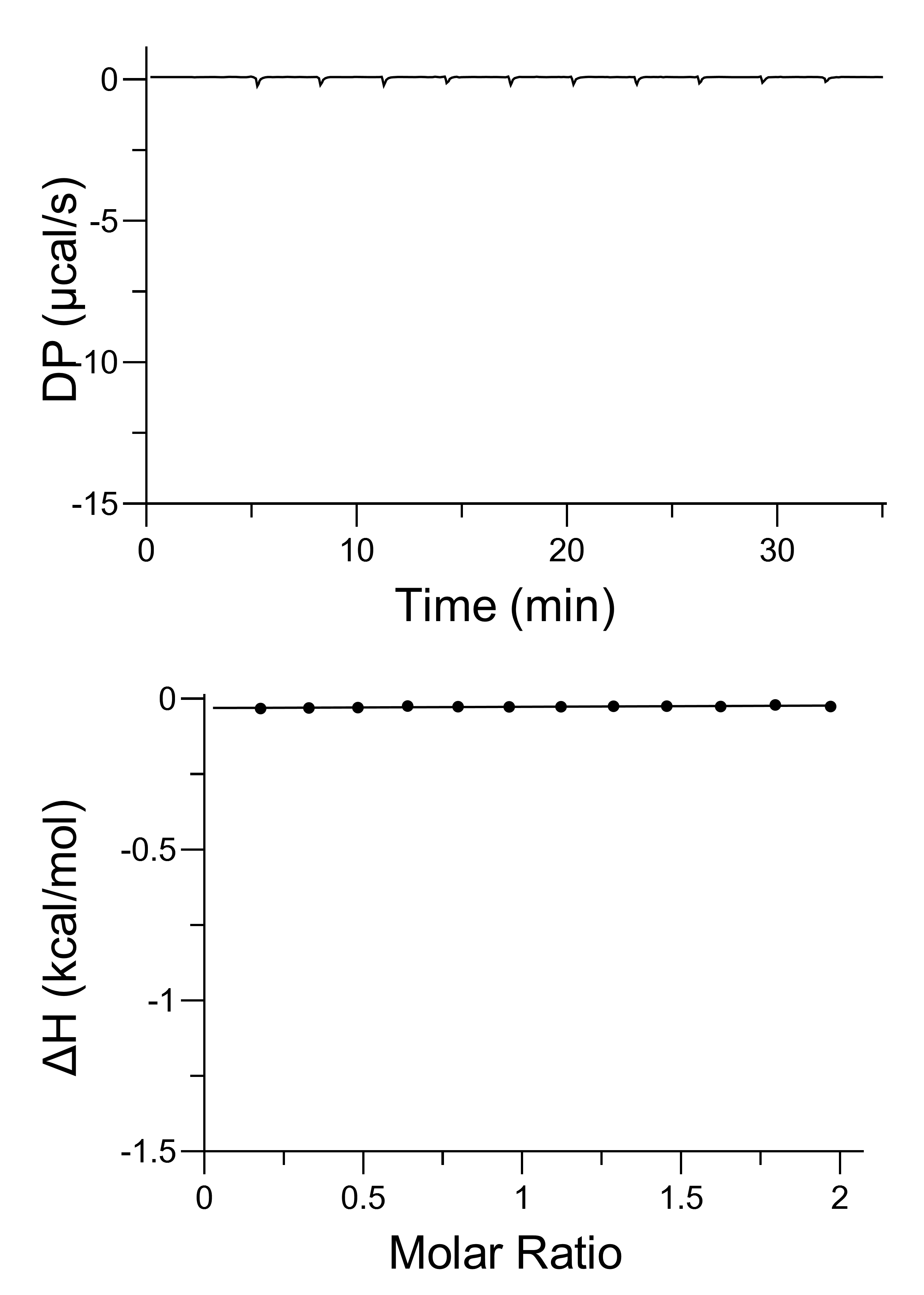

Supplement: Supplementary file 6 — Source data Fig. 2 [file 44319_2025_605_MOESM6_ESM.zip › Figure 2/2H-J/Figure2H_HORMA-25uM_CM-H2-A-350uM_Graph.bmp]

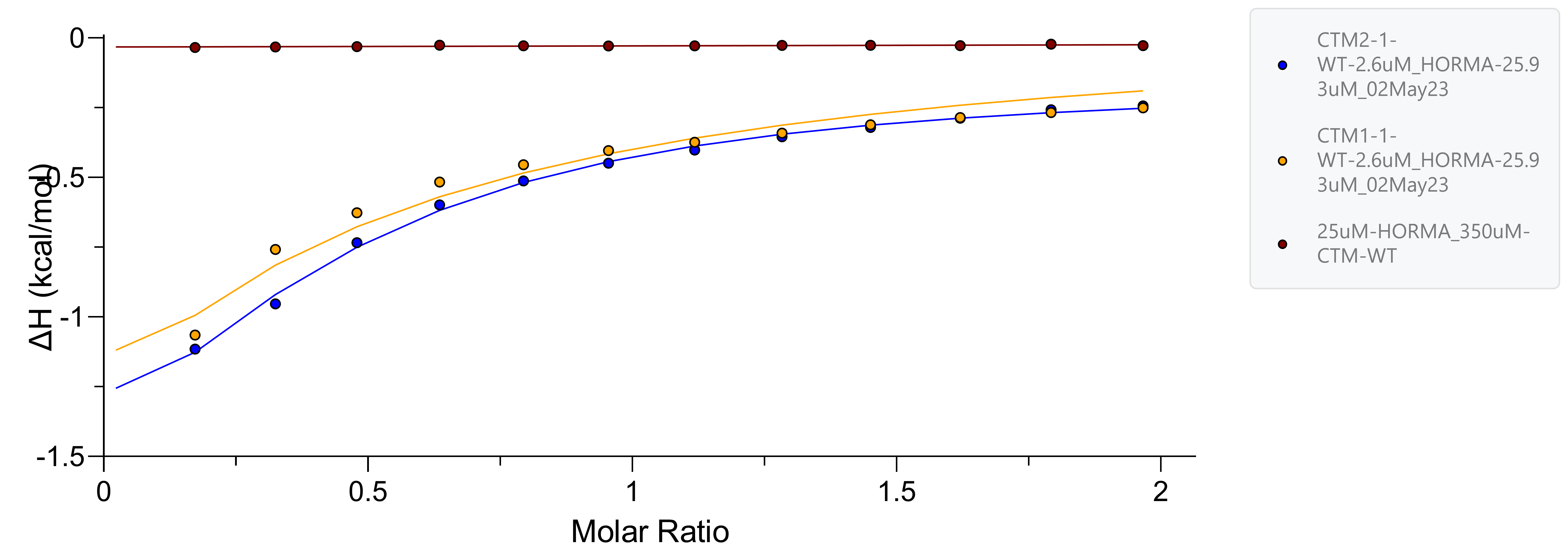

Supplement: Supplementary file 6 — Source data Fig. 2 [file 44319_2025_605_MOESM6_ESM.zip › Figure 2/2H-J/Figure2H_Merged-Data_All-CMs_Normalized-Inj-Heat.bmp]

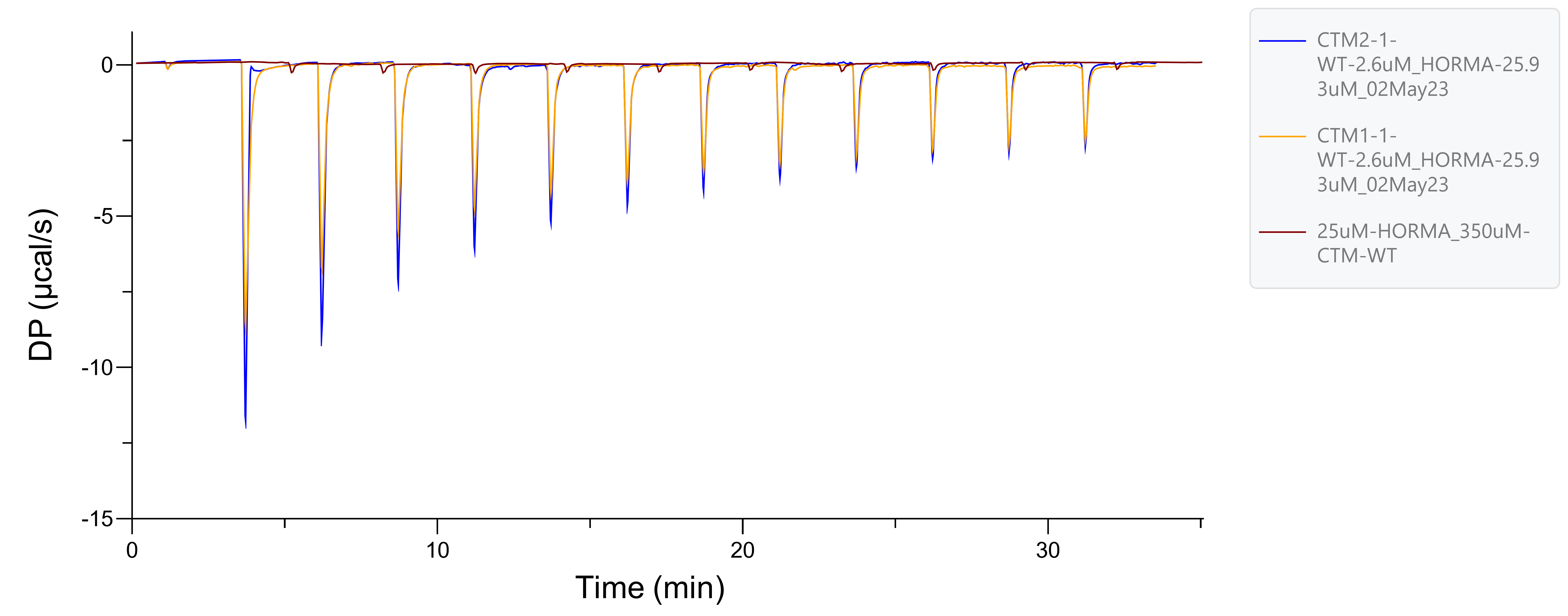

Supplement: Supplementary file 6 — Source data Fig. 2 [file 44319_2025_605_MOESM6_ESM.zip › Figure 2/2H-J/Figure2H_Merged-Data_All-CMs_Raw-Heats.bmp]

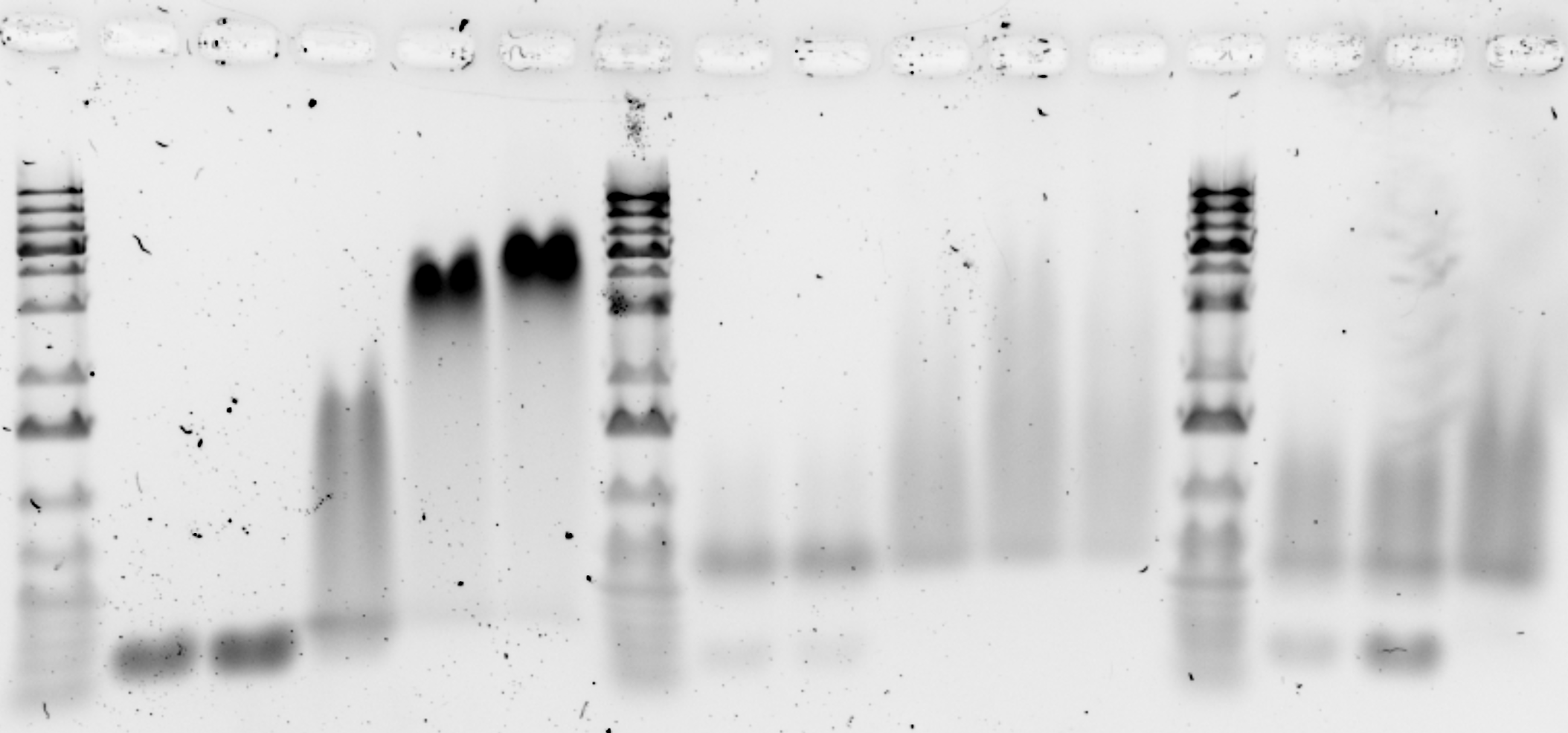

Supplement: Supplementary file 7 — Source data Fig. 3 [file 44319_2025_605_MOESM7_ESM.zip › Figure 3/3A/Figure3C_wHTH-WT_v_167bp_MN-wt_MN-dualmethyl.tif]

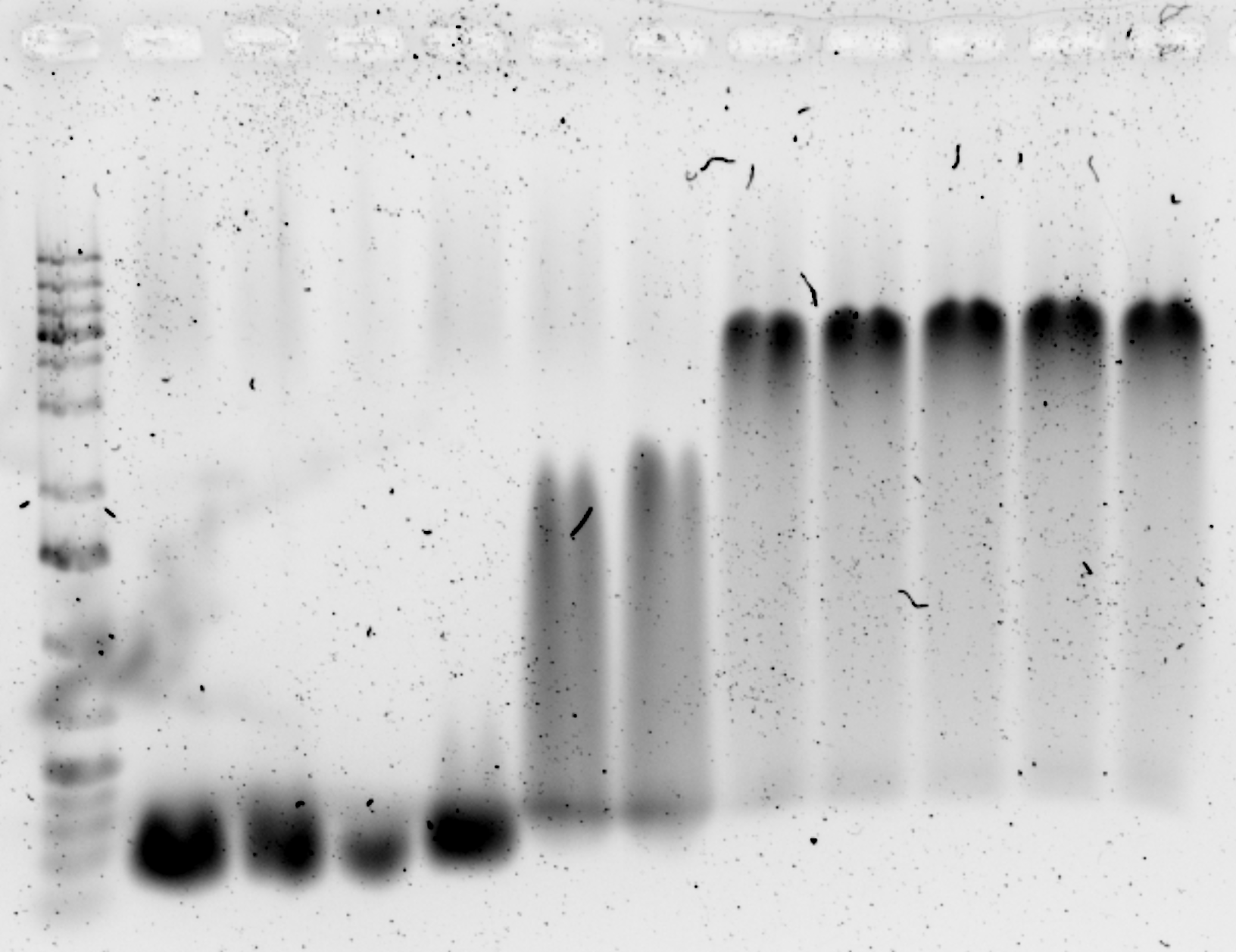

Supplement: Supplementary file 7 — Source data Fig. 3 [file 44319_2025_605_MOESM7_ESM.zip › Figure 3/3B/Figure3B_SUMO-wHTH-WT_167bp-WIDOM_EMSA_Test1.tif]

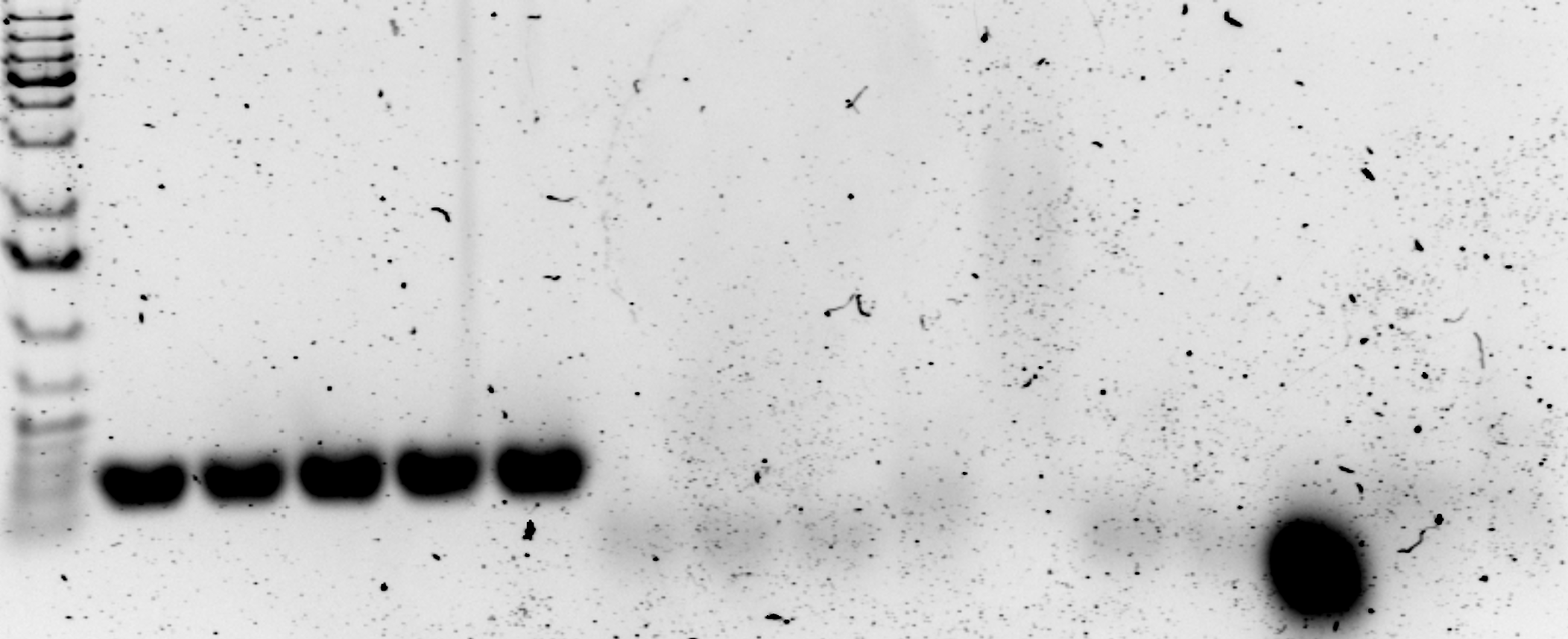

Supplement: Supplementary file 7 — Source data Fig. 3 [file 44319_2025_605_MOESM7_ESM.zip › Figure 3/3D/Figure3D_HORMA-fusion-tag_167bp-WIDOM_EMSA.tif]

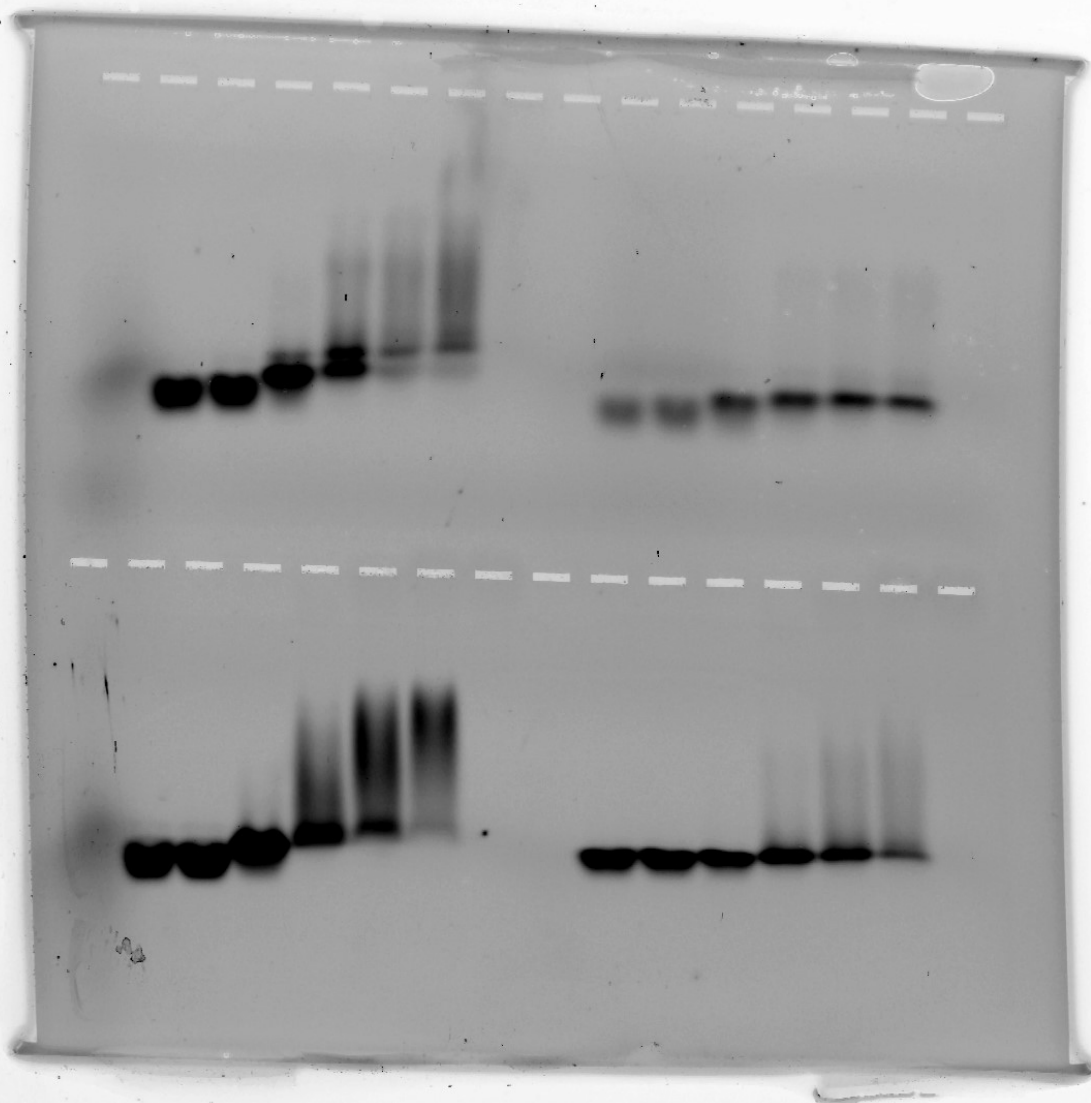

Supplement: Supplementary file 7 — Source data Fig. 3 [file 44319_2025_605_MOESM7_ESM.zip › Figure 3/3F/Figure3G_SUMO-wHTH_EMSA_ds-ssDNA_50nt-and-90nt.pdf]

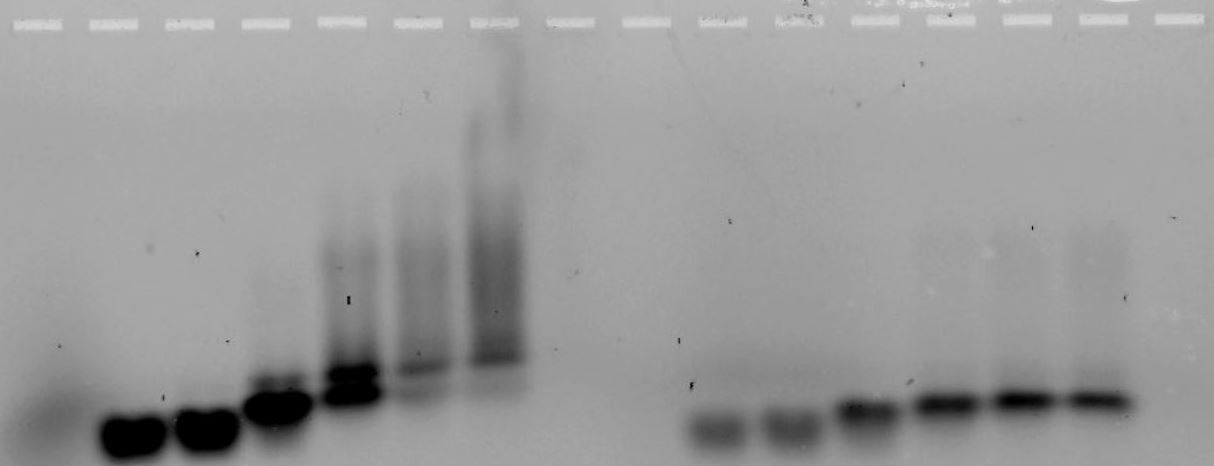

Supplement: Supplementary file 7 — Source data Fig. 3 [file 44319_2025_605_MOESM7_ESM.zip › Figure 3/3F/Figure3G_SUMO-wHTH_EMSA_ds-ssDNA_50nt.JPG]

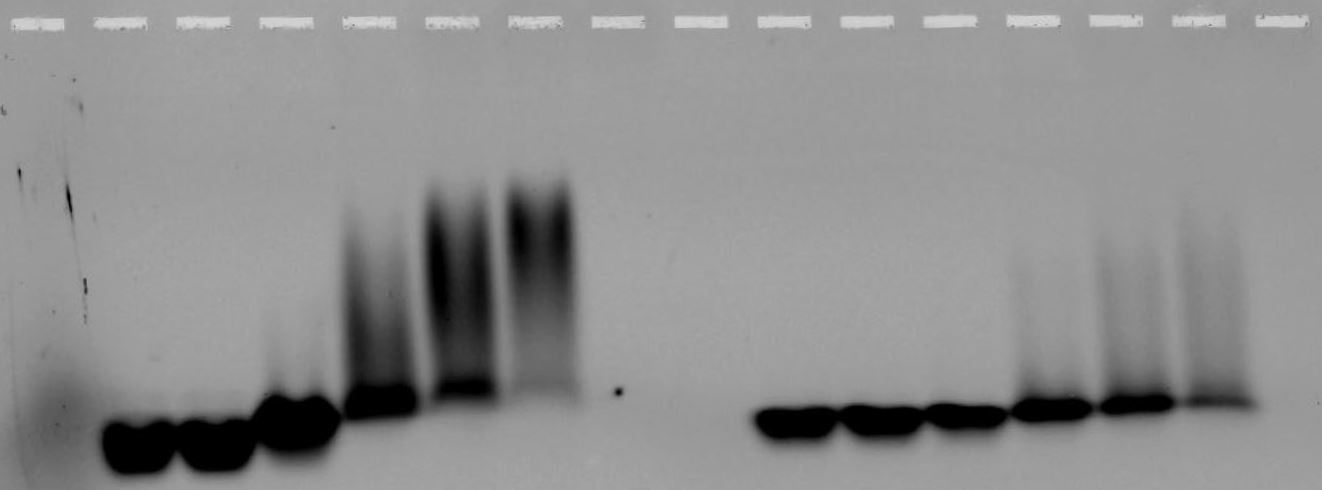

Supplement: Supplementary file 7 — Source data Fig. 3 [file 44319_2025_605_MOESM7_ESM.zip › Figure 3/3F/Figure3G_SUMO-wHTH_EMSA_ds-ssDNA_90nt.JPG]

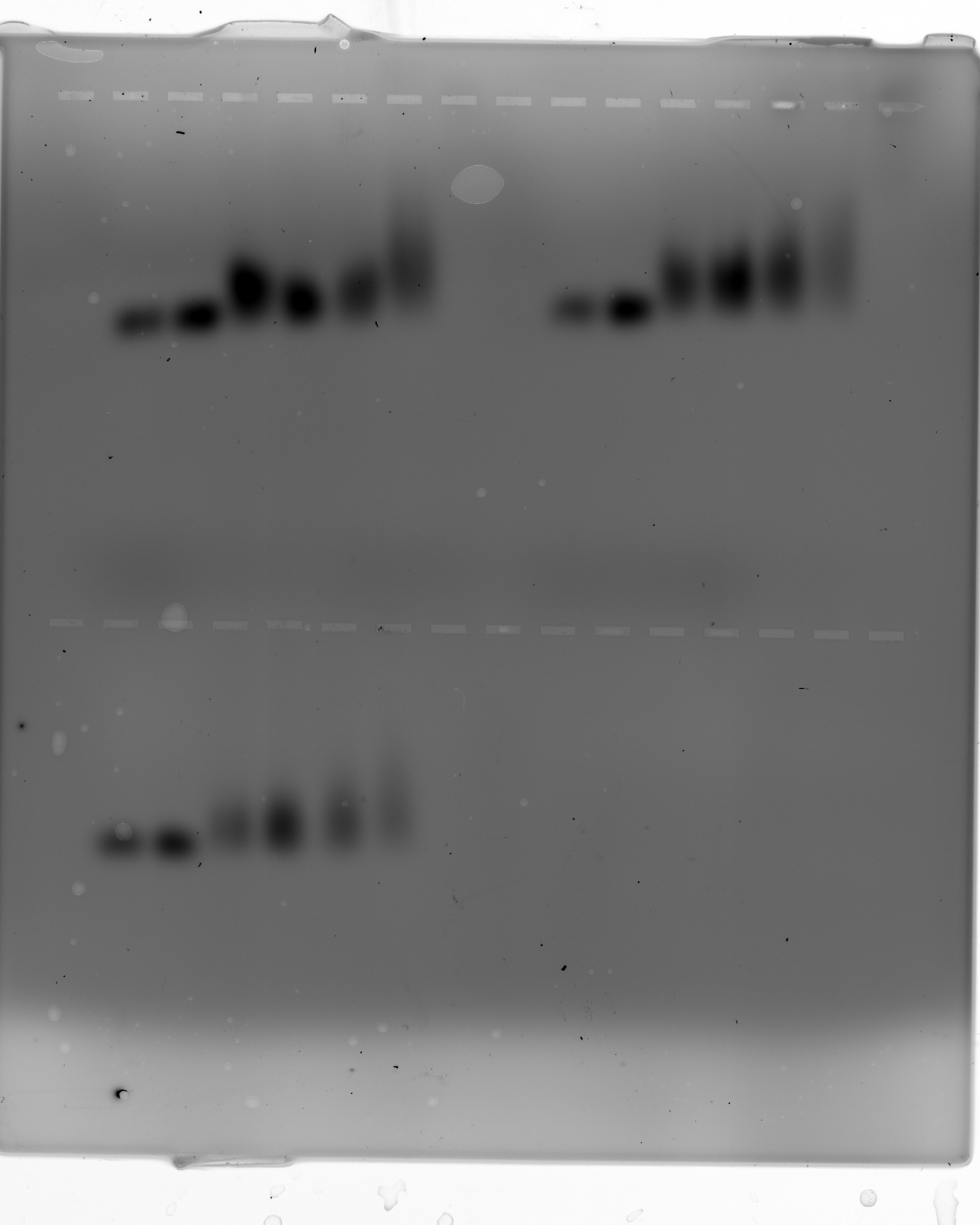

Supplement: Supplementary file 7 — Source data Fig. 3 [file 44319_2025_605_MOESM7_ESM.zip › Figure 3/3G/20250512-141350-[Cy2].gel]

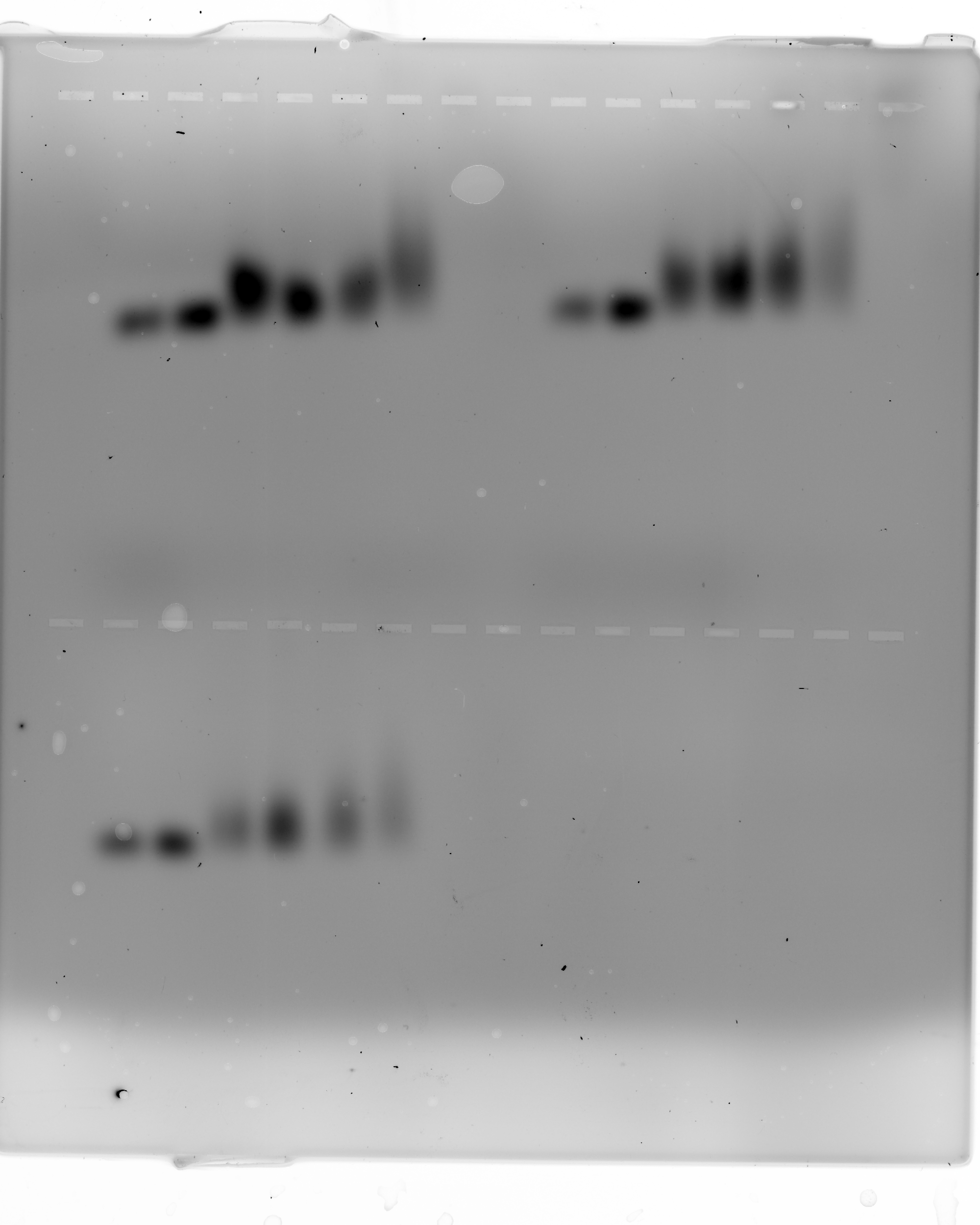

Supplement: Supplementary file 7 — Source data Fig. 3 [file 44319_2025_605_MOESM7_ESM.zip › Figure 3/3G/20250512-141350-[Cy2].tif]

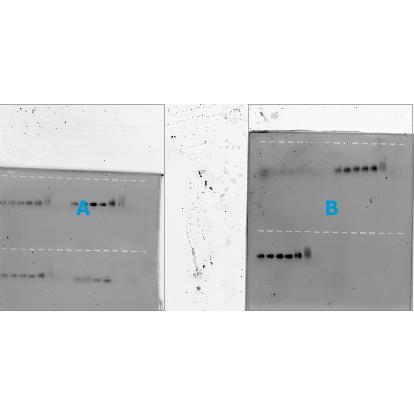

Supplement: Supplementary file 7 — Source data Fig. 3 [file 44319_2025_605_MOESM7_ESM.zip › Figure 3/3G/ecHOP1-wHTH-WT_DNA-shapes_09Apr25.jpg]

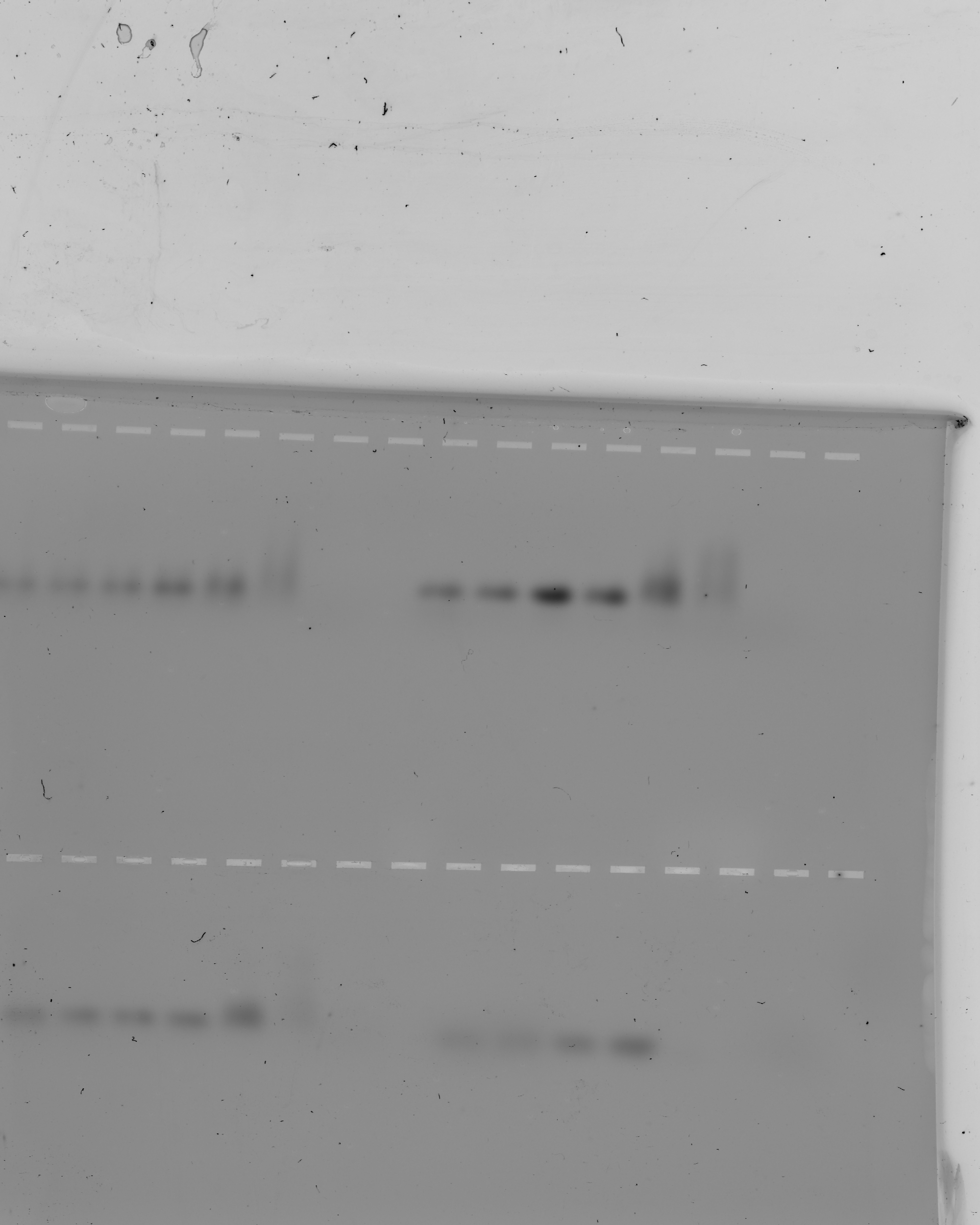

Supplement: Supplementary file 7 — Source data Fig. 3 [file 44319_2025_605_MOESM7_ESM.zip › Figure 3/3G/ecHOP1-wHTH-WT_DNA-substrates-various_09Apr25-[Cy2]-A.gel]

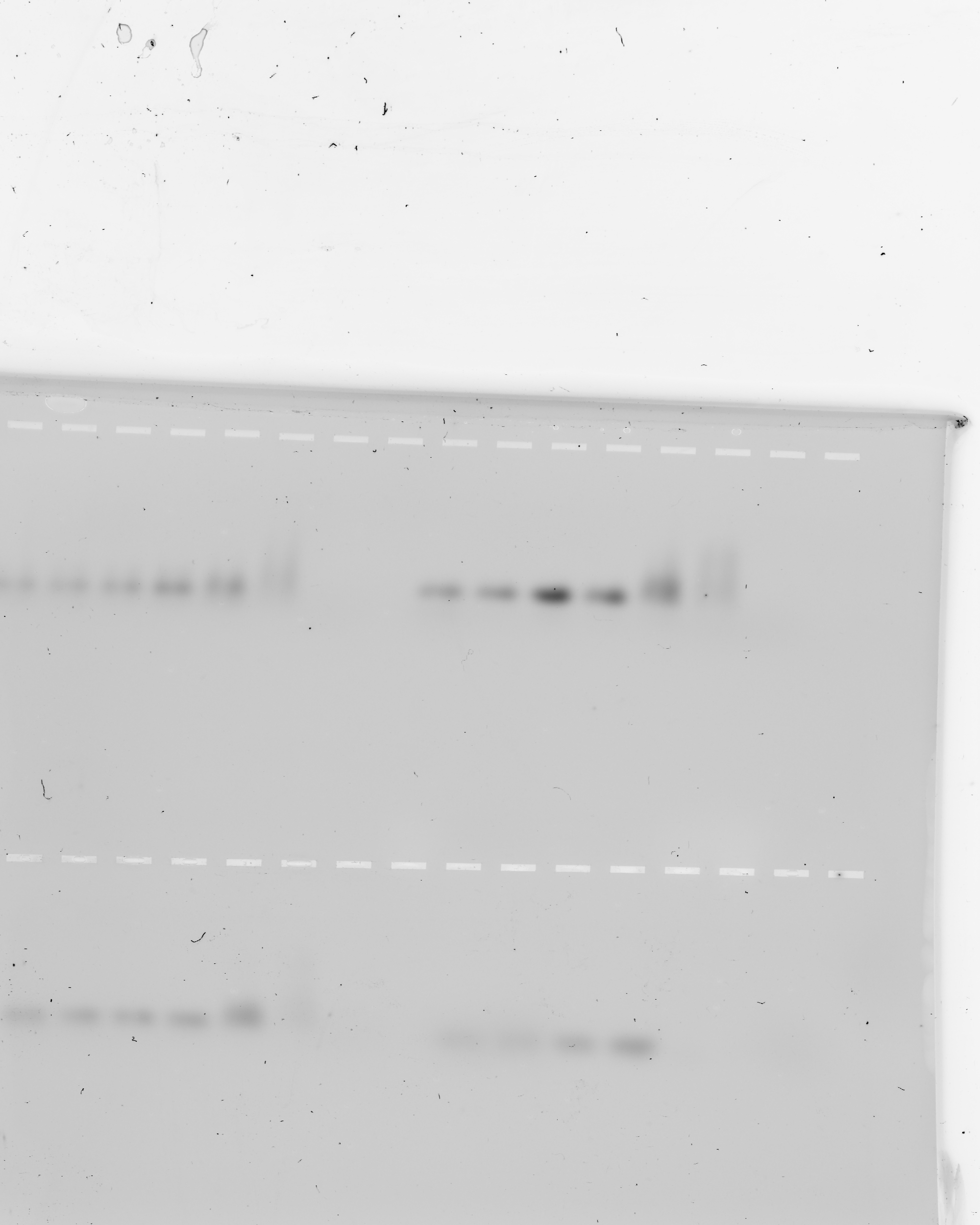

Supplement: Supplementary file 7 — Source data Fig. 3 [file 44319_2025_605_MOESM7_ESM.zip › Figure 3/3G/ecHOP1-wHTH-WT_DNA-substrates-various_09Apr25-[Cy2]-A.tif]

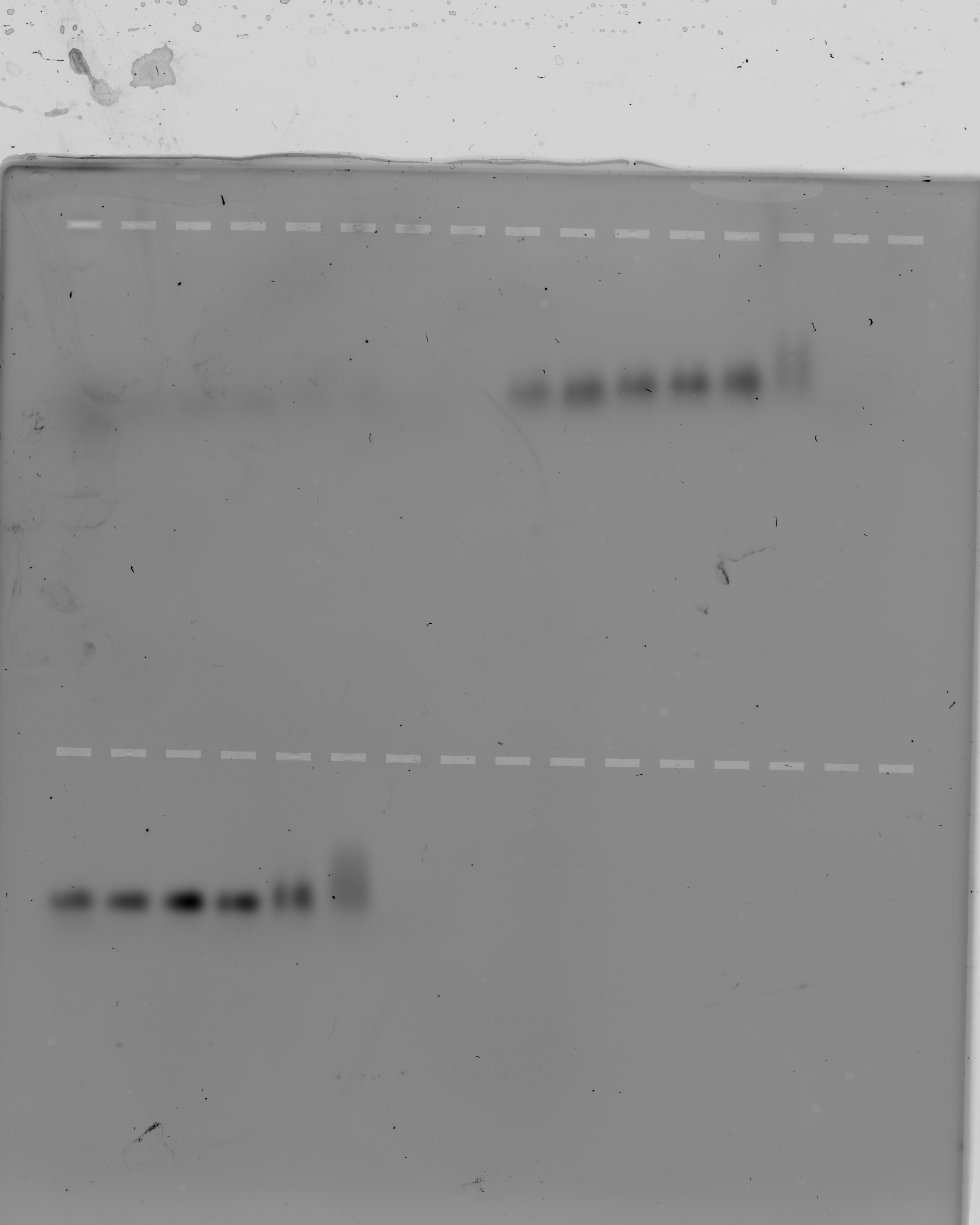

Supplement: Supplementary file 7 — Source data Fig. 3 [file 44319_2025_605_MOESM7_ESM.zip › Figure 3/3G/ecHOP1-wHTH-WT_DNA-substrates-various_09Apr25-[Cy2]-B.gel]

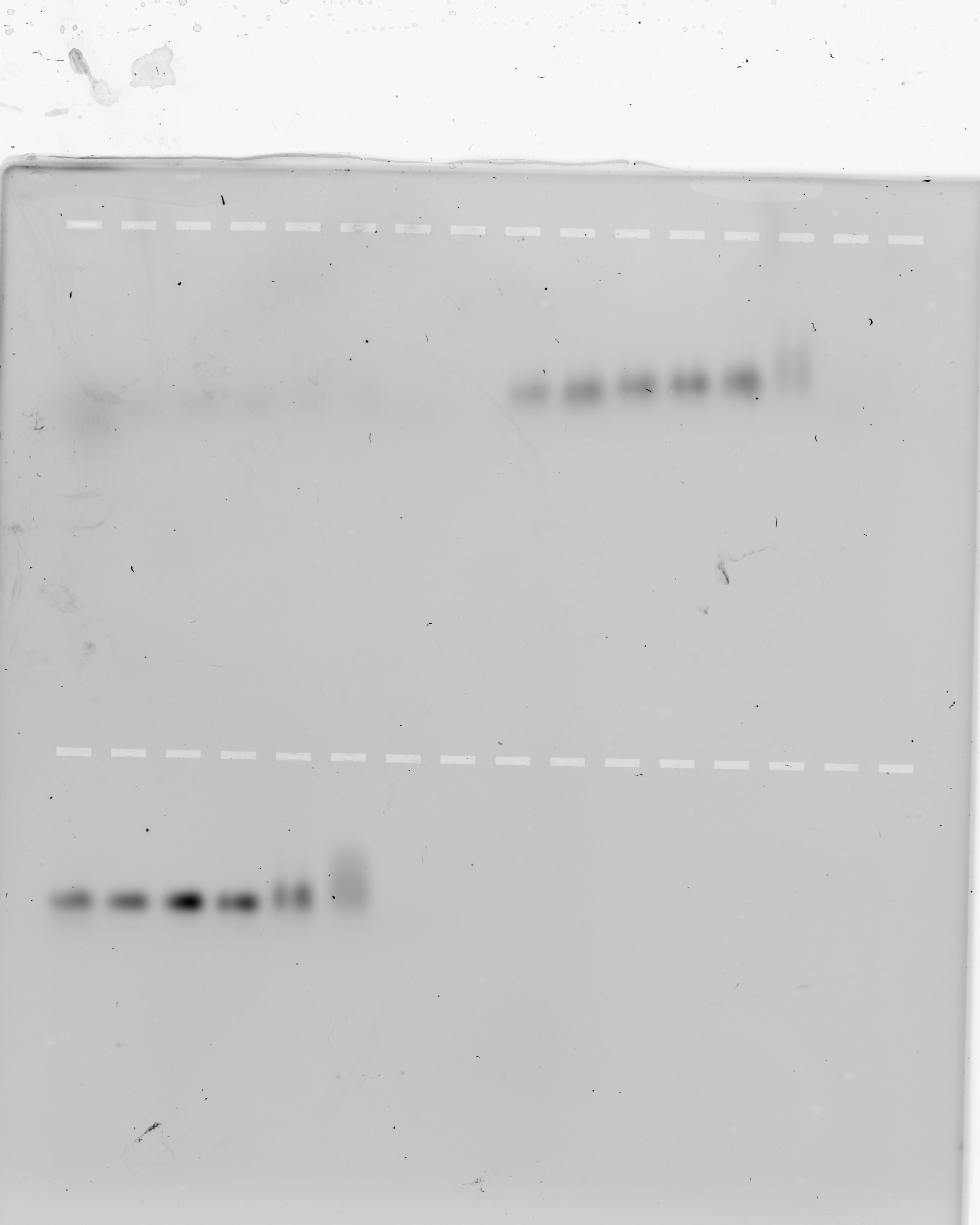

Supplement: Supplementary file 7 — Source data Fig. 3 [file 44319_2025_605_MOESM7_ESM.zip › Figure 3/3G/ecHOP1-wHTH-WT_DNA-substrates-various_09Apr25-[Cy2]-B.tif]

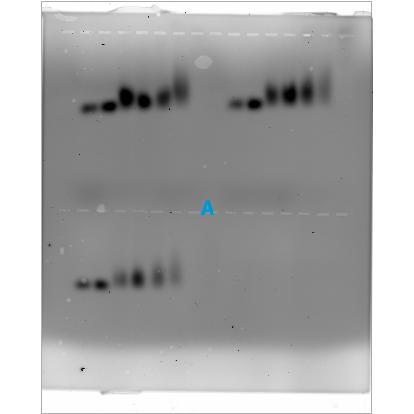

Supplement: Supplementary file 7 — Source data Fig. 3 [file 44319_2025_605_MOESM7_ESM.zip › Figure 3/3G/Figure3G_His6SUMO-ecHOP1-wHTH-WT_3'-overhang_Triplicates.jpg]

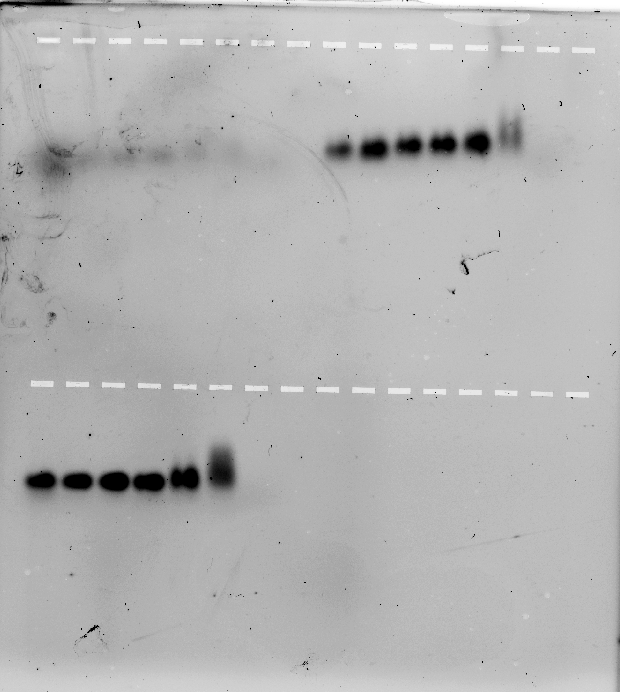

Supplement: Supplementary file 7 — Source data Fig. 3 [file 44319_2025_605_MOESM7_ESM.zip › Figure 3/3G/Figure3G_His6SUMO-ecHOP1-wHTH-WT_DNA-Substrates_3'-overhang_5'-overhang-dual-load.tif]

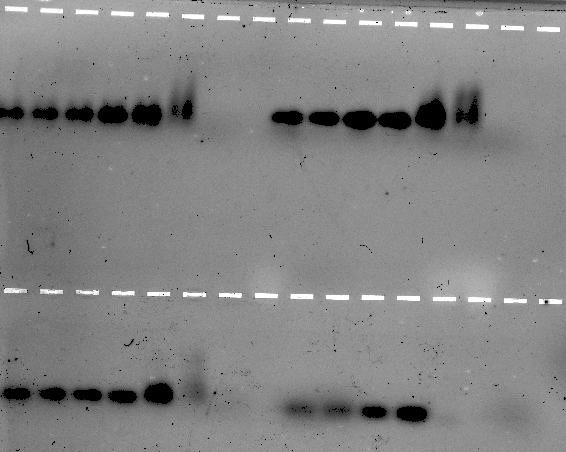

Supplement: Supplementary file 7 — Source data Fig. 3 [file 44319_2025_605_MOESM7_ESM.zip › Figure 3/3G/Figure3G_His6SUMO-ecHOP1-wHTH-WT_DNA-Substrates_D-loop_HJ_Y-form_Gel1.tif]

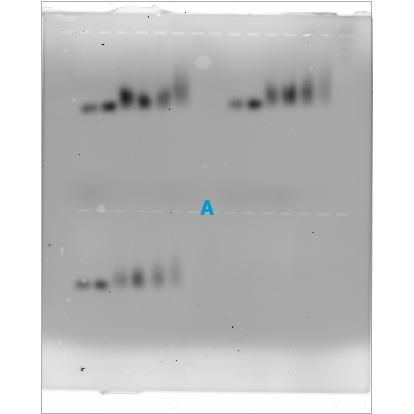

Supplement: Supplementary file 7 — Source data Fig. 3 [file 44319_2025_605_MOESM7_ESM.zip › Figure 3/3G/gel2.jpg]

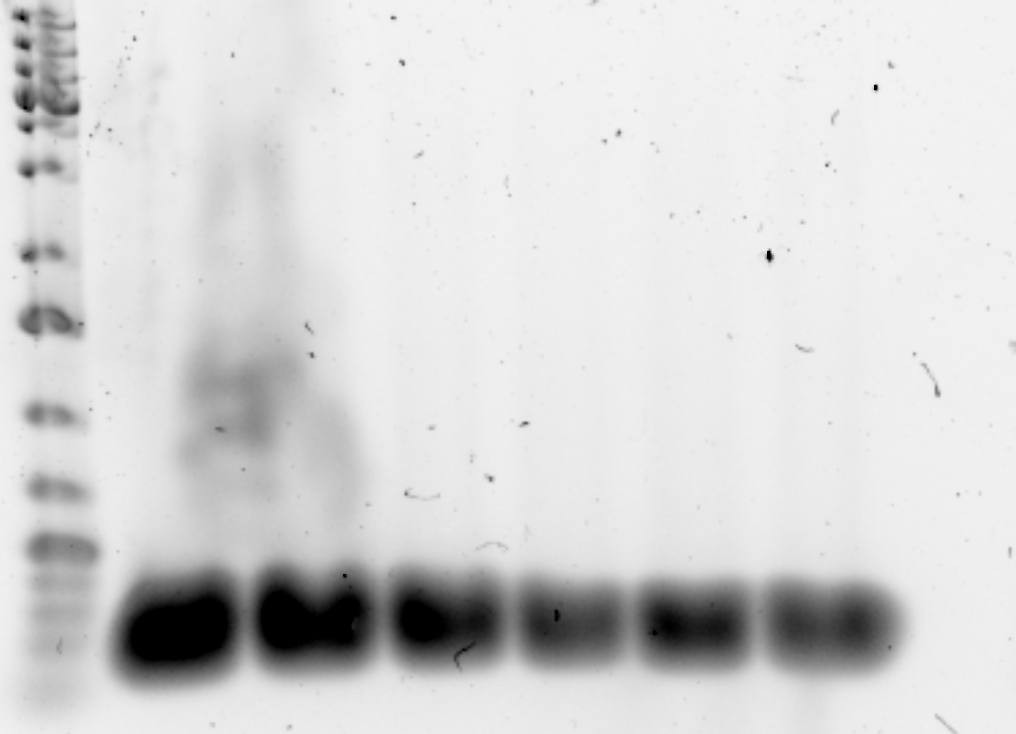

Supplement: Supplementary file 7 — Source data Fig. 3 [file 44319_2025_605_MOESM7_ESM.zip › Figure 3/3I/Figure3I_SUMO-wHTH-R432A_167bp-WIDOM_EMSA_Test1-inverted.tif]

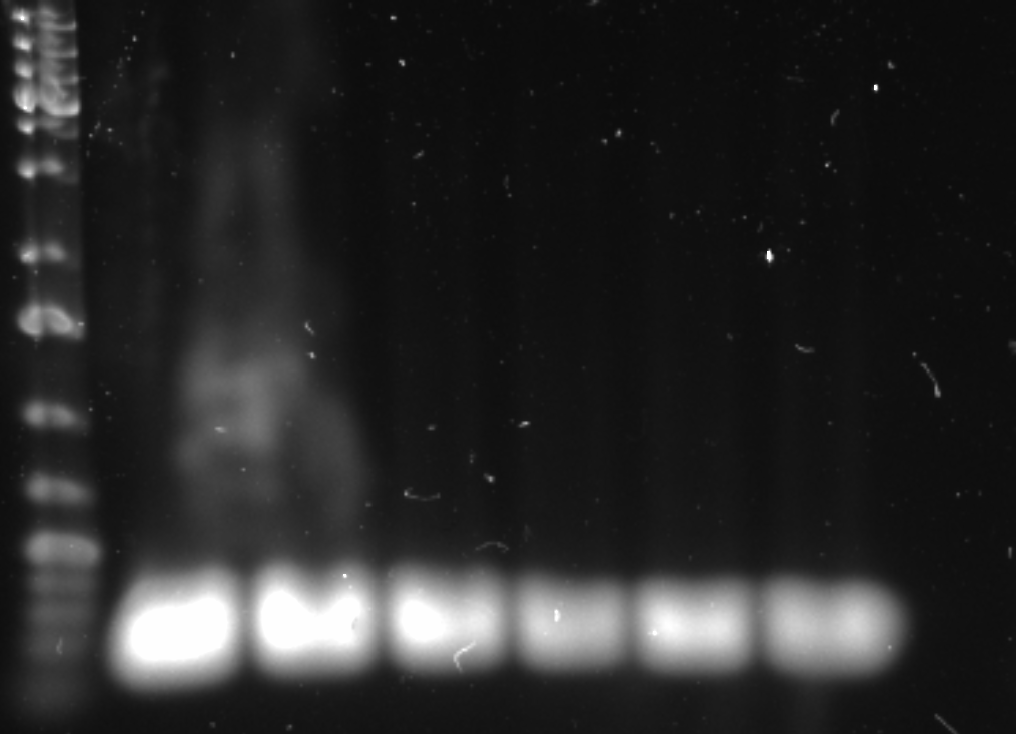

Supplement: Supplementary file 7 — Source data Fig. 3 [file 44319_2025_605_MOESM7_ESM.zip › Figure 3/3I/Figure3I_SUMO-wHTH-R432A_167bp-WIDOM_EMSA_Test1-original.tif]

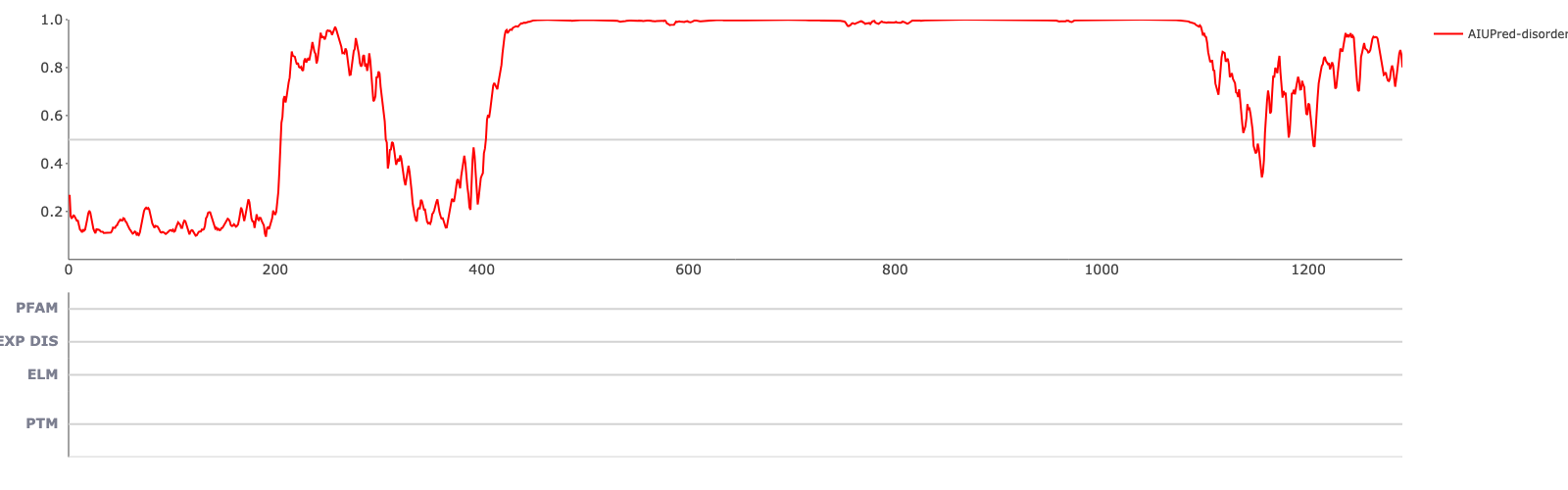

Supplement: Supplementary file 8 — Source data Fig. 4 [file 44319_2025_605_MOESM8_ESM.zip › Figure 4/4A/Figure4A_ecRED1-FL_AIUPred-Prediction.png]

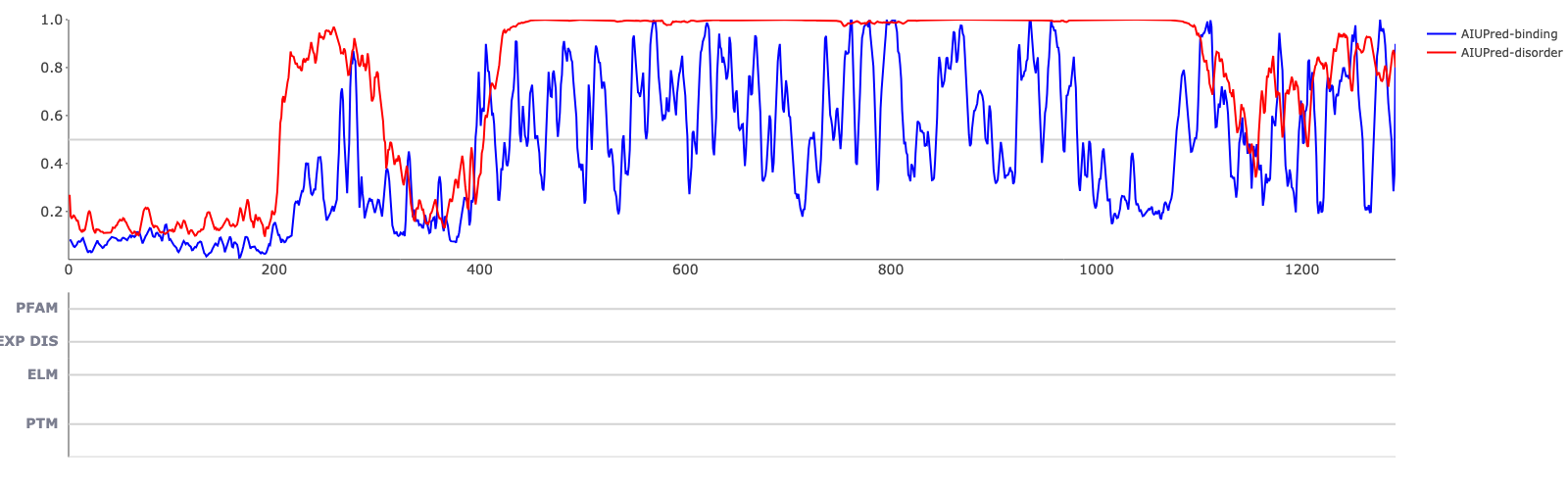

Supplement: Supplementary file 8 — Source data Fig. 4 [file 44319_2025_605_MOESM8_ESM.zip › Figure 4/4A/Figure4A_ecRED1_FL_IUPred-and ANCHOR2-Plots_plot.png]

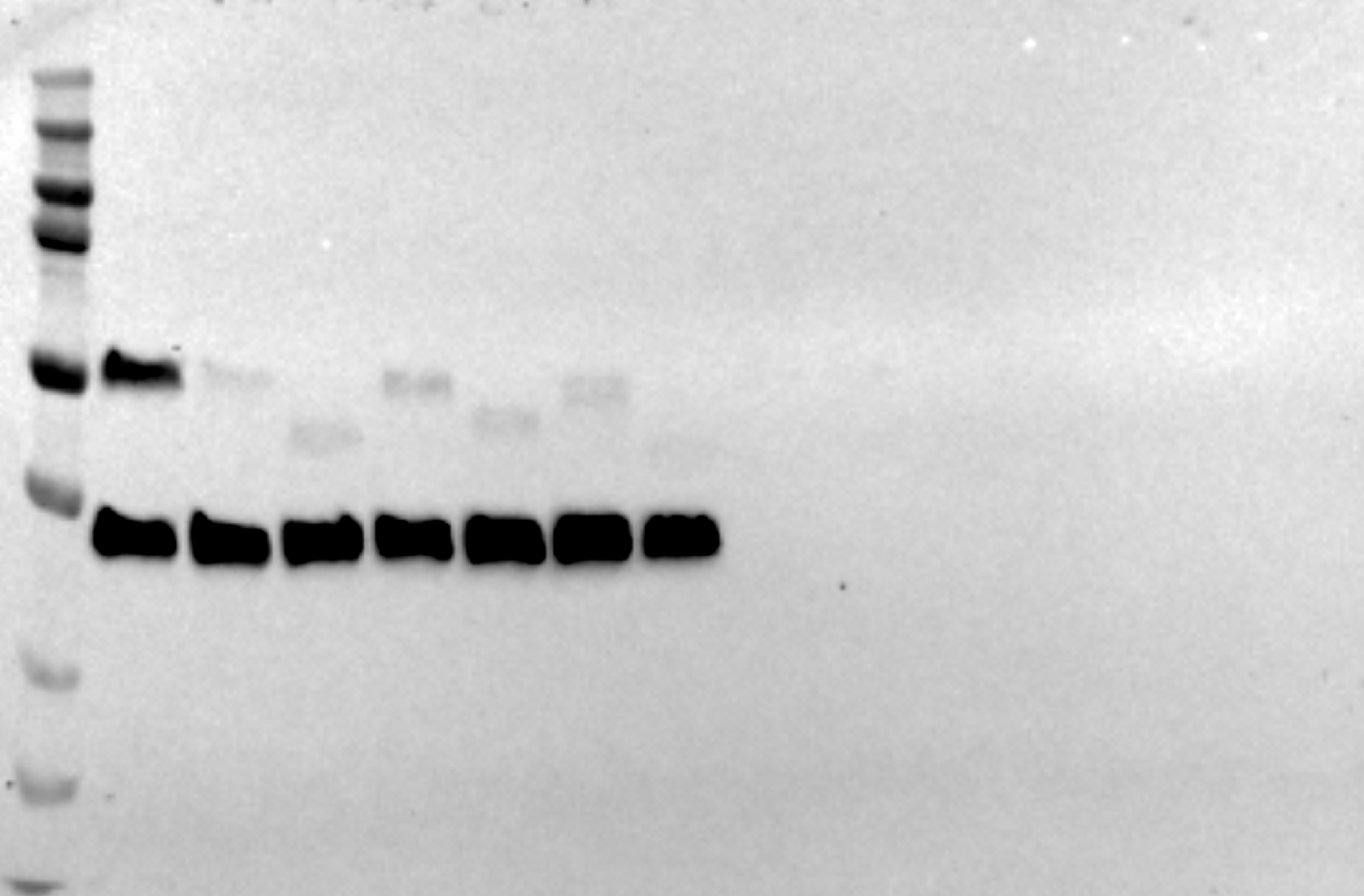

Supplement: Supplementary file 8 — Source data Fig. 4 [file 44319_2025_605_MOESM8_ESM.zip › Figure 4/4C/Figure4C_StrepII-EcHOP1-HORMA_His6MBP-ecRED1-CMs-all_Pull-Down_anti-Strep-anti-MBP_Gel4-Elute-High-Exposure-Merge.tif]

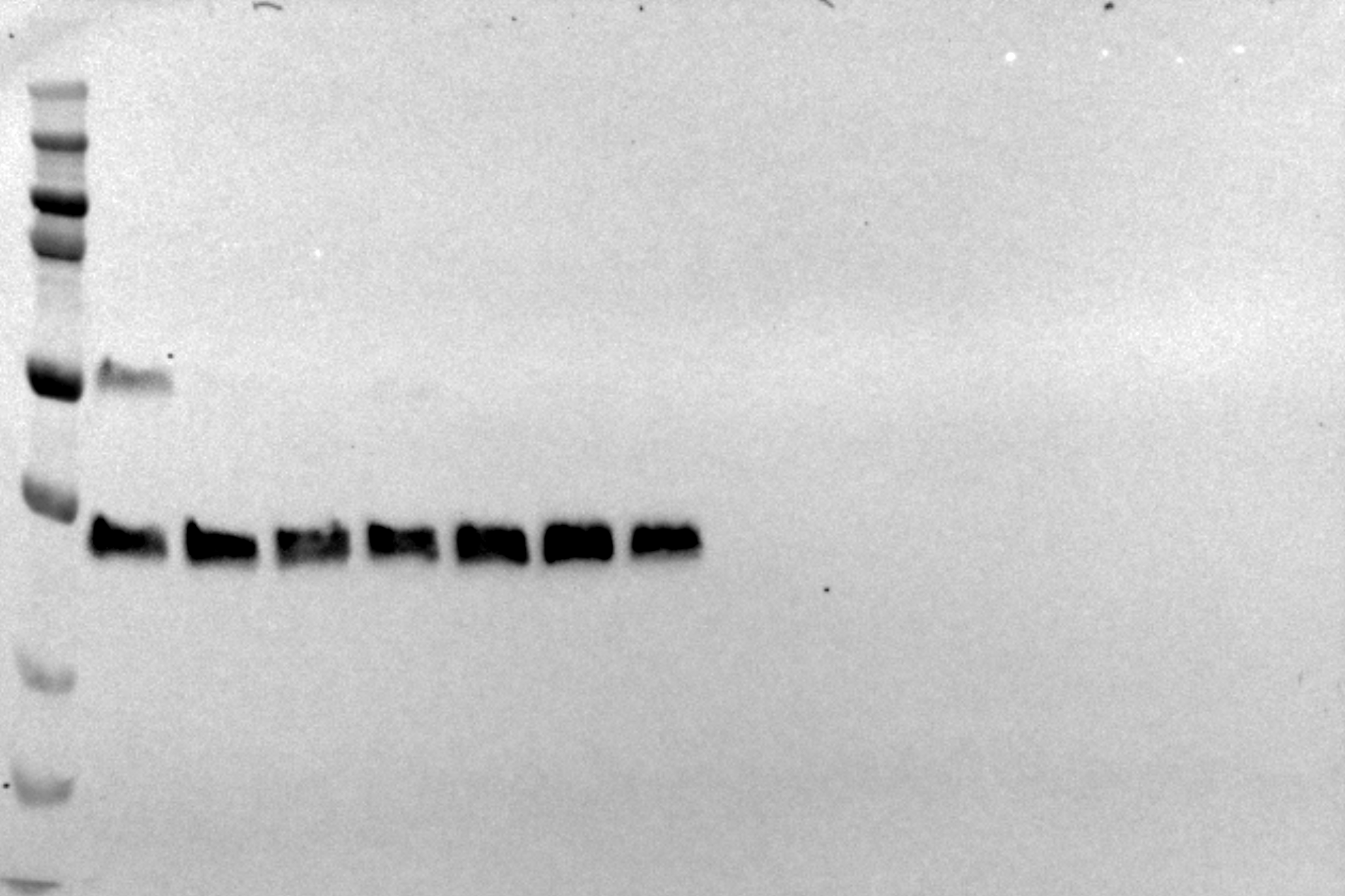

Supplement: Supplementary file 8 — Source data Fig. 4 [file 44319_2025_605_MOESM8_ESM.zip › Figure 4/4C/Figure4C_StrepII-EcHOP1-HORMA_His6MBP-ecRED1-CMs-all_Pull-Down_anti-Strep-anti-MBP_Gel4-Elute-Merge.tif]

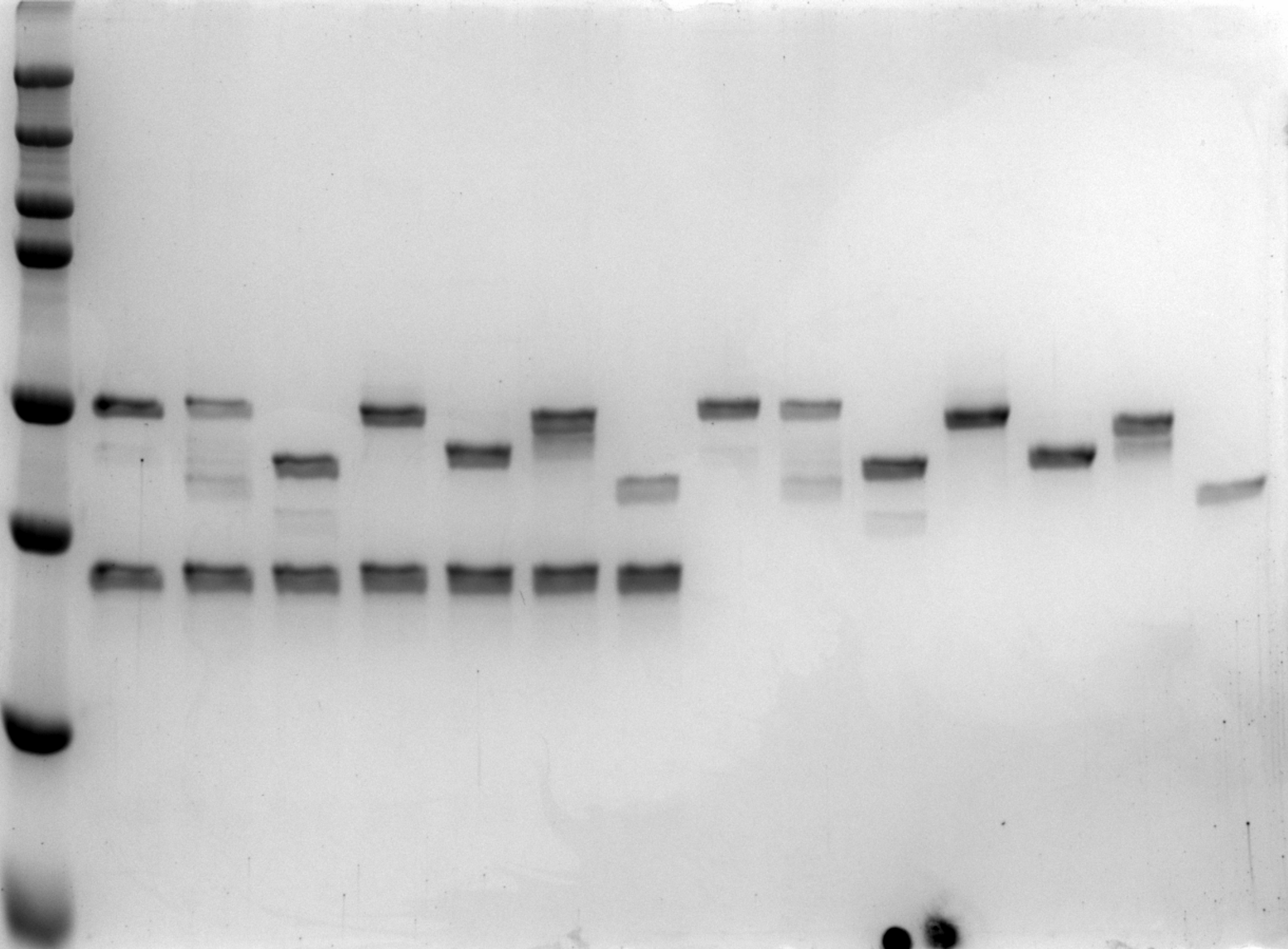

Supplement: Supplementary file 8 — Source data Fig. 4 [file 44319_2025_605_MOESM8_ESM.zip › Figure 4/4C/Figure4C_StrepII-EcHOP1-HORMA_His6MBP-ecRED1-CMs-all_Pull-Down_Gel1-Input-coomassie.tif]

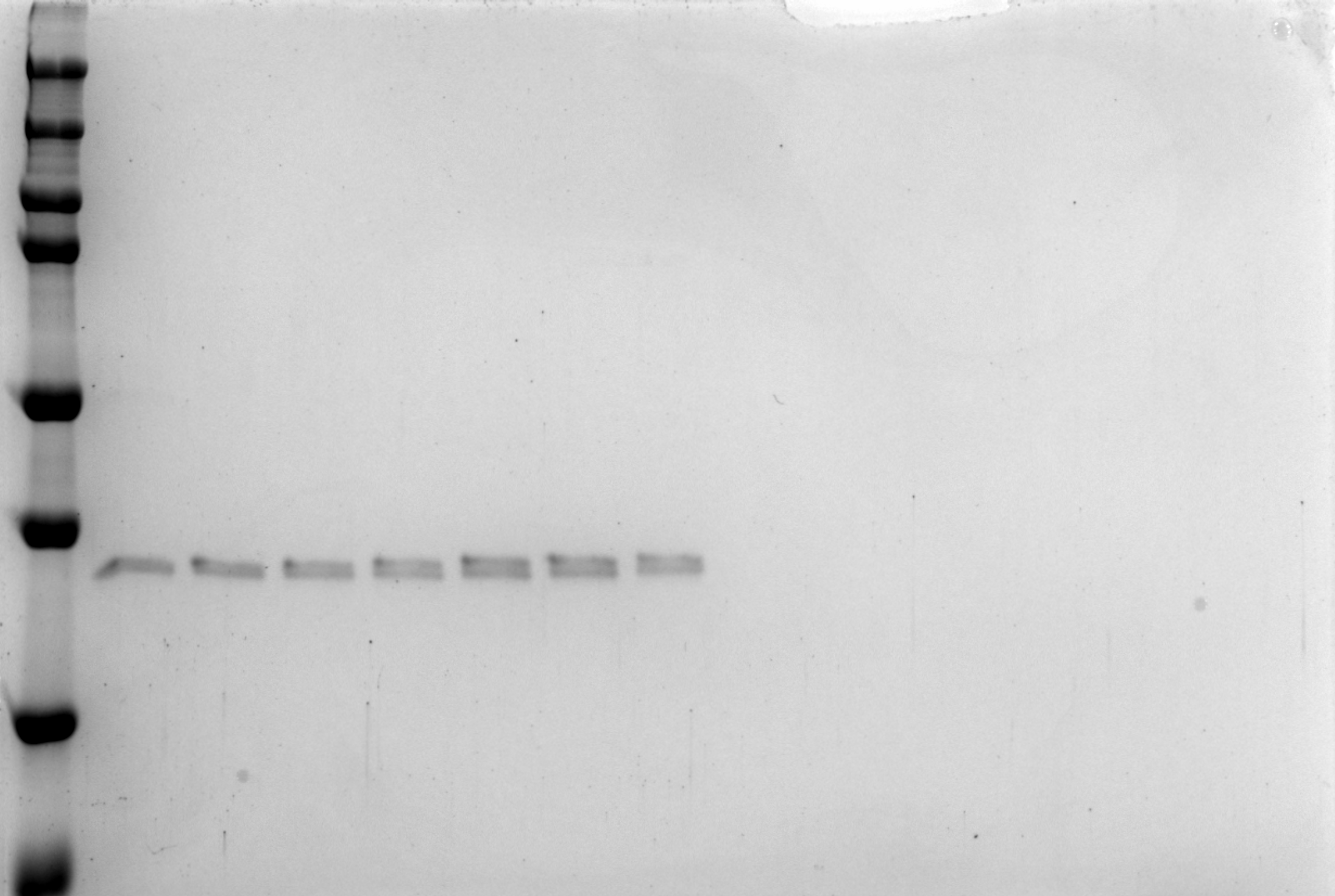

Supplement: Supplementary file 8 — Source data Fig. 4 [file 44319_2025_605_MOESM8_ESM.zip › Figure 4/4C/Figure4C_StrepII-EcHOP1-HORMA_His6MBP-ecRED1-CMs-all_Pull-Down_Gel4-Elution-coomassie.tif]

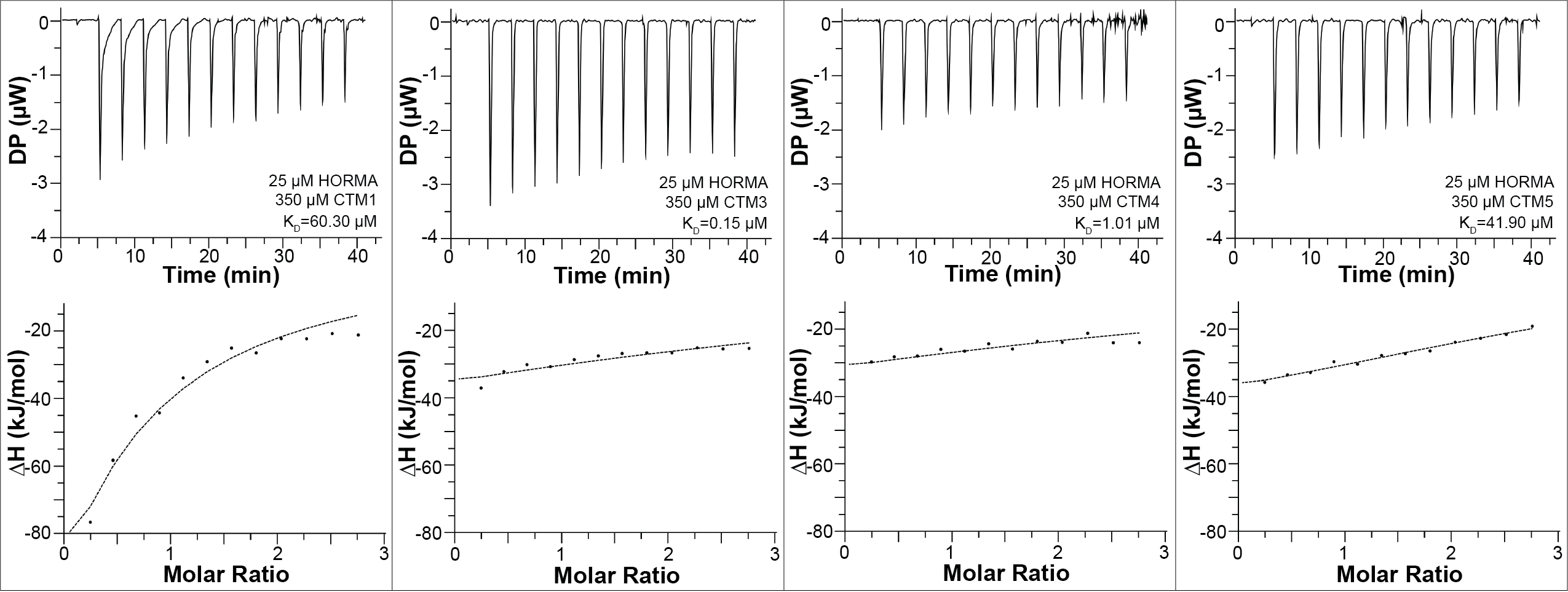

Supplement: Supplementary file 8 — Source data Fig. 4 [file 44319_2025_605_MOESM8_ESM.zip › Figure 4/4D/Figure4D_HORMA-25uM_ecRed1-CM-R-A_and-others-350-uM_Graphs.png]

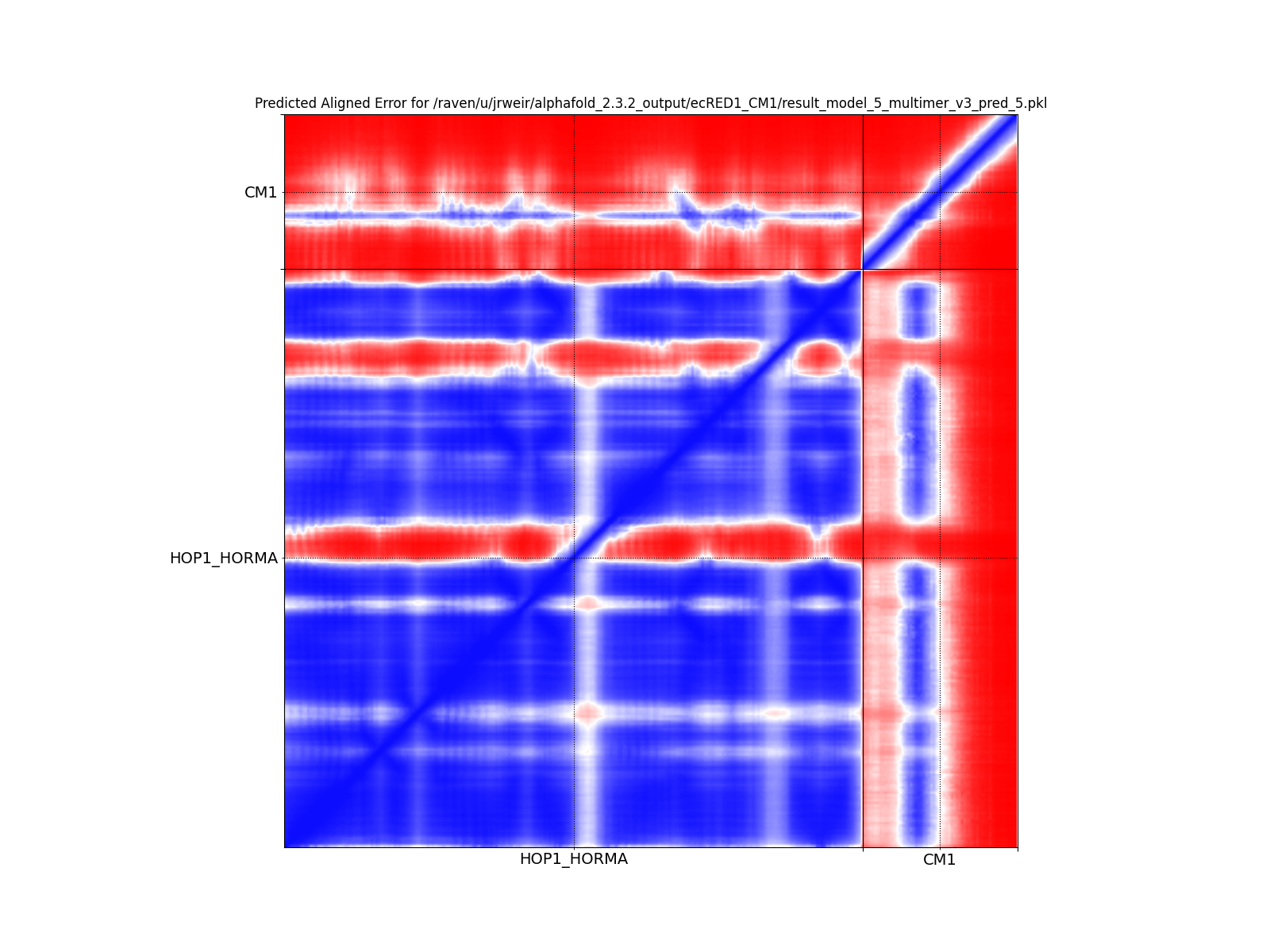

Supplement: Supplementary file 8 — Source data Fig. 4 [file 44319_2025_605_MOESM8_ESM.zip › Figure 4/4E/pae_ranked_0.png]
