# Supplementary material for: Characterization of meiotic axis proteins in the model brown alga Ectocarpus
Source: EMBO Rep. 2025 Oct 23;26(23):5673–702. doi: 10.1038/s44319-025-00605-3 (PMC12678776; doi:10.1038/s44319-025-00605-3)
Supplement: Supplementary file 6 — Source data Fig. 2 [file 44319_2025_605_MOESM6_ESM.zip › Figure 2/2B/Report-EcHOP1-HORMA-2_12Jul23.pdf]

ASTRA Report Experiment3

File Properties

Name: Experiment3

Sample: EcHOP1-2

Concentration: 2.800 mg/mL

Configuration

Concentration Source: RI
Flow Rate: 0.300 mL/min

Light Scattering Instrument: miniDAWN TREOS

Band Broadening Correction: Yes (Instrumental: 0.601 µL, Mixing: 53.692 µL)
Cell Type: Fused Silica
Wavelength: 658.0 nm
Calibration Constant: 5.0280×10^{-5} 1/(V cm)

Detector	Refractive Index Corrected Scattering Angle	Gain	Normalization Coefficient
1 (49.00)	43.63°	n/a	0.722
2 (90.00)	90.00°	n/a	1.000
3 (131.00)	136.37°	n/a	0.767

RI Instrument: Optilab rEX

Band Broadening Correction: n/a
Wavelength: 658.0 nm

UV Instrument: Generic UV

Band Broadening Correction: Yes (Instrumental: 15.743 µL, Mixing: 61.453 µL)
UV Cell Length: 1.000 cm

Solvent: Tris

Temperature Correction Enabled: yes
Refractive Index: 1.331

Fluid Connections

Source Instrument	Destination Instrument	Delay Volume (mL)
Generic Pump	Injector	0.000
Injector	Generic Column	0.000
Generic Column	Generic UV Instrument	0.000
Generic UV Instrument	miniDAWN TREOS	0.045
miniDAWN TREOS	Optilab rEX	0.093

Aux Connections

Source Instrument	Destination Instrument	Source Aux Channel	Destination Aux Channel	Calibration Constant
Generic UV Instrument	miniDAWN TREOS		2	1.000

Processing

Collection Time: Wednesday July 12, 2023 03:21:10 PM +0200
Processing Time: Wednesday July 12, 2023 03:42:36 PM +0200

Basic Collection:

LS Instrument Collection Interval: 0.500 sec

Baselines:

Series	Start	Stop	Type
detector 1	(1.352, 0.026)	(11.694, 0.025)	manual x, auto y
detector 2	(1.359, 0.010)	(11.682, 0.010)	manual x, auto y
detector 3	(0.971, 0.022)	(11.634, 0.022)	manual x, auto y
channel	(0.386, 0.050)	(11.995, 0.050)	manual x, auto y
differential refractive index data	(0.068, 0.000)	(11.359, 0.000)	manual x, auto y

Peak settings:

**Peak Name**	Peak 1	Peak 2
**Peak Limits (min)**	6.148 - 6.440	5.346 - 5.705
**Light Scattering Model**	Zimm	Zimm
**Fit Degree**	1	1
**dn/dc (mL/g)**	0.1850	0.1850
**A2 (mol mL/g²)**	0.000	0.000
**UV Ext. Coef. (mL/(mg cm))**	0.667	0.667

Results

Peak Results

	**Peak 1**	**Peak 2**
**Masses**		
**Injected Mass (µg)**	140.00	140.00
**Calculated Mass (µg)**	5.11	0.88
**Mass Recovery (%)**	3.7	0.6
**Mass Fraction (%)**	85.3	14.7
**Molar mass moments (g/mol)**		
**Mn**	4.053×10^{4} (±4.610%)	1.233×10^{5} (±12.312%)
**Mp**	3.758×10^{4} (±4.547%)	1.073×10^{5} (±13.370%)
**Mv**	n/a	n/a
**Mw**	4.085×10^{4} (±4.608%)	1.250×10^{5} (±12.106%)
**Mz**	4.118×10^{4} (±10.303%)	1.266×10^{5} (±26.890%)
**Mz+1**	4.154×10^{4} (±16.475%)	1.282×10^{5} (±42.507%)
**M(avg)**	4.055×10^{4} (±0.770%)	1.243×10^{5} (±1.832%)
**Polydispersity**		
**Mw/Mn**	1.008 (±6.518%)	1.013 (±17.266%)
**Mz/Mn**	1.016 (±11.288%)	1.027 (±29.574%)
**rms radius moments (nm)**		
**rn**	21.0 (±33.0%)	31.6 (±39.5%)
**rw**	21.1 (±32.8%)	31.6 (±39.2%)
**rz**	21.1 (±32.6%)	31.6 (±38.9%)
**r(avg)**	21.1 (±5.4%)	31.5 (±5.9%)
