# Supplementary material for: Characterization of meiotic axis proteins in the model brown alga Ectocarpus
Source: EMBO Rep. 2025 Oct 23;26(23):5673–702. doi: 10.1038/s44319-025-00605-3 (PMC12678776; doi:10.1038/s44319-025-00605-3)
Supplement: Supplementary file 9 — Expanded View Figures [file 44319_2025_605_MOESM9_ESM.pdf]

# Expanded View Figures

**Figure EV1. MSA ecHOP1 transcriptional isoforms.**

(**A**) (top) Genome annotation figure of the two ecHOP1 isoforms from ORCAE. (bottom) Alignment between the two transcriptional isoforms of ecHOP1 (Ec-07_005800.1 for isoform #1 and Ec-07_005800.2 for isoform #2) show high conservation between the two. Interestingly, there is an insertion between a.a. 495-514 in isoform #2 (blue), as well as a *C*-terminal extension following a.a. 574 in isoform #1 (orange). Although the insertion in isoform #2 does not computationally demonstrate to influence the protein structure, the extension in isoform #1 contains the two putative CMs. The DNA sequences for each isoform were acquired through ORCAE, and the translated sequences were utilized for MSA. The MSA was generated with JalView and aligned with MAFFT using default settings. (**B**) Topology map of ecHOP1 HORMA domain, colored as in Fig. 2. (**C**) AlphaFold2 model of ecHOP1-HORMA alongside experimentally determined HORMA domain structures from nematodes (*C. elegans*; HTP-3 with HIM-2 CM (PDB: 4TZM)) and mammalians (*H. sapiens* HORMAD1 bound in *cis* to HORMAD1 CM (PDB: 8J69)). (**D**) MSA of the brown algal HOP1 orthologs, highlighting the novel β3′′ region (residues 94-107 in ecHOP1). A color gradient from light to dark was implemented to indicate the percentage of conservation. The alignment was generated using MAFFT and curated in Jalview.

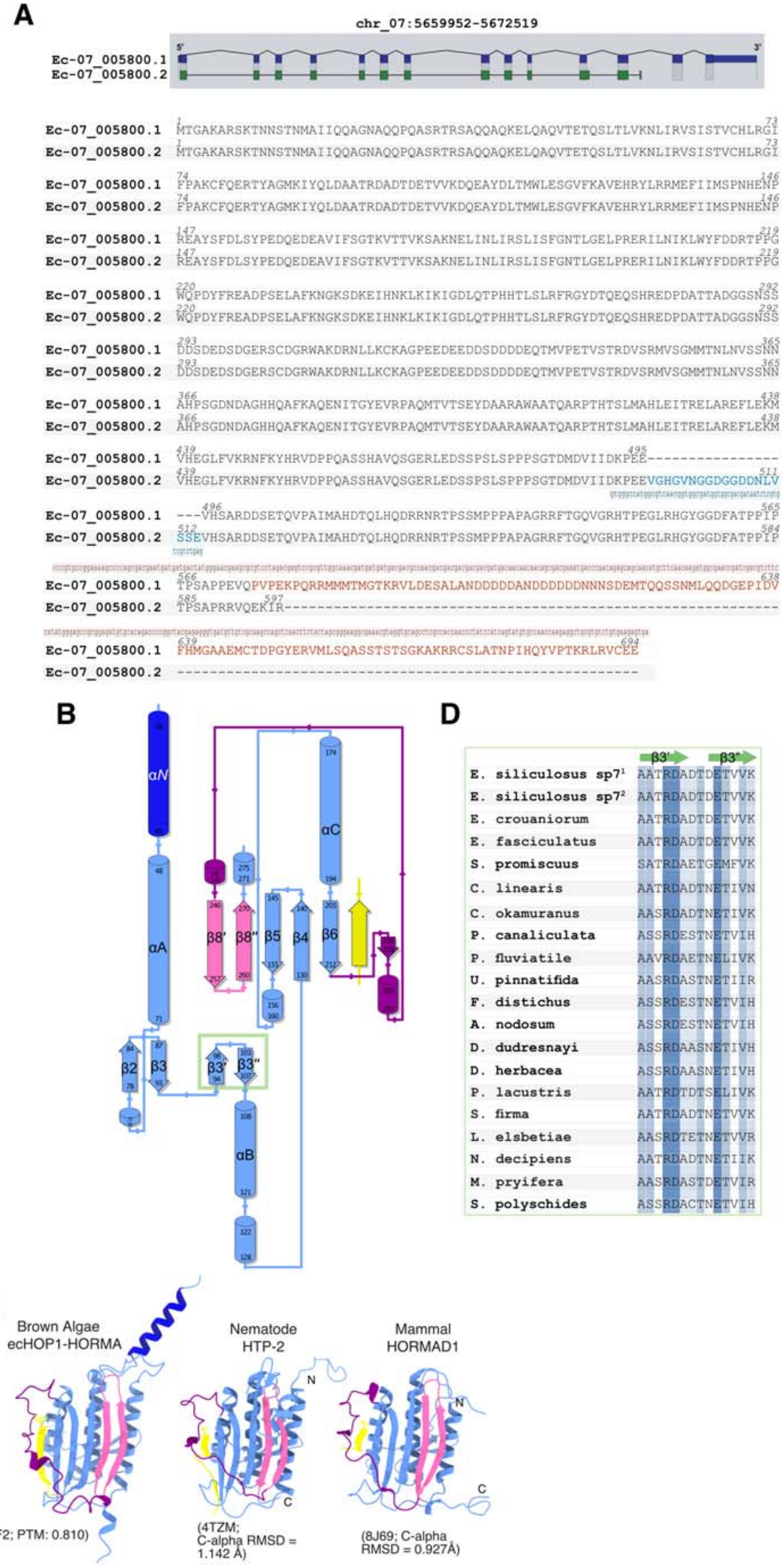
A
chr_07:5659952-5672519
Ec-07_005800.1
Ec-07_005800.2
5'
3'
Ec-07_005800.1 MTGAKARSKTNNSTNMAIIQQAGNAQQPQASRTRSAQQAQKELQAQVTETQSLTLVKNLIRVSISTVCHLRGI
Ec-07_005800.2 MTGAKARSKTNNSTNMAIIQQAGNAQQPQASRTRSAQQAQKELQAQVTETQSLTLVKNLIRVSISTVCHLRGI
Ec-07_005800.1 FPAKCFQERTYAGMKIYQLDAATRDADTDETVVKDQEAYDLTMWLESGVFKAVEHRYLRRMEFIIMSPNHENP
Ec-07_005800.2 FPAKCFQERTYAGMKIYQLDAATRDADTDETVVKDQEAYDLTMWLESGVFKAVEHRYLRRMEFIIMSPNHENP
Ec-07_005800.1 REAYSFDLSYPEDQEDEAVIFSGTKVTTVKSAKNELINLIRSLISFGNTLGELPRERILNIKLWYFDDRTPPG
Ec-07_005800.2 REAYSFDLSYPEDQEDEAVIFSGTKVTTVKSAKNELINLIRSLISFGNTLGELPRERILNIKLWYFDDRTPPG
Ec-07_005800.1 WQPDYFREADPSELAFKNGKSDKEIHNKLKIKIGDLQTPHHTLSLRFRGYDTQEQSHREDPDATTADGGSNSS
Ec-07_005800.2 WQPDYFREADPSELAFKNGKSDKEIHNKLKIKIGDLQTPHHTLSLRFRGYDTQEQSHREDPDATTADGGSNSS
Ec-07_005800.1 DDSDEDSDGERSCDGRWAKDRNLLKCKAGPEEDEEDDSDDDDEQTMVPETVSTRDVSRMVSGMMTNLNVSSNN
Ec-07_005800.2 DDSDEDSDGERSCDGRWAKDRNLLKCKAGPEEDEEDDSDDDDEQTMVPETVSTRDVSRMVSGMMTNLNVSSNN
Ec-07_005800.1 AHPSGDNDAGHHQAFKAQENITGYEVRPAQMTVTSEYDAARAWAATQARPTHTSLMAHLEITRELAREFLEKM
Ec-07_005800.2 AHPSGDNDAGHHQAFKAQENITGYEVRPAQMTVTSEYDAARAWAATQARPTHTSLMAHLEITRELAREFLEKM
Ec-07_005800.1 VHEGLFVKRNFKYHRVDPPQASSHAVQSGERLEDSSPSLSPPPSGTDMDVIIDKPEE
Ec-07_005800.2 VHEGLFVKRNFKYHRVDPPQASSHAVQSGERLEDSSPSLSPPPSGTDMDVIIDKPEEVGHGVNGGDGGDDNLV
Ec-07_005800.1 VHSARDDSETQVPAIMAHDTQLHQDRRNRTPSSMPPPAPAGRRFTGQVGRHTPEGLRHGYGGDFATPPIP
Ec-07_005800.2 SSEVHSARDDSETQVPAIMAHDTQLHQDRRNRTPSSMPPPAPAGRRFTGQVGRHTPEGLRHGYGGDFATPPIP
Ec-07_005800.1 TPSAPPEVQPVPEKPQRRMMMTMGTKRVLDESALANDDDDDANDDDDDDNNNSDEMTQQSSNMLQQDGEPIDV
Ec-07_005800.2 TPSAPRRVQEKIR
Ec-07_005800.1 FHMGAAEMCTDPGYERVMLSQASSTSTSGKAKRRCSLATNPIHQYVPTKRLRVCEE
Ec-07_005800.2
B
αN
αA
β2
β3
β3'
β3''
αB
β8'
β8''
β5
β4
αC
β6
D
β3'
β3''
E. siliculosus sp7[1] AATRDADTDETVVK
E. siliculosus sp7[2] AATRDADTDETVVK
E. crouaniorum AATRDADTDETVVK
E. fasciculatus AATRDADTDETVVK
S. promiscuus SATRDAETGEMFVK
C. linearis AATRDADTNETIVN
C. okamuranus AASRDADTNETIVK
P. canaliculata ASSRDESTNETVIH
P. fluviatile AAVRDAETNELIVK
U. pinnatifida AASRDASTNETIIR
F. distichus ASSRDESTNETVIH
A. nodosum ASSRDESTNETVIH
D. dudresnayi ASSRDAASNETIVH
D. herbacea ASSRDAASNETIVH
P. lacustris AATRDTDTSELIVK
S. firma AATRDADTNETVVK
L. elsbetiae AASRDTETNETVVR
N. decipiens AATRDADTNETIIK
M. pryifera AASRDASTDETVIR
S. polyschides ASSRDACTNETVIH
C
Brown Algae
ecHOP1-HORMA
Nematode
HTP-2
Mammal
HORMAD1
N
C
(AF2; PTM: 0.810)
(4TZM;
C-alpha RMSD =
1.142 Å)
(8J69; C-alpha
RMSD = 0.927Å)

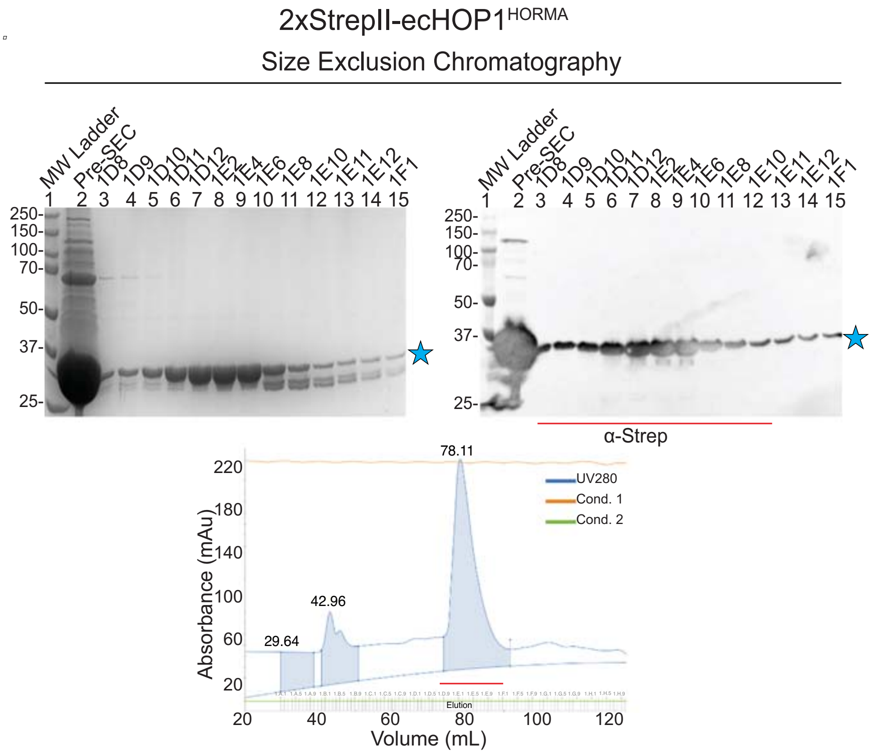


**Figure EV2. Purification of ecHOP1-HORMA.**

Purification of 2xStrepII-ecHOP1-HORMA. The Coomassie-stained gel image and western blot shown here also appear in Fig. 2A.

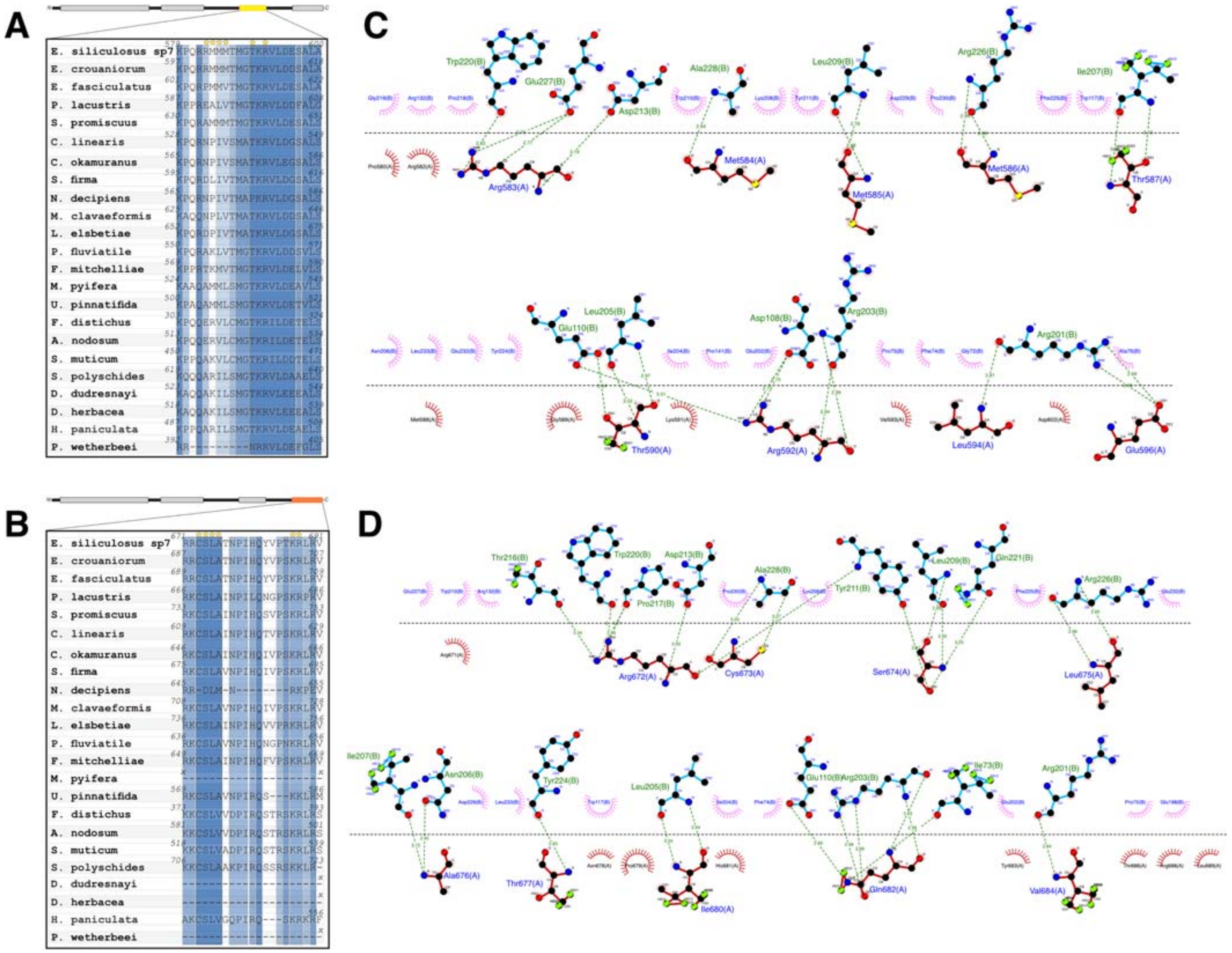


**Figure EV3. Details of CM-H1-A and CM-H1-B.**

(**A**) MSA of CM-H1-A with other sequences from within Stramenopiles. Residues predicted to be involved in the interaction with the HORMA domain are indicated with yellow stars. (**B**) MSA of CM-H1-B with other sequences from within Stramenopiles. Residues predicted to be involved in the interaction with the HORMA domain are indicated with yellow stars. (**C**) LigPlot analysis of the top-rated (based on iPTM) prediction of CM-H1-A with ecHOP1-HORMA. (**D**) LigPlot analysis of the top-rated (based on iPTM) prediction of CM-H1-B with ecHOP1-HORMA.

 © The Author(s)

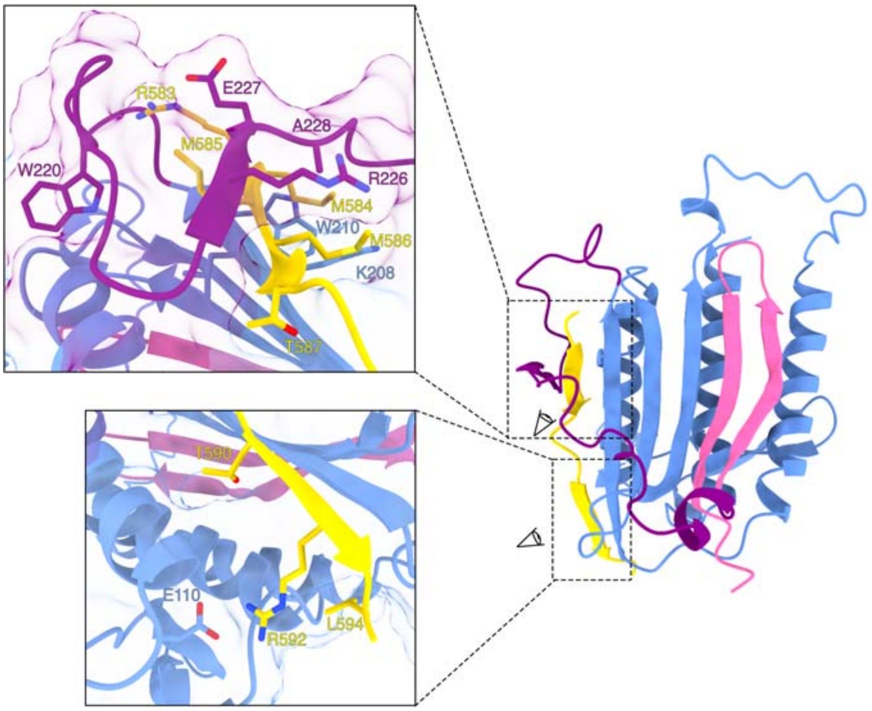


**Figure EV4. Structural analysis of the predicted interaction of CM-H1-A with ecHOP1-HORMA.**

AlphaFold2 model of ecHOP1-HORMA in complex with CM-H1-A (yellow), highlighting key intermolecular interactions. (top) Close-up of the CM-H1-A residues R583, M584, M585, and M588 engaging ecHOP1-HORMA core residues K208 and W210 (blue), as well as the conserved ecHOP1-HORMA residues within the safety belt region R226, W220, E227, and A228 (purple). R583 is predicted to form side-chain H-bonds with E227 and the main chain of W220, while the methionine cluster forms hydrophobic contacts with surrounding residues. (bottom) Additional contacts between CM-H1-A residues T590, R592, and L594 (yellow) and ecHOP1-HORMA residue E110 (blue), which is conserved and situated outside the safety belt.

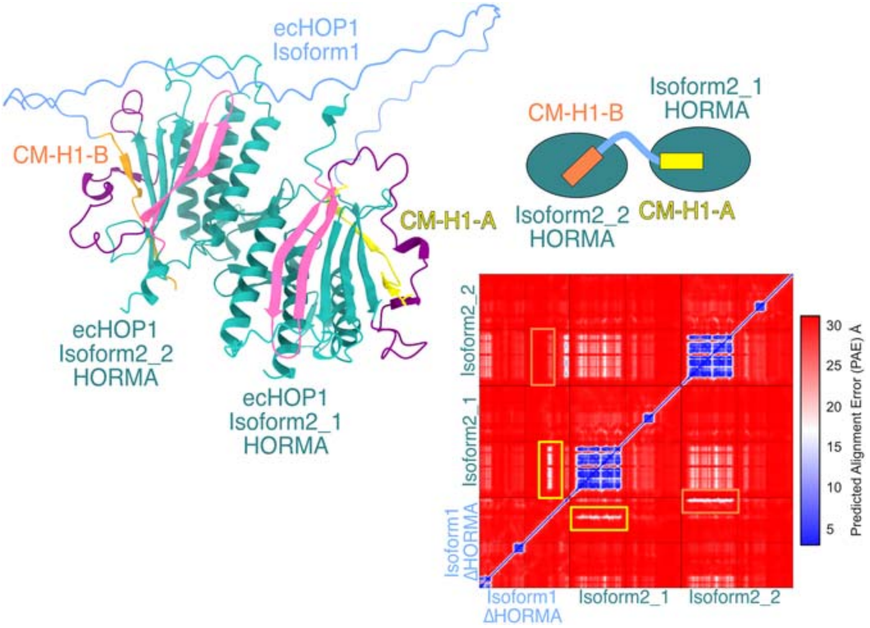


**Figure EV5. ecHOP1-isoform 1 could bind two ecHOP1-isoform 2.**

AlphaFold2 model of a complex of ecHOP1-isoform1 (with the *N*-terminal HORMA domain removed; residues 562–694) (blue; CM-H1-A, yellow; CM-H1-B, orange) and two copies of full-length ecHOP1-isoform2. The model is colored as elsewhere, but the ecHOP1-HORMA-isoform2 domains are colored in teal. In the PAE plot, the CMs are highlighted with the colors as in the cartoon.

 © The Author(s)

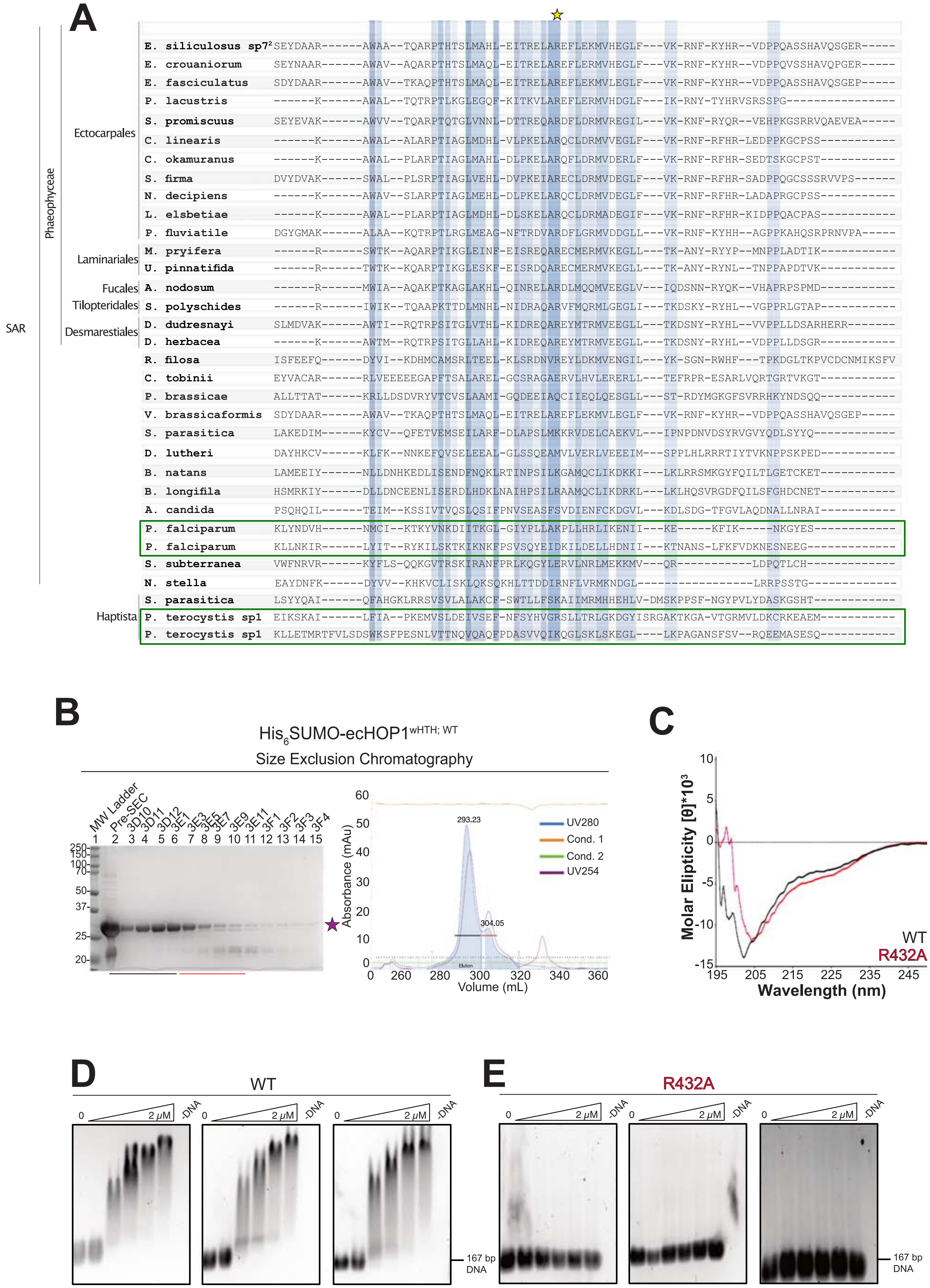
A
SAR
Phaeophyceae
Ectocarpales
Laminariales
Fucales
Tilopteridales
Desmarestiales
Haptista
E. siliculosus sp7[2]
E. crouaniorum
E. fasciculatus
P. lacustris
S. promiscuus
C. linearis
C. okamuranus
S. firma
N. decipiens
L. elsbetiae
P. fluviatile
M. pryifera
U. pinnatifida
A. nodosum
S. polyschides
D. dudresnayi
D. herbacea
R. filosa
C. tobinii
P. brassicae
V. brassicaformis
S. parasitica
D. lutheri
B. natans
B. longifila
A. candida
P. falciparum
P. falciparum
S. subterranea
N. stella
S. parasitica
P. terocystis sp1
P. terocystis sp1
B
His6SUMO-ecHOP1wHTH; WT
Size Exclusion Chromatography
MW Ladder
Pre-SEC
3D10
3D11
3D12
3E1
3E3
3E5
3E7
3E9
3E11
3F1
3F2
3F3
3F4
1 2 3 4 5 6 7 8 9 10 11 12 13 14 15
250-
150-
100-
70-
50-
37-
25-
20-
Absorbance (mAu)
Volume (mL)
293.23
304.05
UV280
Cond. 1
Cond. 2
UV254
C
Molar Elipticity [θ]*10³
Wavelength (nm)
WT
R432A
D
WT
0
2 μM
-DNA
167 bp DNA
E
R432A
0
2 μM
-DNA
167 bp DNA

**Figure EV6. ecHOP1-wHTH and DNA binding properties.**

(**A**) MSA of HOP1 wHTH within SAR and Haptista clades. The two species with twin wHTH domains are highlighted (green boxes). (**B**) Purification of the $His_6$SUMO-ecHOP1-wHTH. (**C**) Circular dichroism spectra of ecHOP1-wHTH WT (black) and R432A mutant (red). The wHTH domain is composed of three α-helices, a β-loop, and a flexible wing, typically producing minima near 208, 217, and 222 nm. Similar spectra for WT and R432A indicate that the mutation does not significantly alter the secondary structure or overall fold of the domain. (**D**) Triplicate EMSAs of WT ecHOP1-wHTH on 167 bp dsDNA. The first of these EMSAs is shown in Fig. 3I. (**E**) Triplicate EMSAs of ecHOP1-wHTH R432A mutant on 167 bp dsDNA. The first of these EMSAs is shown in Fig. 3I.


© The Author(s)

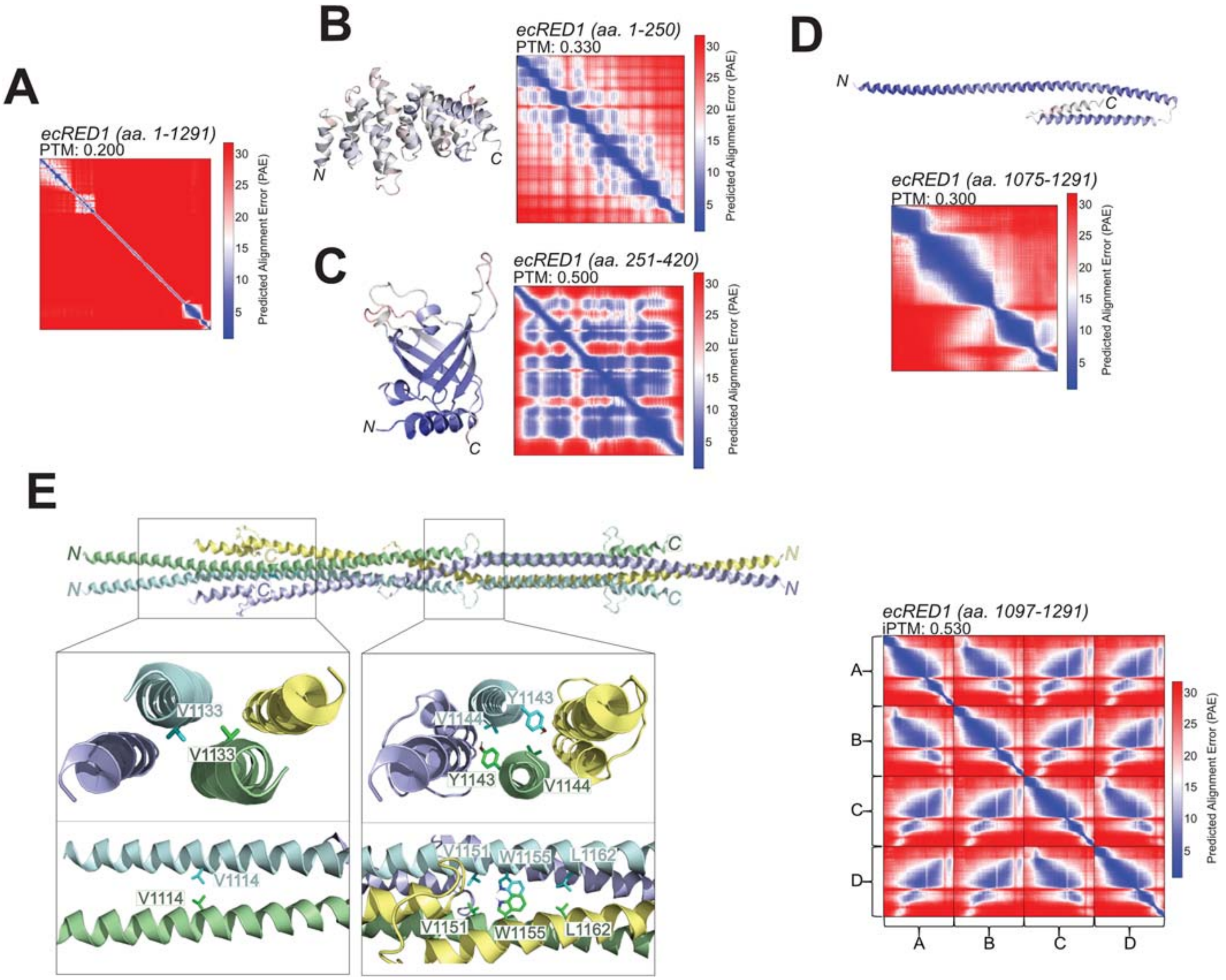


**Figure EV7. ecRED1 structure modeling and validation.**

(**A–D**) AlphaFold2 model of ecRED1 domains with PAE plots. (**E**) AlphaFold2 model of ecRED1 CC domain as a tetramer with PAE plot.

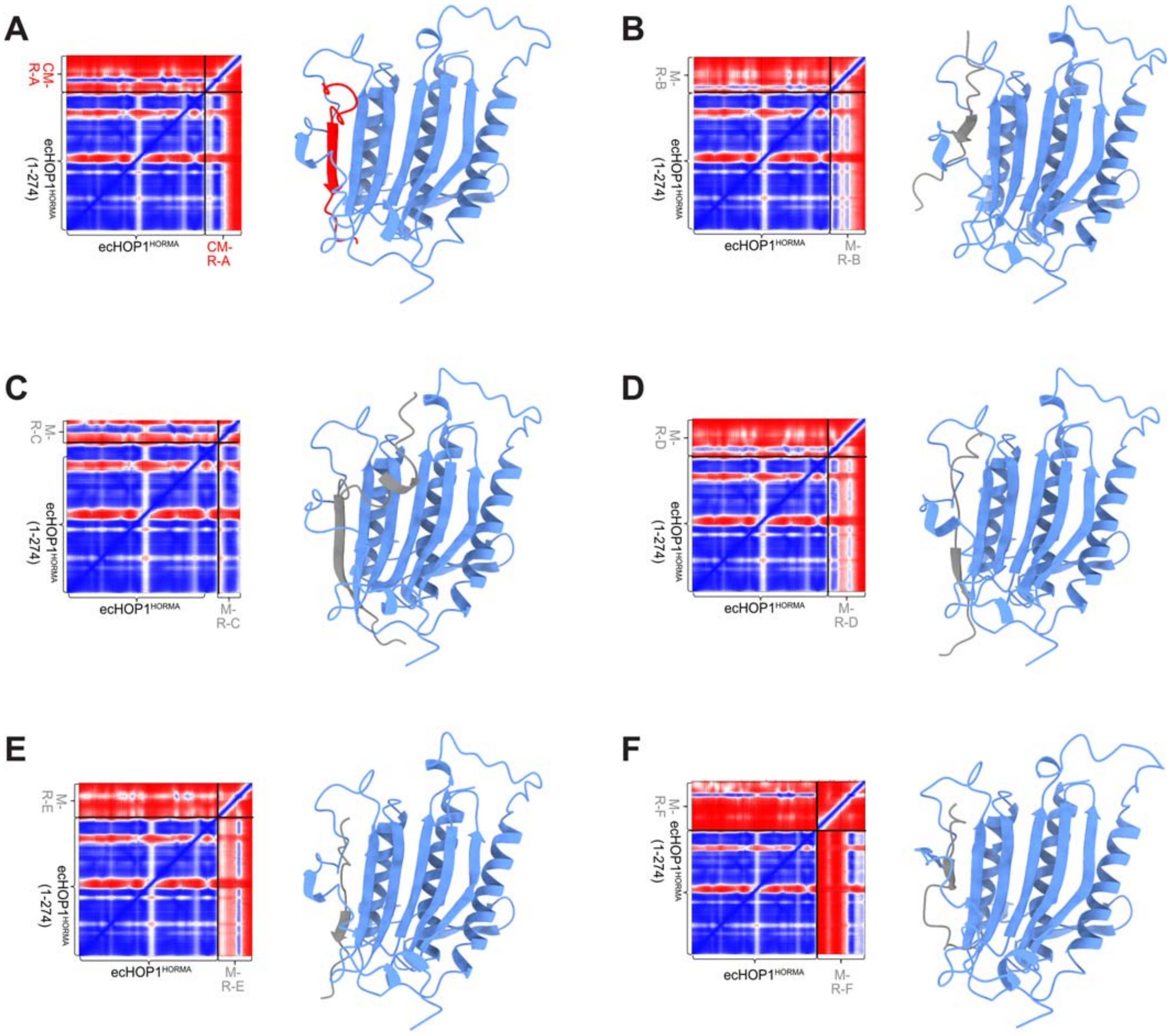


**Figure EV8. PAE plots and models of ecHOP1-HORMA with ecRED1 candidate closure motifs.**

(**A**) PAE plot, colored as in Fig. 4E. Cartoon representation of ecHOP1-HORMA in blue, CM-R-A in red. (**B–F**) PAE plot, colored as in Fig. 4E. Cartoon representation of ecHOP1-HORMA in blue, M-R-B to M-R-F in gray.

 © The Author(s)

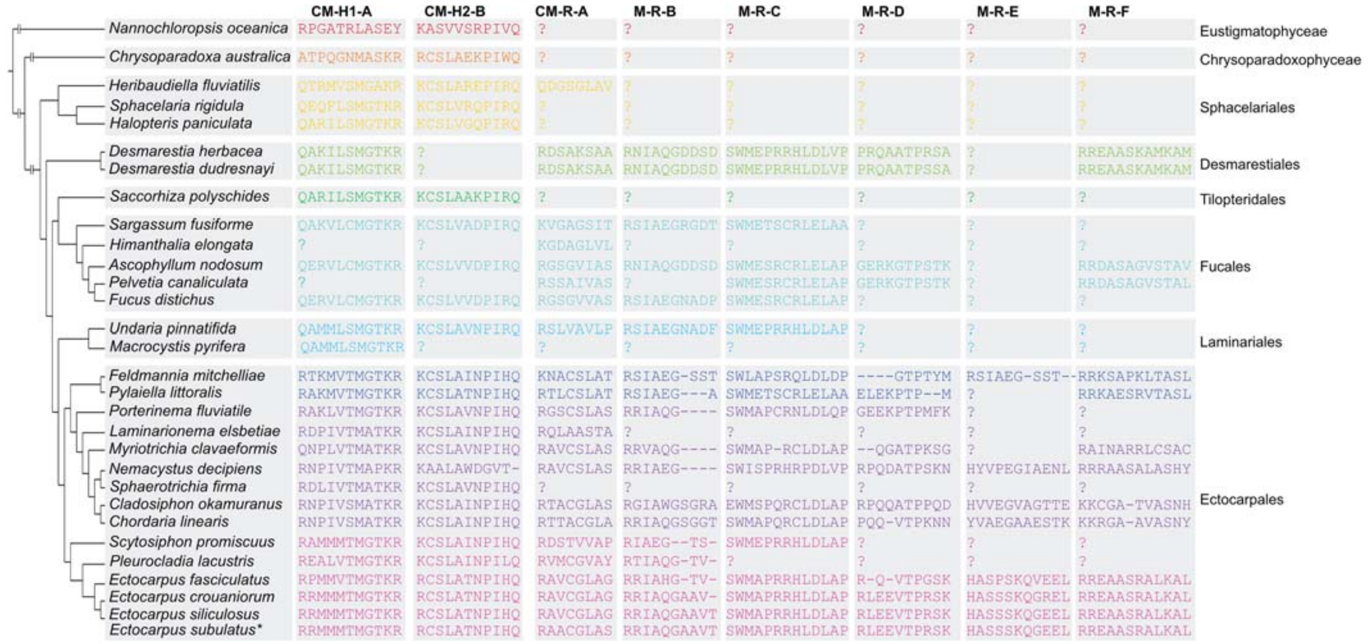


**Figure EV9. Closure motif phylogeny.**

MSA and phylogenetic analysis of brown algal HOP1 and RED1 orthologs reveal a lineage-specific rise of CMs. Absence of specific CMs in certain organisms is due to poor genome annotations or lack of conservation in relevant regions. In Ectocarpales, ecHOP1 and ecRED1 CMs are highly conserved, with additional ecRED1 CMs emerging in Desmarestiales. HOP1 orthologs in Eustigmatophyceae and Chrysoparadoxophyceae show two well-conserved CMs, suggesting an early addition of CMs for regulatory functions. The alignment was generated using MAFFT and curated in Jalview to identify conserved lysine and arginine residues defining CMs.

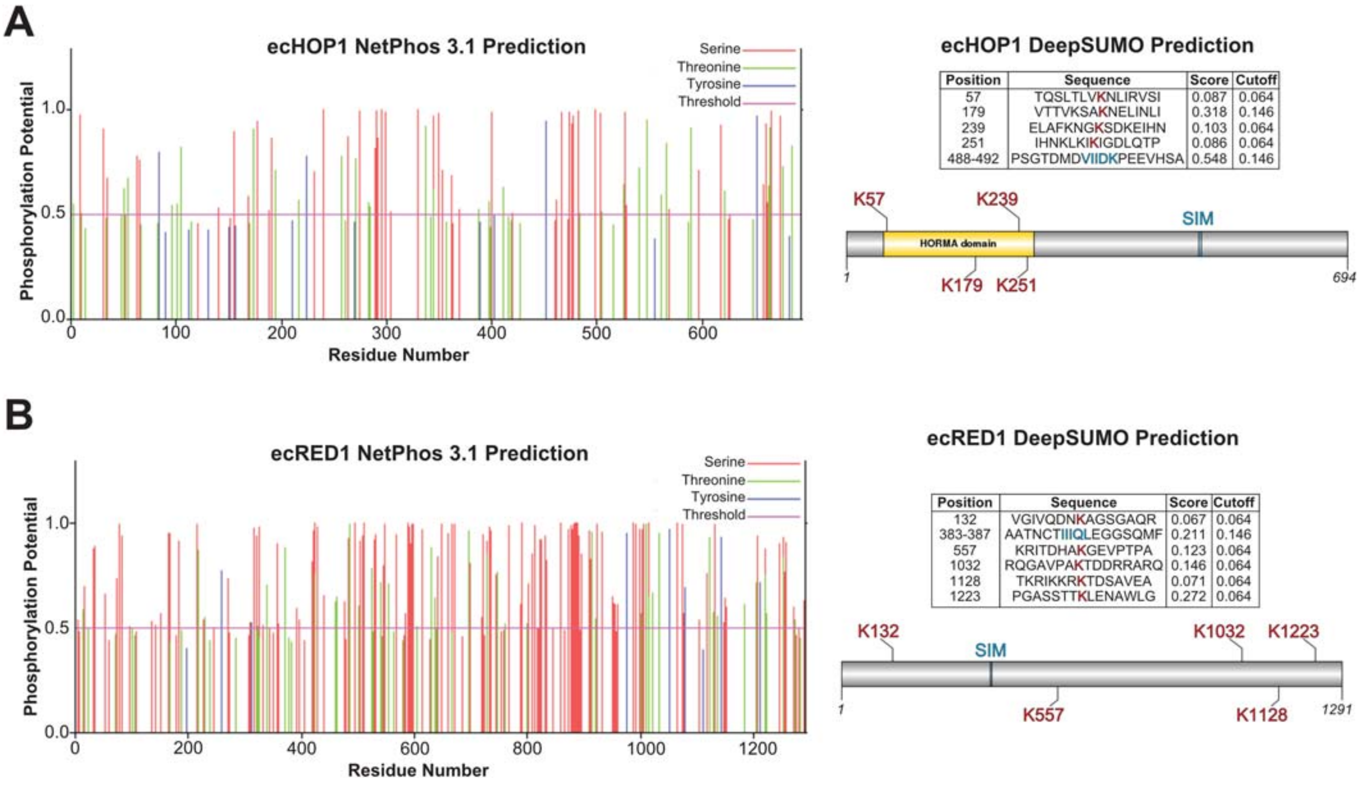


**Figure EV10. Potential post-translational modifications on ecHOP1 and ecRED1.**

(**A**) Predicted phosphorylation and SUMOylation sites mapped onto the AlphaFold3 model of ecHOP1. (**B**) Predicted phosphorylation and SUMOylation sites mapped onto the AlphaFold3 model of ecRED1. Phosphorylation sites (serine, threonine, tyrosine) and candidate SUMOylation motifs, including SUMO interaction motifs (SIMs), were predicted in silico using NetPhos 3.1 and DeepSUMO, respectively. Several predicted phosphorylation sites cluster near putative or non-functional CMs in ecRED1. Although multiple SUMOylation sites were identified, structural modeling did not support stable SUMO binding, suggesting possible regulation dependent on additional in vivo factors.

 © The Author(s)
